# Oxadiargyl analogs as potent inhibitors of *Toxoplasma gondii* protoporphyrinogen oxidase

**DOI:** 10.1039/d5md00888c

**Published:** 2025-11-03

**Authors:** Samuel Kwain, Vikky Awasthi, Rajib Islam, Shivani Kore, Emma Polaski, Kerrick C. Rees, Zhicheng Dou, Daniel C. Whitehead

**Affiliations:** a Department of Chemistry, Clemson University Clemson SC 29634 USA dwhiteh@clemson.edu; b Department of Biological Sciences, Clemson University Clemson SC 29634 USA zdou@clemson.edu; c Eukaryotic Pathogens Innovation Center, Clemson University Clemson SC 29634 USA

## Abstract

*Toxoplasma gondii* infects approximately one-third of the human population, posing a severe and potentially fatal risk to individuals with compromised immune systems. Our previous studies demonstrated that modifying the arene in the herbicidal protoporphyrinogen oxidase (PPO) inhibitor, oxadiazon, yields analogs that potently inhibit *T. gondii* PPO, a key enzyme in the heme biosynthesis pathway. In this study, we further investigated the structure–activity relationship of oxadiazon analogs by introducing aliphatic chains with varying functionalities, resulting in 23 new derivatives. Some of these compounds exhibited significant intracellular inhibition of wild-type *T. gondii*, with IC_50_ values ranging from 2 to 3 μM. Biochemical analysis confirmed that their mode of action is mediated by potent PPO inhibition, which further blocked heme production and damaged mitochondrial health status in the parasites. These findings enhance our understanding of oxadiazon's structural optimization and highlight its derivatives as promising early-stage candidates for developing effective therapies against toxoplasmosis in humans and other animals.

## Abstract 2

A click chemistry strategy allowed for the preparation of 23 oxadiargyl analogs exhibiting potent activity against the human pathogen, *Toxoplasma gondii*.

## Graphical abstract

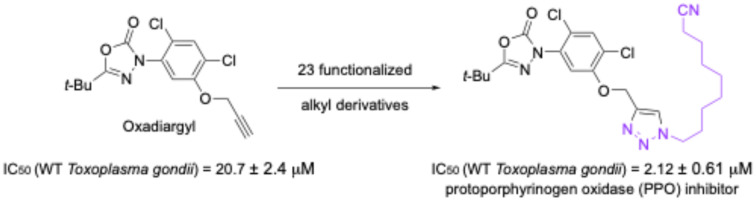

*Toxoplasma gondii*, an obligate intracellular protozoan belonging to the Apicomplexa phylum, is among the most widespread parasitic organisms, infecting approximately one-third of the global human population as well as a diverse range of other warm-blooded animals.^1–4^ While acute infections are generally asymptomatic in immunocompetent individuals, they can cause severe complications in immunocompromised individuals, pregnant women, and patients undergoing chemotherapy or organ transplants. In such cases, the infection may lead to encephalitis or neurological damage, which can be life-threatening.^4–12^ Current treatment options, such as pyrimethamine and sulfadiazine, are hindered by significant side effects and limited efficacy, particularly against chronic and congenital toxoplasmosis. This highlights the urgent need for novel therapeutic strategies.^13^ One promising avenue for drug development lies in targeting the parasite's unique heme biosynthesis pathway.^14–16^*T. gondii* synthesizes heme – an essential molecule for numerous cellular functions – *via* a plant-like pathway that is distinct from the pathways used by mammals.^16,17^ This divergence presents an attractive therapeutic target, as heme biosynthesis plays a critical role in the parasite's intracellular growth and acute virulence.^16^ Studies have demonstrated that heme-deficient parasites exhibit significantly reduced virulence and are effectively eliminated by the host's innate immune system.^16^ These findings strongly suggest that disrupting heme biosynthesis could offer an effective strategy for combating *T. gondii* infections.

One of the key enzymes in the heme biosynthesis pathway is protoporphyrinogen oxidase (PPO), which in *T. gondii* shows a closer phylogenetic relationship to plant PPOs than to those in mammals.^16^ PPO is a well-established target in herbicide development, where inhibitors cause the accumulation of toxic intermediates that destroy plant cell membranes.^18^ This similarity suggests that herbicidal PPO inhibitors, which have been effectively used for weed control with high specificity and low toxicity, could serve as starting molecular scaffolds for the development of new antiparasitic compounds.

Indeed, initial work from our group identified oxadiazon (1) and its propargyl homolog oxadiargyl (2) as moderately effective against WT *T. gondii* (Fig. 1).^16^ Building on this study, we explored the structure–activity relationship of the homolog, oxadiargyl, by appending an aryl ring of varying functionality *via* a straightforward “click” chemistry approach. Five out of the 18 oxadiargyl analogs prepared showed significantly improved potency, achieving IC_50_ values between 1.0–1.9 μM against *T. gondii* (Fig. 1).^19^ We demonstrated that the observed inhibitory activity of these compounds against wild-type *T. gondii* resulted primarily from PPO inhibition, as they were approximately 10 to 30 times less potent against a *Toxoplasma* PPO knockout mutant carrying a luciferase reporter, *Δppo::NLuc*. The inhibitory activity was restored when the strain was complemented with *Toxoplasma* PPO (*ΔppoPPO::NLuc*).^19^ Due to the promising results from the derivatization of the arene in compounds 3–7, we elected to further explore a similar “click” chemistry-based strategy to modify oxadiargyl (2) with aliphatic motifs bearing various functionality. This study broadens our understanding of the structural drivers governing potent inhibition of *Toxoplasma* PPO and may inform the future development of an effective therapy against toxoplasmosis in humans and other animals by targeting heme biosynthesis in the parasite.

**Fig. 1:**
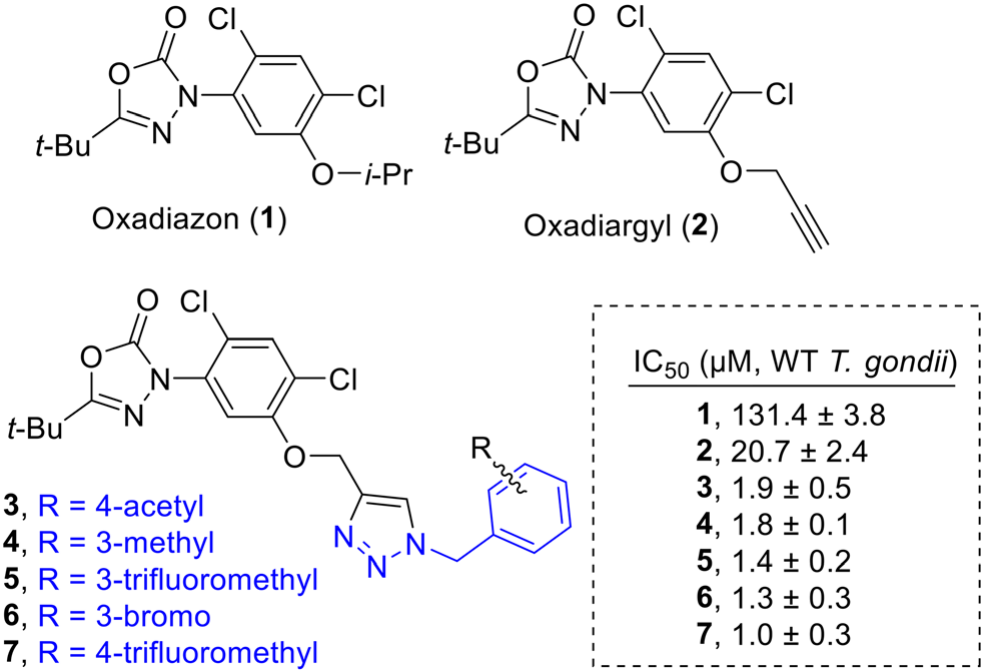
Herbicide PPO inhibitor oxadiazon (1) and derivatives (2–7) exhibit potent growth inhibition against *Toxoplasma gondii*.

In this study, we synthesized a library of 23 oxadiazon derivatives (11a–11w, Fig. 2) containing a triazole moiety bearing aliphatic chains functionalized with diverse groups, including hydroxyl, halogen, nitrile, α-olefin, epoxide, pivalate, sulfonate, phosphonate, amino alcohol, and halohydrin. The synthesis began with the preparation of functionalized aliphatic azide intermediates, which were obtained by reacting alkyl bromides with sodium azide in DMF. In some cases, subsequent functional group interconversions afforded the desired azides in good to excellent yields (S9a–f and S10g–w; see SI for details). These azides were then subjected to copper-catalyzed Huisgen 1,3-dipolar cycloaddition with commercially available oxadiargyl (2), resulting in the target oxadiazon derivatives in moderate to excellent yields (11a–w; see SI for details). Notably, the iso-pentyl (11a) and heptyl (11b) derivatives were isolated in yields of 90% and 95%, respectively. Similarly, derivatives bearing α-olefin (11c), butylbenzene (11d), butoxymethylbenzene (11e), and hydroxyl (11f) groups were obtained in 90–96% yield. The sulfonate (11g) and pivalate (11h) derivatives were isolated in 80% and 90% yield, respectively, while halogenated derivatives (11i, 90%; 11j, 90%) as well as nitrile (11k, 98%) and epoxide (11l, 92%) derivatives were successfully synthesized. Additional analogs included halohydrin derivatives (11m, 78%; 11n, 82%) and a series of amino alcohol derivatives (11o–r) isolated in 86–92% yield. Finally, phosphonate derivatives (11s–u) were obtained in 85–90% yield, while the corresponding phosphonic acid derivatives 11v and 11w were isolated in 68% and 74% yields, respectively.

**Fig. 2:**
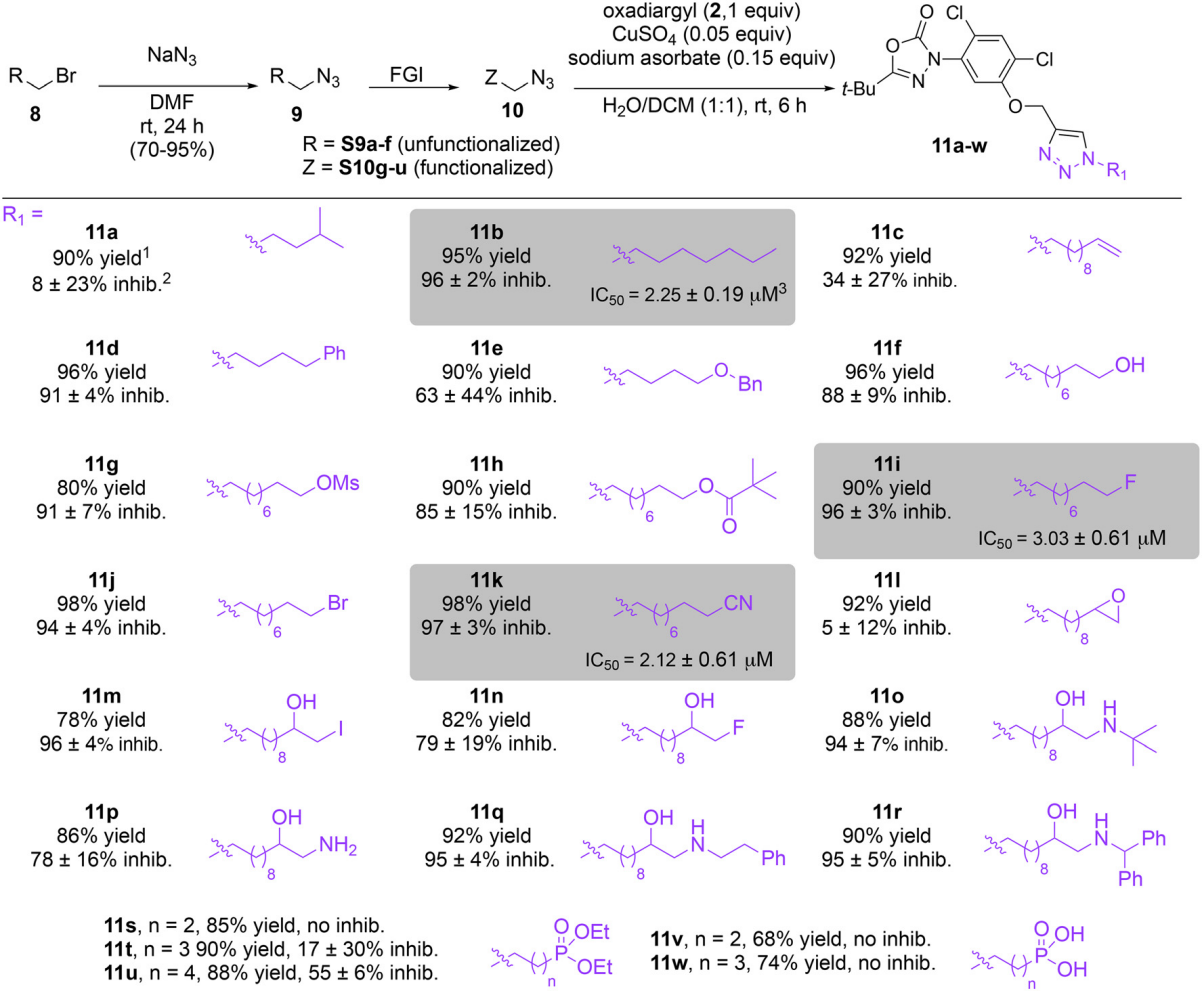
Triazole-oxadiargyl library evaluated against WT *T. gondii*.^1^ Isolated yields.^2^ % growth inhibition of WT *T. gondii* at 5 μM (mean ± STD for three biological replicates).^3^ Structures highlighted in grey background were the most potent, and IC_50_ values were solved (mean ± STD for three biological replicates, two technical replicates each).

Oxadiazon derivatives 11a–w were assessed for their ability to inhibit the intracellular growth of wild-type *T. gondii* expressing a luciferase reporter at 5 μM concentration, using our previously developed assay.^16,17,19^ The average percent inhibition over three biological replicates was calculated, and six compounds exhibited ≥95% growth inhibition (*i.e.* compounds 11b, 11i, 11k, 11m, 11q, and 11r). IC_50_ values were calculated for the three most potent analogs, 11b (2.25 ± 0.19 μM), 11i (3.03 ± 0.61 μM), and 11k (2.12 ± 0.61 μM) (Fig. 2, shaded boxes). We next elected to move forward with additional evaluations of 11k owing to its strong potency and more favorable solubility profile.

First, the IC_50_ of 11k against WT *T. gondii* was compared to PPO-knockout and complemented strains bearing the luciferase reporter (Table 1).^16,19^ Consistent with our previous findings,^19^ oxadiazon analog 11k primarily inhibits intracellular growth of *T. gondii* by targeting PPO, thereby disrupting heme biosynthesis. Compound 11k was approximately 9.5 times less potent against the knockout mutant, reinforcing that PPO inhibition is its primary mechanism of action (Table 1, column 3). Furthermore, its potency was restored against a strain complemented with *Toxoplasma* PPO (*ΔppoPPO::NLuc*), further supporting PPO disruption as the primary mode of inhibition (Table 1, column 4). Additionally, 11k was evaluated for cytotoxicity against human foreskin fibroblast (HFF) cells using the alamarBlue viability assay and only exhibited slight cytotoxicity at 100 μM.

**Table 1:** Evaluation of selected oxadiazon derivatives against wild-type, knockout, and complemented strains of *Toxoplasma gondii*

Compound	WT*::Nluc*^a^ (μM)	*Δppo::Nluc* (μM)	*ΔppoPPO::Nluc* (μM)
11k	2.12 ± 0.61	20.24 ± 11.3	1.21 ± 0.63

a IC_50_ values for intracellular growth inhibition (mean ± STD for three biological replicates, two technical replicates each).

Our previous work demonstrated that deletion of TgPPO disrupted heme biosynthesis by ∼50% and impaired the parasite lytic cycle.^16^ Based on this, we hypothesized that 11k treatment would inhibit parasite growth by targeting TgPPO and reducing heme production. To test this, parasites were cultured in the presence of 11k at 1× IC_50_ (2.1 μM) or 3× IC_50_ (6.3 μM), and proliferation was quantified using a luciferase-based assay reported before.^16^ After 96 h of treatment, parasite growth was reduced by 57% and 90% at 1× IC_50_ and 3× IC_50_, respectively (Fig. 3A), relative to the vehicle control (*i.e.* DMSO-treated parasites). We next assessed whether the treatment of 11k decreases heme abundance in the parasites. Parasites were cultured with 11k at 1× IC_50_ and 3× IC_50_ for 4 days, with fresh inhibitor added daily, and intracellular heme levels were quantified using a fluorescence-based assay.^20^ Heme content was decreased by 22% and 48% at 1× IC_50_ and 3× IC_50_, respectively (Fig. 3B) compared to the DMSO-treated control, confirming that 11k was able to penetrate the parasite's membrane and perturbed the heme biosynthetic pathway in the parasites.

**Fig. 3:**
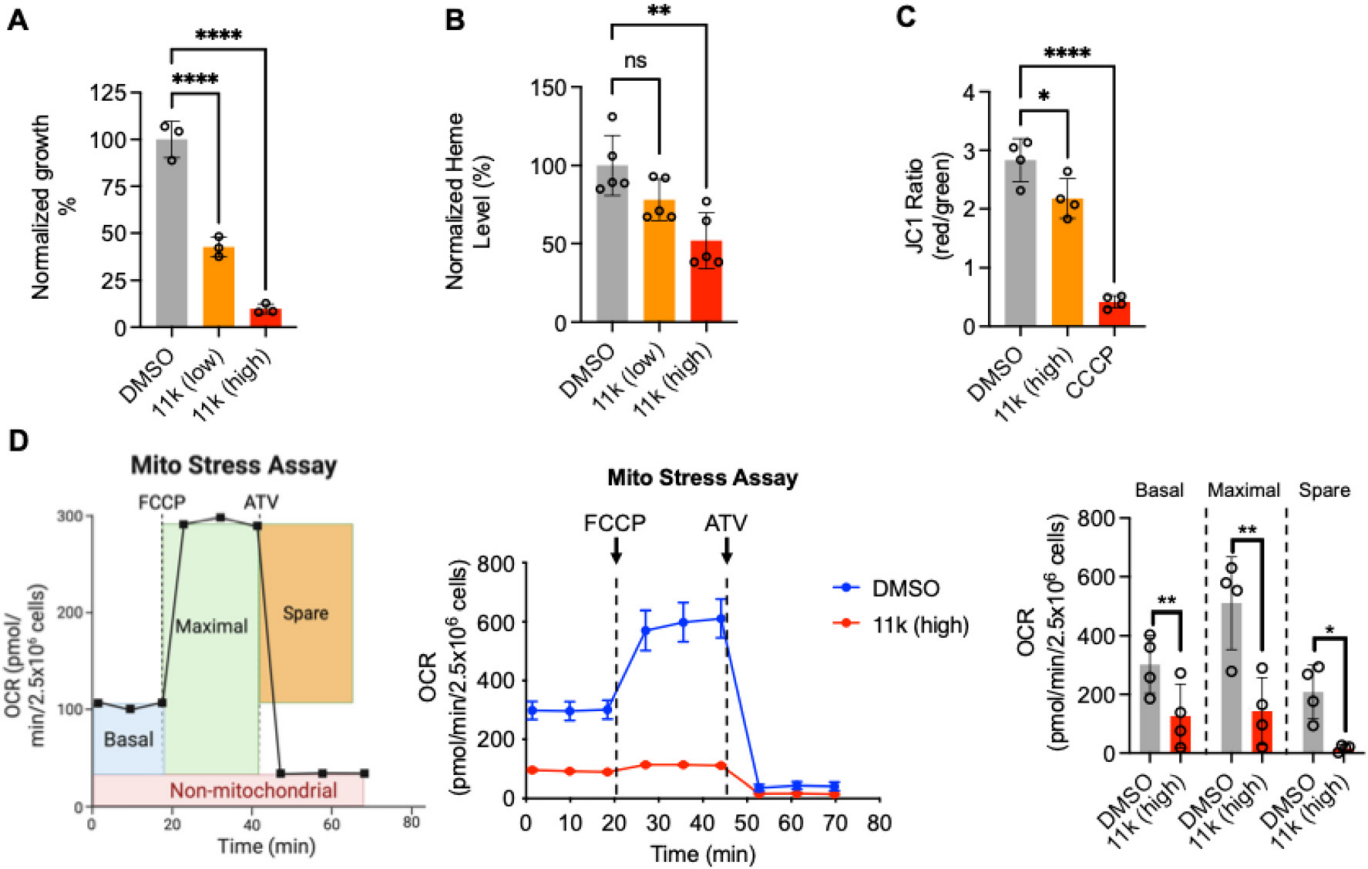
Compound 11k treatment significantly inhibited parasite growth, heme production, and mitochondrial function in *Toxoplasma gondii*. (A) Parasite growth was severely reduced at 1× IC_50_ and 3× IC_50_ concentrations of 11k, as determined by a luciferase-based growth assay. (B) Heme levels were decreased in *Toxoplasma* parasites following 11k treatment, measured by a fluorescence-based heme assay. (C) Mitochondrial membrane potential was reduced in 11k-treated parasites. Parasites treated with CCCP served as a positive control. (D) Schematic representation of the modified Seahorse mitochondrial stress test, indicating the bioenergetic parameters measured (*i.e.* basal, maximal, and spare mitochondrial OCRs). A representative stress assay is shown, and average values from four biological replicates were plotted for statistical comparison. Statistical significance in panels (A to C) was determined by one-way ANOVA, and bioenergetic results in panel (D) were analyzed using paired *t*-tests. n.s., not significant; *, *p* < 0.05; **, *p* < 0.01; ****, *p* < 0.0001. Abbreviations: CCCP, carbonyl cyanide *m*-chlorophenyl hydrazone; FCCP, carbonyl cyanide-*p*-trifluoromethoxyphenylhydrazone; ATV, atovaquone.

Since 11k treatment at 6.3 μM reduced heme abundance by ∼50%, similar to the inhibition observed in the TgPPO knockout,^16^ we next investigated its impact on mitochondrial physiology at 3× IC_50_. First, mitochondrial membrane potential was evaluated using the JC-1 assay, in which healthy mitochondria promote red J-aggregate formation, whereas depolarized mitochondria emit green fluorescence. Treatment with 11k decreased the red/green ratio by 23% compared to DMSO-treated parasites (Fig. 3C), indicating that mitochondrial membrane potentials are reduced due to lack of heme production, which further impairs the function of the mitochondrion by reducing hemoprotein translocation. To further characterize mitochondrial function, we performed a modified mitochondrial stress test using Seahorse analysis (Fig. 3D). Since *T. gondii* lacks ETC complex I and possesses a unique complex III,^21,22^ atovaquone (ATQ) was used to fully block respiration. Parasites treated with 11k showed a 58% reduction in basal respiration, while maximal and spare respiration rates were decreased by 72% and 92%, respectively (Fig. 3D). These data suggest that reduced heme production in the parasites impaired electron transport chain (ETC) activity, likely through decreased availability of hemoproteins such as cytochromes.

In conclusion, we have developed a library of oxadiazon derivatives bearing functionalized alkyl groups appended to the scaffold by means of a straightforward click chemistry approach. Six of the compounds exhibited ≥95% intracellular growth inhibition of WT *T. gondii* at 5 μM concentration. The three most potent compounds, 11b, 11i, and 11k, exhibited IC_50_ values ranging between 2.25 and 3.03 μM. Evaluation of 11k against PPO-knockout and complemented strains of *T. gondii* confirmed that PPO disruption is the main pathway for responsible for growth inhibition. Our biological findings demonstrate that the oxadiazon derivative 11k inhibits TgPPO, disrupts heme biosynthesis, and severely compromises mitochondrial activity in *Toxoplasma* parasites.

## Methods

### General methods

#### Chemistry

All reagents were purchased from commercial sources and used without purification unless otherwise noted. All non-aqueous reactions were performed under an inert atmosphere of nitrogen in flame-dried glassware containing a stir bar. Acetonitrile (ACN), tetrahydrofuran (THF), dichloromethane (DCM), methanol (MeOH), dimethylformamide (DMF) and pyridine (py) were obtained from commercial sources and dried following standard distillation procedures. All other solvents were obtained from commercial sources and used without drying unless otherwise noted. All water and aqueous solutions were made using deionized (DI) water. Flash column chromatography was carried out using ZEOCHEM silica gel (40–63 μm). Analytical and preparative thin-layer chromatography (TLC) were performed on Sorbtech silica gel TLC plates. ^1^H and ^13^C NMR including 2D NMR spectra were obtained using Bruker avance 300 and 500 MHz spectrometers. Chemical shifts are reported in parts per million (ppm). Spectra are referenced to residual solvent peaks. The following abbreviations were used to designate multiplicities: s = singlet, d = doublet, t = triplet, q = quartet, p = pentet, sx = sextet, sep = septet, m = multiplet, br = broad. Infrared spectroscopy data were collected using an IR Affinity-1S instrument (with MIRacle 10 single reflection ATR accessory), and peaks are described as strong (s), moderate (m), and weak (w). All known compounds were characterized by ^1^H and ^13^C NMR and are in complete agreement with samples reported elsewhere. All new compounds were characterized by ^1^H, ^13^C and 2D NMR, ATR-FTIR, HRMS, and melting point (where appropriate). HRMS data were collected using an instrument equipped with electrospray ionization in positive mode (ESI^+^) and a time-of-flight (TOF) detector.

### Mammalian cell and parasite culture

*Toxoplasma gondii* parasites were passaged in human foreskin fibroblasts (HFFs, ATCC, SCRC-1041), which were grown in Dulbecco's modified Eagle's medium (DMEM) supplemented with 10% cosmic calf serum (D10 medium), 10 mM HEPES, 4 mM glutamine, 100 U penicillin/streptomycin at 37 °C with 5% CO_2_.

### Evaluation of inhibition potency of oxidiargyl–triazole derivatives using the bioluminescence-based growth assay

The initial screening of synthesized oxadiazon derivatives was performed at 5 μM. Freshly lysed parasites were harvested by membrane filtration, as previously described.^17^ A total of 1500 RHΔ*ku80*Δ*hxg::NLuc* parasites (referred to as WT::*NLuc* hereafter) were inoculated into each well of 96-well white plates. Parasites were allowed to invade host cells for 4 h, after which non-invaded parasites were washed away, and the medium was replaced with fresh D10 supplemented with 5 μM oxadiazon derivatives. Wells containing D10 medium with DMSO served as vehicle controls for normalization of parasite growth across treatments. Parasites were allowed to grow for 96 h, and bioluminescence was determined as previously described.^17^ Growth inhibition was calculated using the following equation: ((mean readings of bioluminescence from the wells incubated with DMSO − mean readings of bioluminescence from the wells incubated with oxadiazon derivatives)/mean reading of bioluminescence from the wells incubated with DMSO) × 100%. Compounds were then ranked from highest to lowest inhibition.

The top three derivatives were further evaluated to determine their half-maximal inhibitory concentrations (IC_50_). Following the same procedures, 1500 WT::*NLuc*, 1500 *ΔppoPPO::NLuc*, or 7500 *Δppo::NLuc* parasites were inoculated into 96-well plates. The maximum concentration of each compound was 100 μM, followed by three-fold serial dilutions across 10 concentrations. Wells containing DMSO served as vehicle controls. Normalized bioluminescence readings were plotted in Prism version 10 and fitted using the [inhibitor] *vs.* normalized response function. All assays were performed in three independent biological replicates, each with two technical replicates. Calculated IC_50_ values were averaged, and the standard deviations were reported.

### Toxicity quantification of oxidiargyl triazole derivatives in HFFs using the alamarBlue assay

Human foreskin fibroblasts (HFFs) were seeded into 96-well clear plates and grown to confluent monolayers. Compound 11k was prepared in D10 medium at an initial concentration of 100 μM and subsequently serially diluted in 3-fold increments to obtain 11 final concentrations, and the cells were incubated for 96 h at 37 °C with 5% CO_2_. Following treatment, the medium was replaced with D10 containing 0.004% (m/v) resazurin and incubated for 4 h under the same conditions. Absorbance was then measured at 570 and 600 nm using a BioTek H1 hybrid plate reader. Cell viability was calculated according to previously reported methods.^19^

### Heme quantification in *Toxoplasma* parasites

*Toxoplasma* parasites were cultured with or without compound 11k at concentrations corresponding to the 1× IC_50_ or 3× IC_50_ for 48 h prior to purification. Culture medium was replenished every 24 h with freshly supplemented compound. Parasites were harvested by syringe passage, filter purification, and resuspension in ice-cold PBS, followed by centrifugation at 1000 × *g* for 10 min at 4 °C. The pellet was washed twice with PBS, each time centrifuged at 1000 × *g* for 10 min at 4 °C. The final pellet was counted using a hemocytometer, centrifuged at 5000 × *g* for 5 min at 4 °C, resuspended in 400 μL of ice-cold PBS, and subjected to sonication.

Parasite lysates were sonicated three times using a Branson Analog Sonifier S-250A equipped with a tapered 1/8 inch microtip (output intensity = 3, duty cycle = 20%), with 30-sec intervals between pulses to prevent overheating. Heme standards were prepared in parallel to calculate heme content per parasite. For measurement, 100 μL of parasite lysate or heme standard was mixed with 900 μL of 2 M aq. oxalic acid and vortexed. One set of samples was boiled for 30 min, while the other was kept at room temperature to serve as a background control. Two hundred microliters of each oxalic acid/sample mixture were transferred in triplicate into black 96-well plates and measured using a BioTek Synergy H1 hybrid multi-mode microplate reader under the following settings: excitation = 400 nm, emission = 608 nm, optics = top, gain = 135, read speed = normal, delay = 100 msec, measurements/data point = 10, and read height = 7 mm. The assay was performed in at least three independent biological replicates for statistical comparison.

### Mitochondrial membrane potential quantification

To assess the health status of drug-treated parasites, a JC-1 assay was used to quantify mitochondrial membrane potential. 11k-treated parasites were filter-purified, centrifuged, washed, and resuspended in PBS at 5 × 10^8^ tachyzoites per mL. Parasite suspensions were incubated at 37 °C with 2 μM JC-1 dye for 15 min. Wild-type parasites treated with CCCP (carbonyl cyanide *m*-chlorophenylhydrazone) served as positive controls. Following staining, parasites were washed once with warm PBS to remove residual dye and resuspended at 5 × 10^8^ tachyzoites per mL. Aliquots of 100 μL were loaded into black 96-well plates in triplicate, and fluorescence was measured at excitation 488 nm with emission recorded at 530 nm (green) and 585 nm (red). The ratio of red (585 nm) to green (530 nm) fluorescence was reported as an indicator of mitochondrial membrane potential. The assay was performed in four independent biological replicates.

### Seahorse mitochondrial stress assay

To evaluate the bioenergetic parameters of the parasite's mitochondria, we performed an Agilent Seahorse mitochondrial stress test to measure basal, maximal, and spare mitochondrial oxygen consumption rates (mOCRs). Because *Toxoplasma* mitochondria lack ETC complex I and possess a unique complex III, rendering them insensitive to rotenone and antimycin A,^21^ atovaquone (ATQ) was used to completely inhibit mitochondrial respiration. Following the manufacturer's instructions, freshly lysed, filter-purified parasites were seeded at 2.5 × 10^6^ tachyzoites per well on CellTak-coated 24-well Seahorse plates (Agilent). Parasites were incubated in DMEM medium (Agilent) supplemented with 10 mM glucose and 4 mM l-glutamine for basal OCR measurements. FCCP (2 μM) and ATQ (1 μM) were sequentially injected to induce maximal respiration and then fully block mitochondrial respiration, respectively. Oxygen consumption rates were recorded three times following each injection. Energetic parameters were calculated as previously described.^22^ The assay was repeated in four biological replicates.

### General procedure for synthesis of oxadiargyl–triazole derivatives 11a–w

In a flame-dried 20 mL round-bottom flask equipped with a magnetic stir bar, oxadiargyl (0.2 mmol) was dissolved in a 1 : 1 mixture of DCM and water (2 mL). The appropriate azide (0.24 mmol) was then added, followed by anhydrous CuSO_4_ (0.01 mmol) and sodium ascorbate (0.03 mmol). The reaction mixture was stirred vigorously at room temperature for 6 h. Upon completion, the mixture was dried and purified by flash chromatography on silica gel, using gradient elution from 100% hexanes to 80% hexanes/ethyl acetate. This process afforded the corresponding oxadiargyl derivative as an off-white oil or solid in 65–98% isolated yield.

### Analytical data for oxadiargyl–triazole derivatives 11a–w

#### 5-(*tert*-Butyl)-3-(2,4-dichloro-5-((1-isopentyl-1*H*-1,2,3-triazol-4-yl)methoxy)phenyl)-1,3,4-oxadiazol-2(3*H*)-one

Off-white viscous oil (90% yield); IR: (neat) *ν* (cm^−1^): 2984, 1761, 1623, 1421, 1415, 1316, 1274, 1124, 1108, 1075, 926, 921, 756, 536; ^1^H NMR (500 MHz, CDCl_3_) *δ* 7.68 (s, 1H), 7.54 (s, 1H), 7.26 (s, 1H), 5.32 (s, 2H), 4.44–4.35 (m, 2H), 1.89–1.79 (m, 2H), 1.61 (s, 1H), 1.39 (s, 9H), 0.98 (d, *J* = 6.7 Hz, 6H); ^13^C{^1^H} NMR (126 MHz, CDCl_3_) *δ* 163.6, 153.0, 152.1, 142.7, 131.4, 125.2, 123.9, 122.8, 113.8, 63.8, 48.9, 38.9, 33.0, 27.0, 25.6, 22.2; HRMS (ESI-TOF) *m*/*z*: [M + H]^+^ calcd for C_20_H_26_Cl_2_N_5_O_3_ 454.1413; found 454.1415.
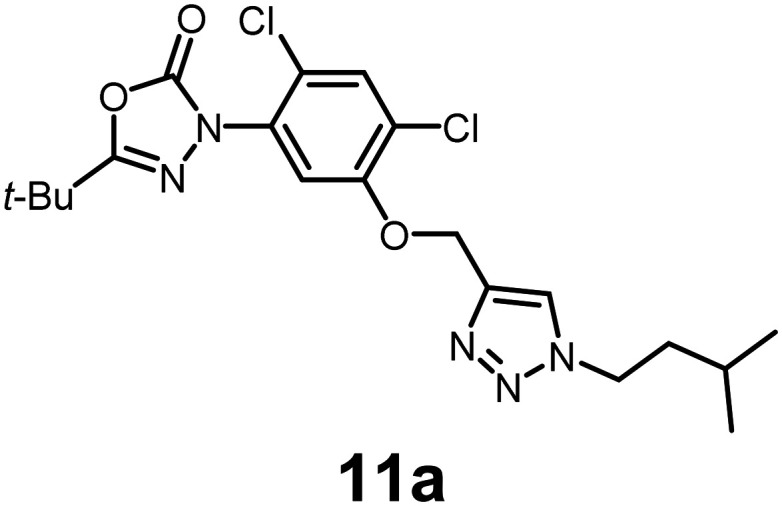


#### 5-(*tert*-Butyl)-3-(2,4-dichloro-5-((1-heptyl-1*H*-1,2,3-triazol-4-yl)methoxy)phenyl)-1,3,4-oxadiazol-2(3*H*)-one

Off-white viscous oil (95% yield); IR: (neat) *ν* (cm^−1^): 3045, 1778, 1643, 1484, 1424, 1346, 1228, 1152, 1147, 1022, 958, 841, 648; ^1^H NMR Off-white viscous oil (500 MHz, CDCl_3_) *δ* 7.68 (s, 1H), 7.53 (s, 1H), 7.26 (s, 1H), 5.31 (s, 2H), 4.36 (t, *J* = 7.2 Hz, 2H), 1.92 (m, 4H), 1.39 (s, 9H), 1.33–1.29 (m, 6H), 0.88 (t, *J* = 7.4 Hz, 3H); ^13^C{^1^H} NMR (126 MHz, CDCl_3_) *δ* 163.6, 152.9, 152.1, 142.6, 131.4, 125.1, 123.9, 122.9, 113.7, 63.7, 50.6, 33.0, 31.6, 30.21, 28.6, 27.0, 26.4, 22.5, 14.0; HRMS (ESI-TOF) *m*/*z*: [M + H]^+^ calcd for C_22_H_30_Cl_2_N_5_O_3_ 482.1726; found 482.1728.
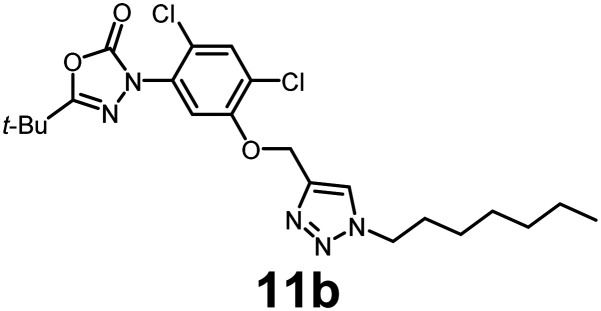


#### 5-(*tert*-Butyl)-3-(2,4-dichloro-5-((1-(undec-10-en-1-yl)-1*H*-1,2,3-triazol-4-yl)methoxy)phenyl)-1,3,4-oxadiazol-2(3*H*)-one

Off-white viscous oil (92% yield); IR: (neat) *ν* (cm^−1^): 3120, 2984, 1756, 1669, 1651, 1531, 1454, 1345, 1228, 1174, 1012, 1054, 893, 824, 623; ^1^H NMR (500 MHz, CDCl_3_) *δ* 7.46 (s, 1H), 7.22 (s, 1H), 5.73 (dt, *J* = 16.4, 8.0 Hz, 1H), 5.22 (s, 1H), 4.88 (dd, *J* = 31.3, 13.8 Hz, 2H), 4.81–4.76 (m, 1H), 4.29 (t, *J* = 7.5 Hz, 1H), 3.18 (m, 3H), 1.96 (q, *J* = 7.3, 6.7 Hz, 2H), 1.85 (m, 2H), 1.31 (s, 9H), 1.23 (m, 10H); ^13^C{^1^H} NMR (126 MHz, CDCl_3_) *δ* 163.6, 152.9, 152.1, 139.0, 131.3, 131.3, 125.1, 123.8, 114.1, 113.8, 63.5, 50.5, 50.1, 33.7, 30.1, 29.2, 29.0, 28.9, 28.8, 26.9, 26.4; HRMS (ESI-TOF) *m*/*z*: [M + H]^+^ calcd for C_26_H_36_Cl_2_N_5_O_3_ 536.2195; found 536.2196.
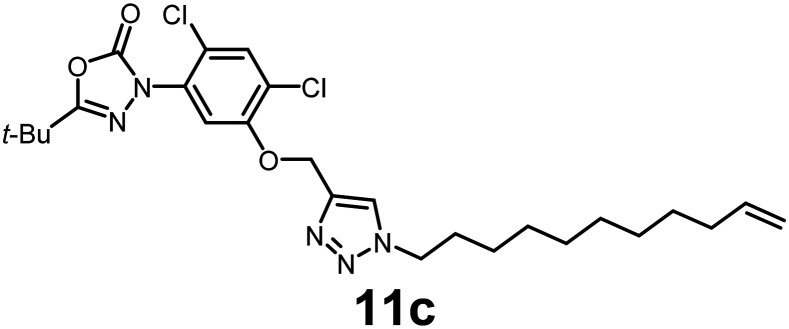


#### 5-(*tert*-Butyl)-3-(2,4-dichloro-5-((1-(4-phenylbutyl)-1*H*-1,2,3-triazol-4-yl)methoxy)phenyl)-1,3,4-oxadiazol-2(3*H*)-one

Off-white viscous oil (96% yield); IR: (neat) *ν* (cm^−1^): 3103, 1754, 1623, 1645, 1562, 1482, 1323, 1217, 1123, 1041, 923, 886, 628; ^1^H NMR (500 MHz, CDCl_3_) *δ* 7.65 (s, 1H), 7.54 (s, 1H), 7.32–7.28 (m, 3H), 7.27 (s, 1H), 7.21–7.14 (m, 3H), 5.31 (s,2H), 4.39 (s, 2H), 2.68 (m, 3H), 1.96 (dd, *J* = 6.2, 2.9 Hz, 1H), 1.69–1.66 (m, 1H), 1.40 (s, 9H); ^13^C{^1^H} NMR (126 MHz, CDCl_3_) *δ* 163.6, 152.9, 152.1, 142.7, 141.4, 131.4, 128.5, 128.4, 126.1, 125.1, 123.9, 123.0, 113.8, 63.7, 50.4, 35.1, 29.7, 28.1, 27.0; HRMS (ESI-TOF) *m*/*z*: [M + H]^+^ calcd for C_25_H_28_Cl_2_N_5_O_3_ 516.1569; found 516.1568.
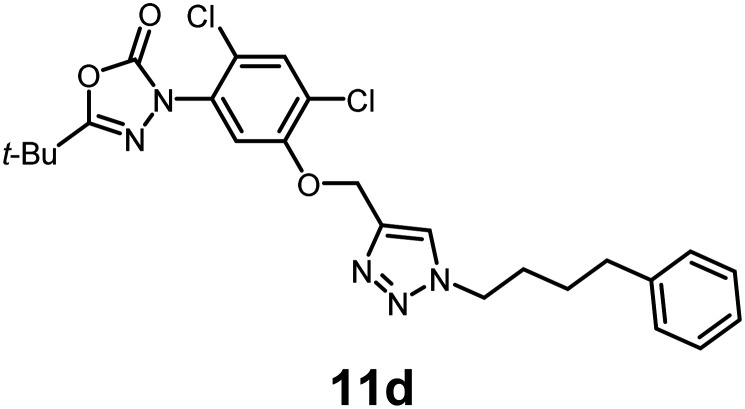


#### 3-(5-((1-(4-(Benzyloxy)butyl)-1*H*-1,2,3-triazol-4-yl)methoxy)-2,4-dichlorophenyl)-5-(*tert*-butyl)-1,3,4-oxadiazol-2(3*H*)-one

Pale-yellow viscous oil in 90% yield; IR: (neat) *ν* (cm^−1^): 2928, 1762, 1661, 1538, 1469, 1438, 1314, 1286, 1118, 1056, 928, 763, 641, 556; ^1^H NMR (500 MHz, CDCl_3_) *δ* 7.74–7.66 (s, 1H), 7.63 (s, 1H), 7.50 (s, 1H), 7.35–7.25 (m, 5H), 5.53 (d, *J* = 6.4 Hz, 2H), 5.29–5.22 (m, 2H), 4.43–4.35 (m, 2H), 3.54–3.47 (m, 2H), 2.04 (dt, *J* = 14.9, 8.9 Hz, 2H), 1.63 (m, 2H), 1.37 (s, 9H); ^13^C{^1^H} NMR (126 MHz, CDCl_3_) *δ* 163.6, 153.0, 152.9, 152.18, 152.06, 143.0, 142.5, 138.3, 134.4, 131.43, 131.37, 129.1, 128.8, 128.4, 128.1, 127.7, 125.11, 125.09, 123.9, 123.8, 123.19, 123.16, 114.0, 113.8, 73.0, 69.3, 63.7, 54.3, 50.3, 33.0, 27.4, 27.0, 26.6; HRMS (ESI-TOF) *m*/*z*: [M + H]^+^ calcd for C_26_H_30_Cl_2_N_5_O_4_ 546.1675; found 546.1678.
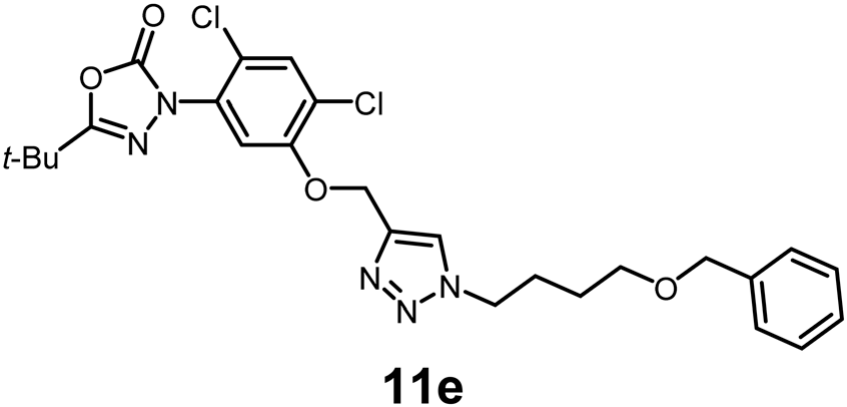


#### 5-(*tert*-Butyl)-3-(2,4-dichloro-5-((1-(9-hydroxynonyl)-1*H*-1,2,3-triazol-4-yl)methoxy)phenyl)-1,3,4-oxadiazol-2(3*H*)-one

Off-white viscous oil (96% yield); IR: (neat) *ν* (cm^−1^): 3531, 2935, 1762, 1623, 1523, 1442, 1345, 1231, 1125, 1054, 1023, 956, 862, 674, 586; ^1^H NMR (500 MHz, CDCl_3_) *δ* 7.68 (s, 1H), 7.52 (s, 1H), 7.27 (s, 1H), 4.35 (t, *J* = 7.3 Hz, 2H), 4.11 (q, *J* = 7.1 Hz, 1H), 3.62 (t, *J* = 6.6 Hz, 2H), 2.04 (s, 1H), 1.92 (m, 3H), 1.69 (m, 1H), 1.54 (dq, *J* = 8.0, 6.5 Hz, 2H), 1.37 (s, 9H), 1.32–1.28 (m, 10H); ^13^C{^1^H} NMR (126 MHz, CDCl_3_) *δ* 163.6, 152.9, 152.1, 142.6, 131.4, 125.1, 123.9, 122.9, 113.7, 63.7, 62.9, 50.5, 32.7, 30.2, 29.3, 29.2, 28.8, 27.0, 26.4, 25.6; HRMS (ESI-TOF) *m*/*z*: [M + H]^+^ calcd for C_24_H_34_Cl_2_N_5_O_4_ 526.1988; found 526.1986.
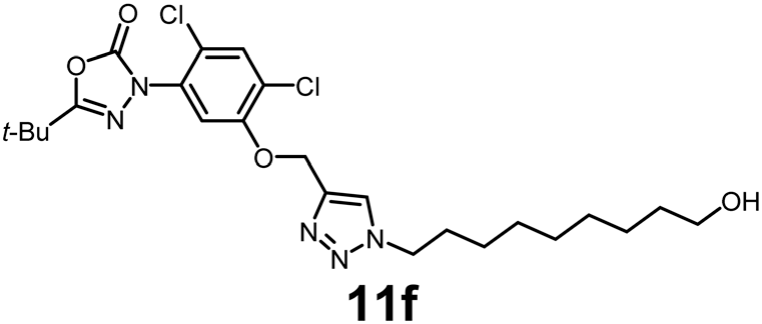


#### 9-(4-((5-(5-(*tert*-Butyl)-2-oxo-1,3,4-oxadiazol-3(2*H*)-yl)-2,4-dichlorophenoxy)methyl)-1*H*-1,2,3-triazol-1-yl)nonyl methanesulfonate

Off-white viscous oil (80% yield); IR: (neat) *ν* (cm^−1^): 2995, 1763, 1652, 1609, 1595, 1434, 1328, 1263, 1131, 1047, 968, 847, 723, 668, 547; ^1^H NMR (500 MHz, CDCl_3_) *δ* 7.68 (s, 1H), 7.60–7.47 (s, 1H), 7.25 (s, 1H), 5.28 (s, 2H), 4.65–4.58 (m, 1H), 4.32 (t, *J* = 7.4 Hz, 2H), 4.18 (q, *J* = 6.5, 5.7 Hz, 1H), 3.53–3.47 (m, 1H), 3.04–2.94 (m, 2H), 2.34 (s, 1H), 2.02 (q, *J* = 7.6, 7.2 Hz, 1H), 1.91–1.84 (m, 2H), 1.76–1.64 (m, 2H), 1.38 (s, 9H), 1.28 (m, 8H); ^13^C{^1^H} NMR (126 MHz, CDCl_3_) *δ* 152.9, 152.1, 142.5, 131.4, 131.3, 125.0, 123.8, 123.1, 113.8, 70.2, 63.6, 50.5, 37.3, 32.9, 30.13, 30.11, 29.12, 29.05, 29.0, 28.88, 28.75, 28.72, 28.67, 28.65, 26.98, 26.36, 26.31, 25.3; HRMS (ESI-TOF) *m*/*z*: [M + H]^+^ calcd for C_25_H_36_Cl_2_N_5_O_6_S 604.1763; found 604.1765.
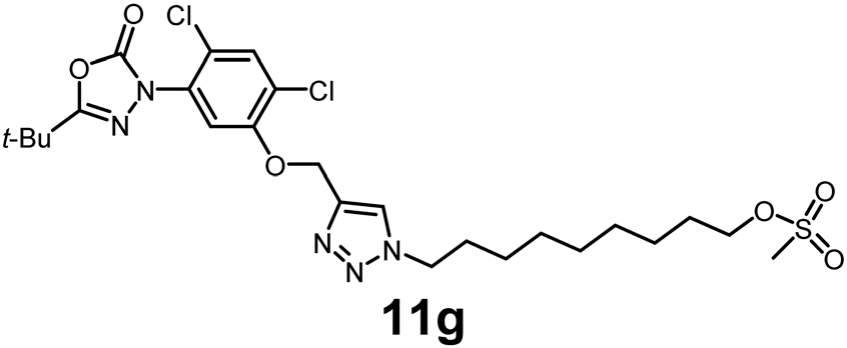


#### 9-(4-((5-(5-(*tert*-Butyl)-2-oxo-1,3,4-oxadiazol-3(2*H*)-yl)-2,4-dichlorophenoxy)methyl)-1*H*-1,2,3-triazol-1-yl)nonyl pivalate

Off-white viscous oil (90% yield); IR: (neat) *ν* (cm^−1^): 3021, 1785, 1728, 1625, 1523, 1485, 1323, 1218, 1173, 1038, 964, 856, 769, 628, 527; ^1^H NMR (500 MHz, CDCl_3_) *δ* 7.65 (s, 1H), 7.41 (s, 1H), 7.19 (s, 1H), 5.18 (s, 2H), 4.25 (m, 3H), 3.93 (m, 2H), 3.48 (m, 2H), 3.17 (m, 3H), 1.81 (m, 1H), 1.45 (m, 4H), 1.28 (s, 9H), 1.20 (m, 11H); ^13^C{^1^H} NMR (126 MHz, CDCl_3_) *δ* 178.5, 163.4, 152.9, 152.0, 142.4, 131.4, 131.2, 124.9, 123.6, 123.2, 113.7, 64.3, 63.5, 62.44, 62.40, 51.3, 50.4, 32.6, 29.34, 29.2, 29.0, 28.74, 28.72, 27.1, 26.9, 26.6, 25.7; HRMS (ESI-TOF) *m*/*z*: [M + H]^+^ calcd for C_29_H_42_Cl_2_N_5_O_5_ 610.2563; found 610.2565.
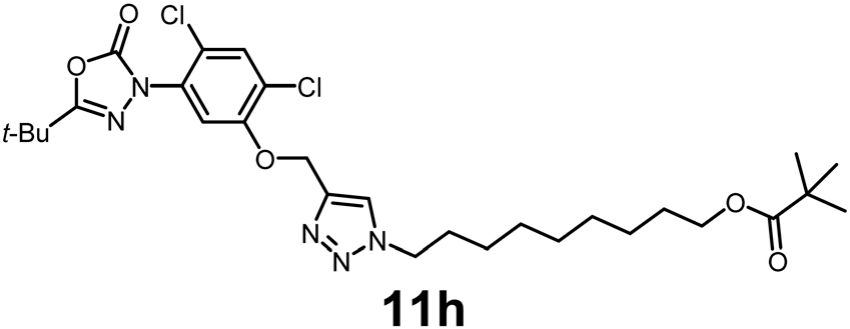


#### 5-(*tert*-Butyl)-3-(2,4-dichloro-5-((1-(9-fluorononyl)-1*H*-1,2,3-triazol-4-yl)methoxy)phenyl)-1,3,4-oxadiazol-2(3*H*)-one

Off-white viscous oil (90% yield); IR: (neat) *ν* (cm^−1^): 2892, 1732, 1661, 1645, 1512, 1462, 1317, 1283, 1145, 1032, 1007, 957, 876, 752, 589; ^1^H NMR (500 MHz, CDCl_3_) *δ* 7.68 (s, 1H), 7.53 (s, 1H), 7.26 (s, 1H), 5.31 (s, 2H), 4.39–4.35 (m, 2H), 3.53 (t, *J* = 6.7 Hz, 1H), 1.92 (p, *J* = 7.6 Hz, 2H), 1.80–1.69 (m, 1H), 1.72–1.59 (m, 1H), 1.42 (d, *J* = 6.7 Hz, 1H), 1.40 (s, 0H), 1.38 (s, 9H), 1.32 (dt, *J* = 16.6, 8.4 Hz, 10H); ^13^C{^1^H} NMR (126 MHz, CDCl_3_) *δ* 163.6, 152.9, 152.1, 142.6, 131.4, 125.1, 123.9, 122.9, 113.7, 84.2 (d, *J*_*C-F*_ = 164.0 Hz), 63.7, 50.5, 45.1, 33.0, 32.5, 30.2, 29.21, 29.17, 29.15, 28.8, 28.7, 27.0, 26.8, 26.4; ^19^F NMR (471 MHz, CDCl_3_) *δ* −218.07; HRMS (ESI-TOF) *m*/*z*: [M + H]^+^ calcd for C_24_H_33_Cl_2_FN_5_O_3_ 528.1944; found 528.1946.
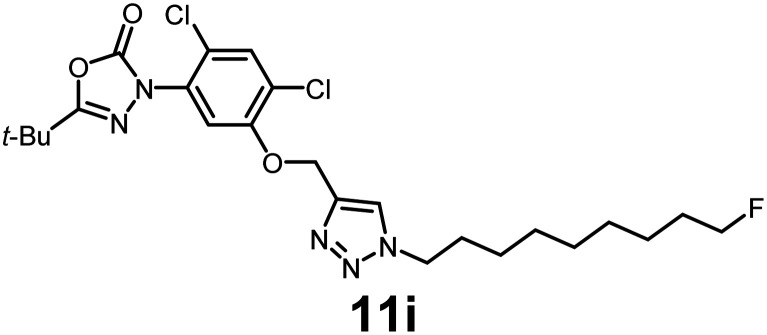


#### 3-(5-((1-(9-Bromononyl)-1*H*-1,2,3-triazol-4-yl)methoxy)-2,4-dichlorophenyl)-5-(*tert*-butyl)-1,3,4-oxadiazol-2(3*H*)-one

Pale-yellow viscous oil in 98% yield; IR: (neat) *ν* (cm^−1^): 2928, 1763, 1612, 1541, 1438, 1341, 1274, 1132, 1074, 941, 832, 686, 528, 567; ^1^H NMR (500 MHz, CDCl_3_) *δ* 7.75–7.64 (s, 1H), 7.56–7.48 (s, 1H), 7.32–7.23 (s, 1H), 5.35–5.25 (m, 2H), 4.33 (t, *J* = 7.5 Hz, 2H), 3.50 (m, 1H), 3.37 (m, 1H), 1.89 (m, 2H), 1.86–1.76 (m, 1H), 1.77–1.68 (m, 1H), 1.39 (m, 2H), 1.36 (s, 9H), 1.33–1.23 (m, 8H); ^13^C{^1^H} NMR (126 MHz, CDCl_3_) *δ* 163.7, 152.9, 152.1, 142.5, 131.42, 131.36, 125.1, 123.8, 123.0, 113.8, 63.7, 50.5, 45.1, 34.0, 32.9, 32.7, 32.5, 30.2, 29.14, 29.11, 28.8, 28.7, 28.5, 28.0, 27.02, 26.99, 26.7, 26.4; HRMS (ESI-TOF) *m*/*z*: [M + H]^+^ calcd for C_24_H_33_BrCl_2_N_5_O_3_ 588.1144; found 588.1145.
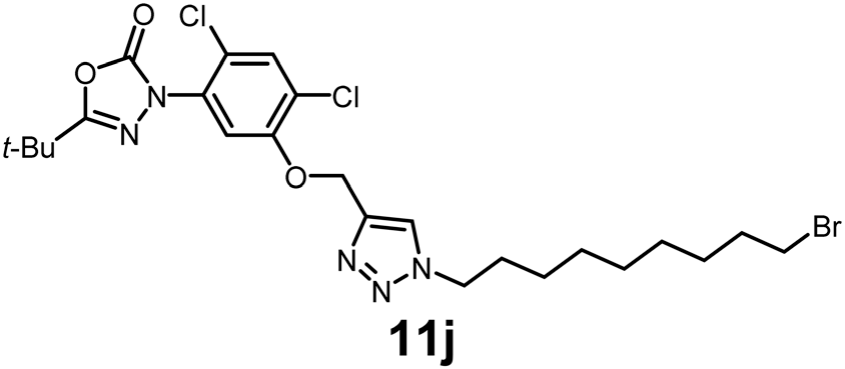


#### 10-(4-((5-(5-(*tert*-Butyl)-2-oxo-1,3,4-oxadiazol-3(2*H*)-yl)-2,4-dichlorophenoxy)methyl)-1*H*-1,2,3-triazol-1-yl)decanenitrile

Off-white viscous oil (98% yield); IR: (neat) *ν* (cm^−1^): 2983, 1747, 1654, 1531, 1495, 1421, 1347, 1204, 1142, 1027, 968, 841, 782, 634, 583; ^1^H NMR (500 MHz, CDCl_3_) *δ* 7.73 (s, 1H), 7.55 (S,1H), 7.26 (s, 1H), 5.32 (s, 2H), 4.37 (m, 2H), 1.92 (t, *J* = 7.1 Hz, 2H), 1.39 (m, 16H), 1.33 (m, 9H); ^13^C{^1^H} NMR (126 MHz, CDCl_3_) *δ* 163.6, 152.9, 152.1, 131.4, 125.1, 123.9, 113.8, 84.8, 83.5, 63.7, 50.6, 33.0, 30.2, 29.2, 29.1, 28.8, 27.0, 26.4; HRMS (ESI-TOF) *m*/*z*: [M + H]^+^ calcd for C_25_H_34_Cl_2_N_5_O_3_ 535.1991; found 535.1991.
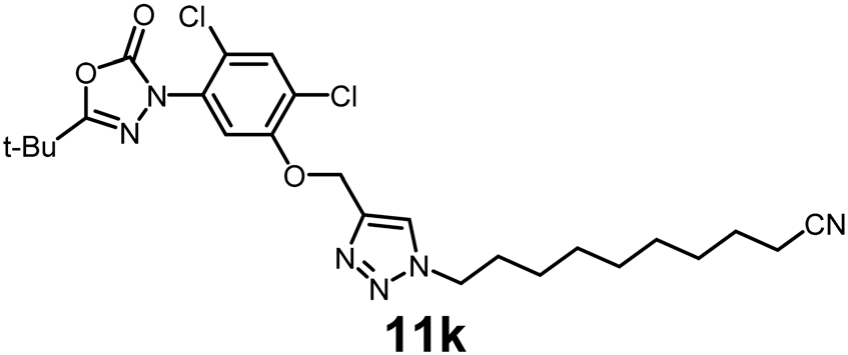


#### 5-(*tert*-Butyl)-3-(2,4-dichloro-5-((1-(9-(oxiran-2-yl)nonyl)-1*H*-1,2,3-triazol-4-yl)methoxy)phenyl)-1,3,4-oxadiazol-2(3*H*)-one

Off-white viscous oil (92% yield); IR: (neat) *ν* (cm^−1^): 2972, 1765, 1643, 1547, 1423, 1463, 1375, 1212, 1174, 1047, 962, 868, 684, 556; ^1^H NMR (500 MHz, CDCl_3_) *δ* 7.68 (s, 1H), 7.53 (s, 1H), 7.27 (s, 1H), 5.31 (s, 2H), 4.36 (t, *J* = 7.3 Hz, 2H), 2.90 (m, 1H), 2.74 (dd, *J* = 5.0, 3.9 Hz, 1H), 2.46 (dd, *J* = 5.0, 2.7 Hz, 1H), 1.96–1.84 (m, 3H), 1.58–1.50 (m, 1H), 1.53–1.44 (m, 1H), 1.38 (s, 9H), 1.34–1.27 (m, 11H); ^13^C{^1^H} NMR (126 MHz, CDCl_3_) *δ* 163.6, 152.9, 152.1, 142.6, 131.4, 125.1, 123.9, 122.9, 113.7, 63.7, 53.5, 50.5, 47.1, 33.0, 32.5, 30.2, 29.4, 29.3, 29.2, 28.9, 27.0, 26.4, 25.9; HRMS (ESI-TOF) *m*/*z*: [M + H]^+^ calcd for C_26_H_37_Cl_2_N_5_O_4_ 552.2144; found 552.2146.
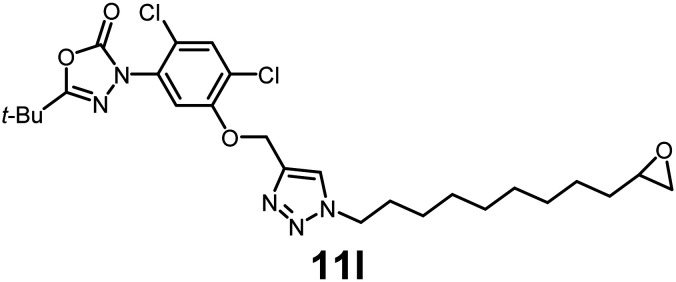


#### 5-(*tert*-Butyl)-3-(2,4-dichloro-5-((1-(10-hydroxy-11-iodoundecyl)-1*H*-1,2,3-triazol-4-yl)methoxy)phenyl)-1,3,4-oxadiazol-2(3*H*)-one

Pale-yellow viscous oil (78% yield); IR: (neat) *ν* (cm^−1^): 3486, 2936, 1762, 1658, 1528, 1453, 1369, 1248, 1136, 1047, 938, 874, 623, 556; ^1^H NMR (500 MHz, CDCl_3_) *δ* 7.68 (s,1H), 7.59–7.49 (s, 1H), 7.32–7.22 (s, 1H), 5.29 (m, 2H), 4.34 (t, *J* = 6.9 Hz, 2H), 3.53–3.46 (m, 1H), 3.35 (m, 1H), 3.22 (m, 1H), 2.50 (m, 1H), 2.08 (m, 1H), 1.90 (m, 2H), 1.59–1.47 (m, 2H), 1.39–1.36 (s, 9H), 1.30–1.25 (m, 11H); ^13^C{^1^H} NMR (126 MHz, CDCl_3_) *δ* 163.6, 152.9, 152.1, 142.6, 131.40, 131.38, 125.1, 123.8, 123.0, 113.8, 70.8, 63.7, 50.5, 36.5, 33.0, 30.1, 29.3, 29.24, 29.17, 28.8, 27.0, 26.4, 25.6, 16.6; HRMS (ESI-TOF) *m*/*z*: [M + H]^+^ calcd for C_26_H_37_ICl_2_N_5_O_4_ 680.1267; found 680.1267.
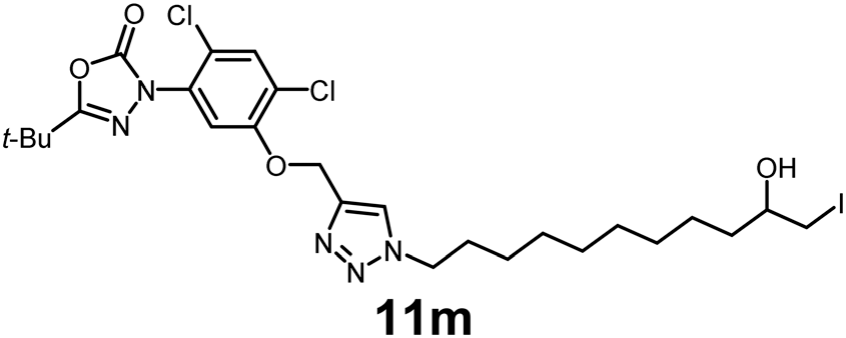


#### 5-(*tert*-Butyl)-3-(2,4-dichloro-5-((1-(11-fluoro-10-hydroxyundecyl)-1*H*-1,2,3-triazol-4-yl)methoxy)phenyl)-1,3,4-oxadiazol-2(3*H*)-one

Pale-yellow viscous oil (82% yield); IR: (neat) *ν* (cm^−1^): 3428, 2983, 1772, 1698, 1538, 1427, 1386, 1234, 1117, 1066, 958, 829, 667, 568, 538; ^1^H NMR (500 MHz, CDCl_3_) *δ* 7.68 (s, 1H), 7.52 (s, 1H), 7.25 (s, 1H), 5.31 (s, 2H), 4.35 (m, 2H), 3.70–3.58 (m, 1H), 3.24 (s, 1H), 1.90 (t, *J* = 7.3 Hz, 3H), 1.60 (m, 3H), 1.57 (m, 1H), 1.37 (s, 9H), 1.26 (m, 11H); ^13^C{^1^H} NMR (126 MHz, CDCl_3_) *δ* 163.6, 152.9, 152.1, 142.6, 131.4, 125.1, 123.9, 122.99, 122.97, 113.7, 71.8 (d, *J*_C–F_ = 107.3 Hz), 66.9, 63.7, 50.5, 34.2, 33.2, 33.1, 33.0, 30.1, 29.6, 29.5, 29.4, 29.2, 29.2, 29.0, 28.80, 28.77, 27.0, 26.7; HRMS (ESI-TOF) *m*/*z*: [M + H]^+^ Calcd for C_26_H_37_Cl_2_FN_5_O_4_ 572.2207; found 572.2209.
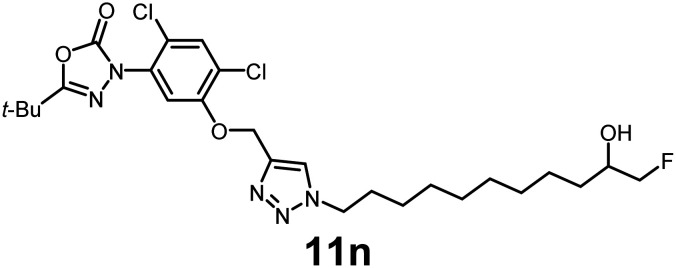


#### 5-(*tert*-Butyl)-3-(5-((1-(11-(*tert*-butylamino)-10-hydroxyundecyl)-1*H*-1,2,3-triazol-4-yl)methoxy)-2,4-dichlorophenyl)-1,3,4-oxadiazol-2(3*H*)-one

Pale-yellow viscous oil (88% yield); IR: (neat) *ν* (cm^−1^): 3486, 3286, 2918, 1763, 1638, 1541, 1463, 1417, 1338, 1239, 1129, 1068, 972, 823, 659, 538, 528; ^1^H NMR (500 MHz, CDCl_3_) *δ* 7.68 (s, 1H), 7.49 (s, 1H), 7.25 (s, 1H), 5.29 (s, 2H), 4.32 (m, 3H), 4.07 (m, 2H), 3.23 (m, 1H), 2.98 (m, 2H), 2.77 (m, 1H), 1.88 (m, 3H), 1.57 (m, 2H), 1.46–1.34 (s, 9H), 1.33–1.17 (m, 18H); ^13^C{^1^H} NMR (126 MHz, CDCl_3_) *δ* 163.6, 153.0, 152.1, 142.5, 131.4, 131.3, 125.0, 123.8, 123.0, 113.7, 66.8, 63.7, 57.2, 51.4, 50.5, 48.4, 34.8, 32.9, 30.2, 29.44, 29.37, 29.30, 29.2, 28.9, 27.0, 26.4, 25.9, 25.4; HRMS (ESI-TOF) *m*/*z*: [M + H]^+^ calcd for C_30_H_47_Cl_2_N_6_O_4_ 625.3036; found 625.3037.
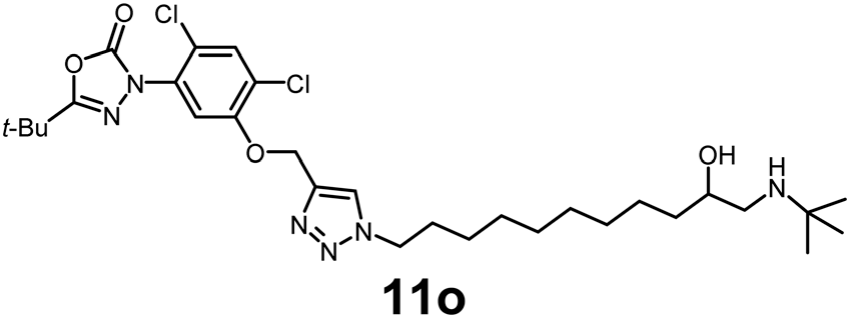


#### 3-(5-((1-(11-Amino-10-hydroxyundecyl)-1*H*-1,2,3-triazol-4-yl)methoxy)-2,4-dichlorophenyl)-5-(*tert*-butyl)-1,3,4-oxadiazol-2(3*H*)-one

See SI for synthesis. Pale-yellow viscous oil (86% yield); IR: (neat) *ν* (cm^−1^): 3562, 3452, 2989, 1763, 1638, 1563, 1428, 1439, 1374, 1269, 1132, 1068, 939, 738, 647, 547; ^1^H NMR (500 MHz, CDCl_3_) *δ* 7.75 (s, 1H), 7.51 (s, 1H), 7.28 (s, 1H), 5.27 (s, 2H), 4.33 (m, 2H), 3.96–3.93 (m, 3H), 3.11 (m, 2H), 2.95 (m, 2H), 1.89 (m, 3H), 1.36 (s, 9H), 1.24 (m, 10H); ^13^C{^1^H} NMR (126 MHz, CDCl_3_) *δ* 163.6, 152.9, 152.2, 142.5, 131.43, 131.36, 125.0, 123.9, 123.3, 113.8, 68.0, 63.6, 50.5, 45.4, 34.9, 33.0, 30.2, 29.5, 29.40, 29.35, 28.9, 27.03, 27.01, 26.4, 25.4; HRMS (ESI-TOF) *m*/*z*: [M + H]^+^ calcd for C_26_H_39_Cl_2_N_6_O_4_ 569.2410; found 569.2412.
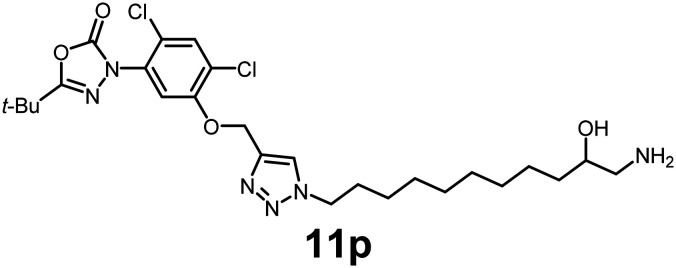


#### 5-(*tert*-Butyl)-3-(2,4-dichloro-5-((1-(10-hydroxy-11-(phenethylamino)undecyl)-1*H*-1,2,3-triazol-4-yl)methoxy)phenyl)-1,3,4-oxadiazol-2(3*H*)-one

Pale-yellow viscous oil (92% yield); IR: (neat) *ν* (cm^−1^): 3486, 3304, 2962, 1736, 1642, 1528, 1414, 1401, 1328, 1236, 1169, 1042, 986, 725, 638, 589, 528; ^1^H NMR (500 MHz, CDCl_3_) *δ* 7.84 (s, 1H), 7.69 (s, 1H), 7.49 (s, 1H), 7.28 (s, 3H), 7.21–7.18 (m, 2H), 5.28 (s, 2H), 4.36–4.27 (m, 2H), 3.20 (m, 1H), 2.91 (m, 2H), 2.87–2.77 (m, 1H), 1.91–1.83 (m, 2H), 1.45 (m, 2H), 1.37 (m, 12H), 1.26 (m, 11H); ^13^C{^1^H} NMR (126 MHz, CDCl_3_) *δ* 163.6, 152.9, 152.1, 142.5, 131.42, 131.35, 128.8, 128.73, 128.71, 128.6, 125.1, 123.8, 123.1, 113.8, 63.6, 53.5, 50.5, 32.9, 30.2, 29.3, 29.2, 28.8, 27.0, 26.4; HRMS (ESI-TOF) *m*/*z*: [M + H]^+^ calcd for C_34_H_47_Cl_2_N_6_O_4_ 673.3036; found 673.3035.
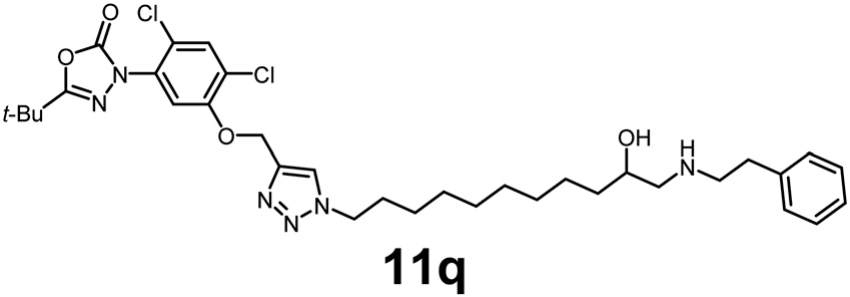


#### 3-(5-((1-(11-(Benzhydrylamino)-10-hydroxyundecyl)-1*H*-1,2,3-triazol-4-yl)methoxy)-2,4-dichlorophenyl)-5-(*tert*-butyl)-1,3,4-oxadiazol-2(3*H*)-one

Pale-yellow viscous oil (90% yield); IR: (neat) *ν* (cm^−1^): 3521, 3289, 2982, 1742, 1632, 1587, 1458, 1456, 1363, 1225, 1174, 1023, 953, 848, 732, 678, 556; ^1^H NMR (500 MHz, CDCl_3_) *δ* 7.67 (s, 1H), 7.57–7.51 (s, 1H), 7.43–7.34 (m, 4H), 7.33–7.19 (m, 6H), 5.29 (s, 2H), 4.84 (m, 1H), 4.33 (t, *J* = 7.3 Hz, 2H), 3.64 (m, 1H), 2.71 (m, 2H), 2.70 (m, 1H), 2.49–2.43 (m, 1H), 1.90 (m, 2H), 1.38 (m, 9H), 1.33–1.23 (m, 13H); ^13^C{^1^H} NMR (126 MHz, CDCl_3_) *δ* 163.6, 153.0, 152.1, 143.7, 143.4, 142.5, 131.4, 128.5, 127.3, 127.22, 127.15, 123.0, 113.8, 70.2, 67.2, 63.7, 53.7, 50.5, 35.0, 33.0, 30.2, 29.6, 29.4, 29.3, 28.9, 27.0, 26.4, 25.6; HRMS (ESI-TOF) *m*/*z*: [M + H]^+^ calcd for C_39_H_49_Cl_2_N_6_O_4_ 735.3192; found 735.3194.
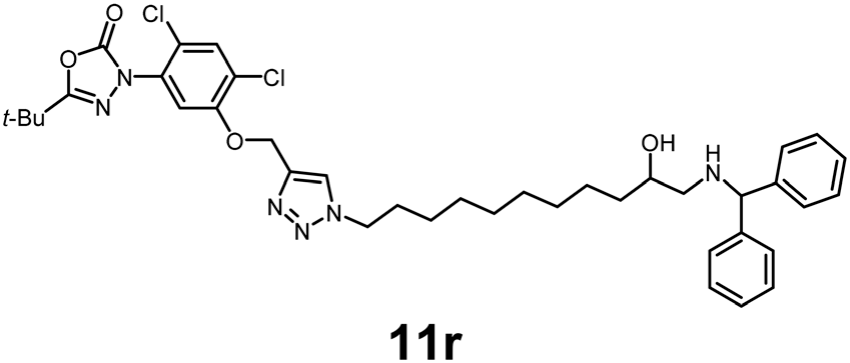


#### Diethyl(3-(4-((5-(5-(*tert*-butyl)-2-oxo-1,3,4-oxadiazol-3(2*H*)-yl)-2,4-dichlorophenoxy)methyl)-1*H*-1,2,3-triazol-1-yl)propyl)phosphonate

Off-white viscous oil (85% yield); IR: (neat) *ν* (cm^−1^): 2923, 1765, 1625, 1586, 1524, 1468, 1345, 1282, 1204, 1156, 923, 865, 745, 571; ^1^H NMR (500 MHz, CDCl_3_) *δ* 7.76 (s, 1H), 7.51 (s, 1H), 7.26–7.22 (s, 1H), 5.29 (s, 2H), 4.48 (m, 2H), 4.09–4.07 (m, 2H), 2.23 (s, 1H), 1.74–1.69 (m, 2H), 1.44–1.34 (s, 9H), 1.34–1.26 (m, 9H); ^13^C{^1^H} NMR (126 MHz, CDCl_3_) *δ* 163.6, 152.9, 152.1, 142.7, 131.41, 131.38, 125.1, 123.9, 123.5, 113.7, 63.6, 61.9, 61.8, 50.2, 50.0, 32.9, 27.0, 23.6, 23.6, 23.0, 21.9, 16.5, 16.4; HRMS (ESI-TOF) *m*/*z*: [M + H]^+^ calcd for C_22_H_31_Cl_2_N_5_O_6_P 562.1389; found 562.1387.
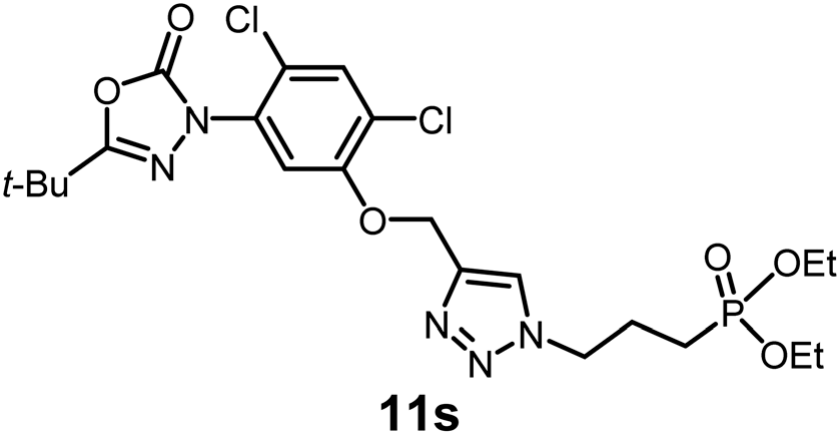


#### Diethyl(4-(4-((5-(5-(*tert*-butyl)-2-oxo-1,3,4-oxadiazol-3(2*H*)-yl)-2,4-dichlorophenoxy)methyl)-1*H*-1,2,3-triazol-1-yl)butyl)phosphonate

Off-white viscous oil (90% yield); IR: (neat) *ν* (cm^−1^): 2962, 1753, 1645, 1634, 1547, 1431, 1367, 1238, 1128, 1063, 975, 852, 762, 612, 537; ^1^H NMR (500 MHz, CDCl_3_) *δ* 7.69 (s, 1H), 7.48 (s, 1H), 7.22 (s, 1H), 5.26 (s, 2H), 4.36 (m, 2H), 4.06–3.98 (m, 2H), 2.70 (m, 1H), 2.66 (s, 9H), 2.43 (m, 11H), 1.33 (t, *J* = 7.4 Hz, 3H); ^13^C{^1^H} NMR (126 MHz, CDCl_3_) *δ* 163.6, 152.9, 152.1, 142.6, 131.4, 125.1, 123.8, 123.2, 113.7, 63.5, 61.63, 61.58, 49.8, 32.9, 30.7, 30.6, 27.0, 25.4, 24.3, 19.62, 19.58, 16.44,16.39; HRMS (ESI-TOF) *m*/*z*: [M + H]^+^ calcd for C_23_H_323_Cl_2_N_5_O_6_P 576.1546; found 576.1546.
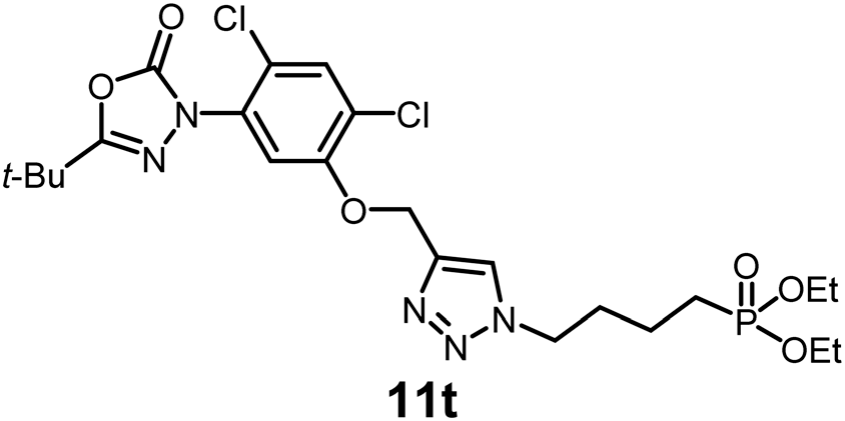


#### Diethyl(5-(4-((5-(5-(*tert*-butyl)-2-oxo-1,3,4-oxadiazol-3(2*H*)-yl)-2,4-dichlorophenoxy)methyl)-1*H*-1,2,3-triazol-1-yl)pentyl)phosphonate

Off-white viscous oil (88% yield); IR: (neat) *ν* (cm^−1^): 2982, 1745, 1636, 1621, 1536, 1458, 1339, 1221, 1172, 1028, 968, 823, 712, 543; ^1^H NMR (500 MHz, CDCl_3_) *δ* 7.68 (s, 1H), 7.54–7.48 (s, 1H), 7.32–7.21 (s, 1H), 5.28 (s, 2H), 4.41–4.31 (m, 2H), 4.13–4.04 (m, 2H), 4.08–3.99 (m, 2H), 1.99–1.88 (m, 2H), 1.76–1.58 (m, 4H), 1.48–1.35 (m, 1H), 1.40–1.33 (s, 9H), 1.34–1.21 (m, 7H); ^13^C{^1^H} NMR (126 MHz, CDCl_3_) *δ* 163.6, 152.9, 152.1, 142.6, 131.4, 125.1, 123.9, 123.0, 113.7, 63.6, 61.54, 61.49, 50.2, 32.9, 29.8, 27.3, 27.2, 27.0, 25.9, 24.8, 22.0, 21.9, 16.5, 16.4; HRMS (ESI-TOF) *m*/*z*: [M + H]^+^ calcd for C_24_H_35_Cl_2_N_5_O_6_P 590.1702; found 590.1704.
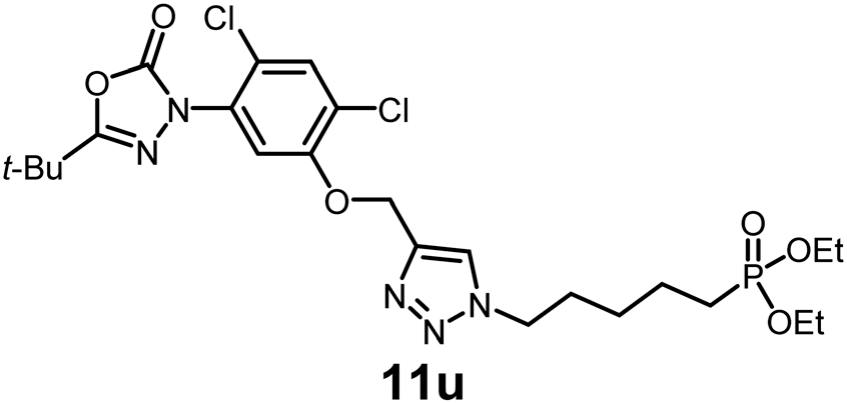


#### (3-(4-((5-(5-(*tert*-Butyl)-2-oxo-1,3,4-oxadiazol-3(2*H*)-yl)-2,4-dichlorophenoxy)methyl)-1*H*-1,2,3-triazol-1-yl)propyl)phosphonic acid

See SI for synthesis. Pale-yellow viscous oil (68% yield); IR: (neat) *ν* (cm^−1^): 2982, 1763, 1672, 1617, 1568, 1439, 1364, 1233, 1127, 1051, 928, 874, 741, 653, 528; ^1^H NMR (500 MHz, CD_3_OD) *δ* 8.27 (s, 1H), 7.67 (s, 1H), 7.65 (s, 1H), 5.33 (s, 2H), 4.57 (m, 2H), 2.11 (m, 3H), 1.84 (m, 3H), 1.40 (s, 9H); ^13^C{^1^H} NMR (126 MHz, CD_3_OD) *δ* 163.5, 153.1, 152.5, 131.6, 130.7, 125.0, 124.9, 123.5, 114.5, 62.6, 32.6, 26.0; HRMS (ESI-TOF) *m*/*z*: [M + H]^+^ calcd for C_18_H_23_Cl_2_N_5_O_6_P 506.0763; found 506.0765.
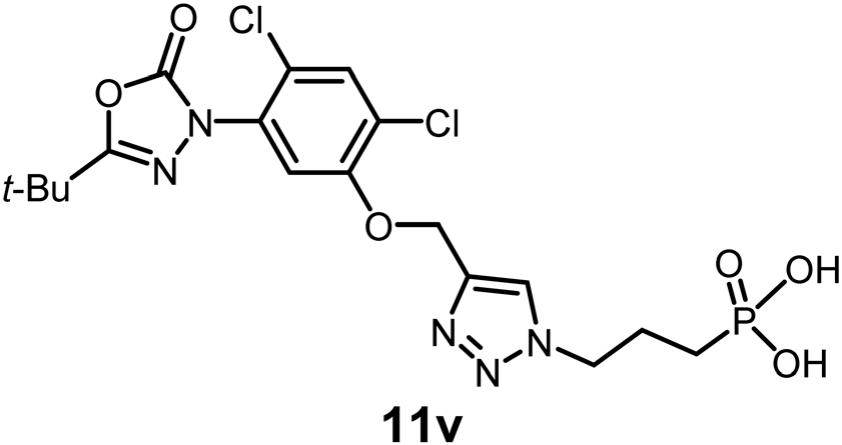


#### (4-(4-((5-(5-(*tert*-Butyl)-2-oxo-1,3,4-oxadiazol-3(2*H*)-yl)-2,4-dichlorophenoxy)methyl)-1*H*-1,2,3-triazol-1-yl)butyl)phosphonic acid

See SI for synthesis. Prepared using the procedure outlined for 19n. pale-yellow viscous oil (74% yield); IR: (neat) *ν* (cm^−1^): 2986, 1772, 1663, 1598, 1523, 1434, 1341, 1232, 1124, 1036, 928, 821, 753, 674, 521; ^1^H NMR (500 MHz, CD_3_OD); *δ* 8.24 (s, 1H), 7.68 (s, 1H), 7.63 (s, 1H), 5.32 (s, 2H), 4.49 (t, *J* = 6.9 Hz, 2H), 2.04 (m, 3H), 1.65 (m, 6H), 1.40 (s, 9H); ^13^C{^1^H} NMR (126 MHz, CD_3_OD) *δ* 163.5, 153.1, 152.5, 142.1, 131.6, 130.7, 124.9, 124.8, 123.7, 114.4, 62.6, 49.9, 32.6, 26.0; HRMS (ESI-TOF) *m*/*z*: [M + H]^+^ calcd for C_19_H_25_Cl_2_N_5_O_6_P 519.0841; found 519.0845.
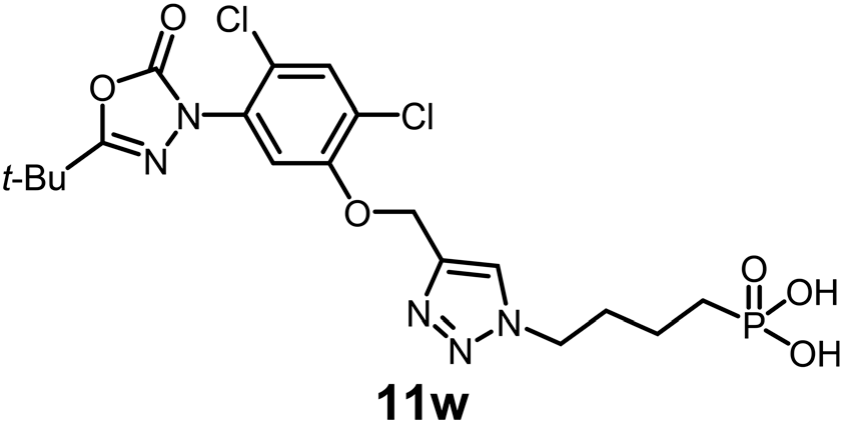


## Conflicts of interest

There is no conflict of interest to declare.

## Supplementary Material

MD-OLF-D5MD00888C-s001

## Data Availability

The data supporting this article have been included as part of the supplementary information (SI). Supplementary information is available. See DOI: https://doi.org/10.1039/d5md00888c.
