# Supplementary material for: Oxadiargyl analogs as potent inhibitors of Toxoplasma gondii protoporphyrinogen oxidase
Source: RSC Med Chem. 2025 Nov 3;17(1):343–53. doi: 10.1039/d5md00888c (PMC12612854; doi:10.1039/d5md00888c)
Supplement: MD-017-D5MD00888C-s001 [file MD-017-D5MD00888C-s001.pdf]

Supplementary Information (SI) for RSC Medicinal Chemistry.
This journal is © The Royal Society of Chemistry 2025

Oxadiargyl Analogs as Potent Inhibitors of *Toxoplasma gondii* Protoporphyrinogen Oxidase

Samuel Kwain,[1,3#] Vikky Awasthi,[3,#] Rajib Islam,[1,3] Shivani Kore,[2,3] Emma Polaski,[2,3] Kerrick C. Rees,[1] Zhicheng Dou,*[2,3] Daniel C. Whitehead*[1,3]

1. Department of Chemistry, Clemson University, Clemson, SC 29634, USA
2. Department of Biological Sciences, Clemson University, Clemson, SC 29634, USA
3. Eukaryotic Pathogens Innovation Center, Clemson University, Clemson, SC 29634, USA

#, both authors made equal contributions to this work.

*Both authors are listed as corresponding authors.

For parasitology: zdou@clemson.edu

For chemistry: dwhiteh@clemson.edu

Table of Contents

1. General Procedure and Analytical Data for Azides **S9a-f** and **S10g-u**……………….. S1

2. Synthesis of Oxadiargyl-triazole derivatives **11p and 11v-w**………………………….. S7

3. Dose-Response Curves for Compounds 11b, 11k, and 11i………………………….. S8

4. NMR spectral data…..……………………………………..………………………… S9

1. General Procedure and Analytical Data for Azides **S9a-f** and **S10g-x**

1.2 Synthesis of azide Intermediates

1-azido-3-methylbutane. In a flame-dried 50 mL round-bottom flask equipped with a magnetic stir bar, 1-bromo-3-methylbutane (3 mmol) was dissolved in 10 mL of anhydrous dimethylformamide (DMF). Sodium azide (5 mmol) was then added, and the reaction mixture was stirred for 24 h. Upon completion of the reaction, 50 mL of deionized water was added. The organic layer was extracted with ethyl acetate (3 × 50 mL), and the combined organic extracts were washed successively with 50 mL of water and 50 mL of saturated brine. The organic layer was dried under reduced pressure, yielding compound **S9a** as an off-white oil with a 74% yield. ^{1}H NMR (500 MHz, $CDCl_3$) δ 3.30 (t, *J* = 7.2 Hz, 2H), 1.72 (dp, *J* = 7.2, 6.7 Hz, 1H), 1.52 (q, *J* = 7.1 Hz, 2H), 0.95 (d, *J* = 6.7 Hz, 6H); ^{13}C{^{1}H} NMR (126 MHz, $CDCl_3$) δ 49.7, 37.4, 25.7, 22.3; HRMS (FTMS+pAPCI) m/z: $[M+H]^+$ Calcd for $C_5H_{12}N_3$ 114.1031; Found 114.1035.

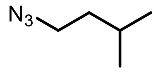

S9a

1-azidoheptane. Prepared following the procedure outlined for compound **S9a**. Off-white oil (85% yield).^{1}H NMR (500 MHz, $CDCl_3$) δ 3.25 (dt, *J* = 6.3, 7.2 Hz, 2H), 1.59 (q, *J* = 7.3 Hz, 1H), 1.45 – 1.25 (m, 9H), 0.93 – 0.86 (m, 3H); ^{13}C{^{1}H} NMR (126 MHz, $CDCl_3$) δ 51.4, 31.7, 28.83, 28.81, 26.7, 22.5, 13.9; HRMS (FTMS+pAPCI) m/z: $[M+H]^+$ Calcd for $C_7H_{16}N_3$ 142.1344; Found 142.1346.

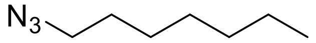

S9b

11-azidoundec-1-ene. Prepared following the procedure outlined for compound **S9a**. Off-white oil (88% yield).^{1}H NMR (500 MHz, $CDCl_3$) δ 5.81 (ddt, *J* = 13.1, 9.9, 6.5 Hz, 1H), 5.04 – 4.90 (m, 2H), 3.26 (q, *J* = 8.6, 6.8 Hz, 2H), 2.08 – 2.01 (m, 2H), 1.61 (dt, *J* = 14.4, 6.5 Hz, 2H), 1.30 (q, *J* = 7.3, 5.6 Hz, 12H); ^{13}C{^{1}H} NMR (126 MHz, $CDCl_3$) δ 139.2, 114.1, 51.5, 33.8, 29.41, 29.36, 29.12, 29.07, 28.9, 28.8, 26.7; HRMS (FTMS+pAPCI) m/z: $[M+H]^+$ Calcd for $C_{11}H_{22}N_3$ 196.1814; Found 196.1818.

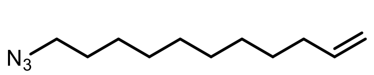

S9c

(4-azidobutyl)benzene. Prepared following the procedure outlined for compound **S9a**. Off-white oil (92% yield).^{1}H NMR (500 MHz, $CDCl_3$) δ 7.54 – 7.31 (m, 5H), 3.38 (m, 2H), 2.80 (dd, *J* = 8.8, 3.7 Hz, 2H), 1.88 – 1.76 (m, 4H); ^{13}C{^{1}H} NMR (126 MHz, $CDCl_3$) δ 142.0, 128.5, 126.1, 51.4, 35.5, 28.61, 28.59; HRMS (FTMS+pAPCI) m/z: $[M+H]^+$ Calcd for $C_{10}H_{14}N_3O$ 176.1188; Found 175.1184.

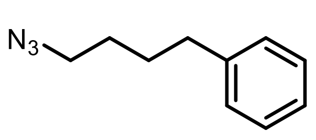

S9d

((4-azidobutoxy)methyl)benzene. In a flame-dried 50 mL round-bottom flask equipped with a magnetic stir bar, 4-(benzyloxy)butan-1-ol (1 mmol) was dissolved in 5 mL of anhydrous DCM at 0 ˚C. To this stirring solution was added triethyl amine (2 mmol) and methanesulfonyl chloride (1.5 mmol). The reaction was stirred at room temperature for 6 h. Upon completion of the reaction, 50 mL of deionized water was added. The organic layer was extracted with ethyl acetate (3 × 50 mL), and the combined organic extracts were washed successively with 50 mL of water and 50 mL of saturated brine. The organic layer was dried and purified by flash chromatography on silica gel, using gradient elution from 100% hexanes to 60% hexanes/ethyl acetate to afford 4-(benzyloxy)butyl methanesulfonate intermediate as a yellow oil (95% yield). In a separate flame-dried 50 mL round-bottom flask equipped with a magnetic stirrer, 4-(benzyloxy)butyl methanesulfonate (1 mmol) was dissolved in 10 mL of DMF, after which sodium azide (2 mmol) was added, and the reaction mixture was allowed to stir at 90 ˚C for 36 h. Upon completion of the reaction, 50 mL of deionized water was added. The organic layer was extracted with ethyl acetate (3 × 50 mL), and the combined organic extracts were washed successively with 50 mL of water and 50 mL of saturated brine. The organic layer was dried and purified by flash chromatography on silica gel, using gradient elution from 100% hexanes to 20% hexanes/ethyl acetate. Compound **S9e** was obtained as a pale-yellow oil in 72% yield. ^{1}H NMR (500 MHz, $CDCl_3$) δ 7.42 – 7.37 (m, 5H), 4.54 (s, 2H), 4.34 (s, 1H), 3.53 (tq, *J* = 5.7, 2.1 Hz, 2H), 3.31 (td, *J* = 6.5, 5.2, 2.9 Hz, 2H), 1.73 (m, 3H); ^{13}C{^{1}H} NMR (126 MHz, $CDCl_3$) δ 138.5, 128.4, 128.3, 127.7, 72.9, 69.6, 51.3, 27.0, 25.9; HRMS (FTMS+pAPCI) m/z: $[M+H]^+$ Calcd for $C_{11}H_{16}N_3O$ 206.1293; Found 206.1296.

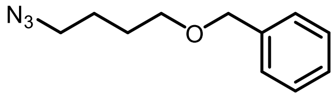

S9e

9-azidononan-1-ol. Prepared following the procedure outlined for compound **S9a**. Off-white oil (90% yield).^{1}H NMR (500 MHz, $CDCl_3$) δ 3.58 (q, *J* = 6.6 Hz, 2H), 3.23 (q, *J* = 7.0 Hz, 2H), 2.31 (d, *J* = 27.4 Hz, 1H), 1.55 (dt, *J* = 13.9, 6.9 Hz, 3H), 1.40 (s, 1H), 1.36 – 1.27 (m, 10H); ^{13}C{^{1}H} NMR (126 MHz, $CDCl_3$) δ 62.7, 51.4, 32.7, 29.4, 29.3, 29.0, 28.8, 26.7, 25.7; HRMS (FTMS+pAPCI) m/z: $[M+H]^+$ Calcd for $C_9H_{20}N_3O$ 186.1606; Found 186.1604.

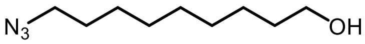

S9f

9-azidononyl methanesulfonate In a flame-dried 50 mL round-bottom flask equipped with a magnetic stir bar, 9-azidononan-1-ol (**S9f**, 1 mmol) was dissolved in 5 mL of anhydrous DCM at 0 °C. To this stirring solution was added triethyl amine (2 mmol) and methanesulfonyl chloride (1.5 mmol). The reaction was stirred at room temperature for 6 h. Upon completion of the reaction, 50 mL of deionized water was added. The organic layer was extracted with ethyl acetate (3 × 50 mL), and the combined organic extracts were washed successively with 50 mL of water and 50 mL of saturated brine. The organic layer was dried and purified by flash chromatography on silica gel, using gradient elution from 100% hexanes to 20% hexanes/ethyl acetate to afford **S10g** as a yellow oil (92% yield). ^{1}H NMR

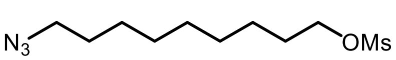

S10g

(500 MHz, $CDCl_3$) δ 4.22 (q, J = 6.5 Hz, 2H), 3.53 (t, J = 6.8 Hz, 1H), 3.25 (t, J = 6.8 Hz, 2H), 3.05 – 2.97 (m, 2H), 1.81 – 1.69 (m, 3H), 1.59 (t, J = 7.1 Hz, 2H), 1.33 (dd, J = 12.7, 7.0 Hz, 10H); $^{13}C\{^1H\}$ NMR (126 MHz, $CDCl_3$) δ 70.2, 51.4, 37.3, 29.2, 29.1, 29.0, 28.9, 28.8, 26.6, 25.4; HRMS (FTMS+pAPCI) m/z: $[M+H]^+$ Calcd for $C_{10}H_{22}N_3O_3S$ 264.1382; Found 264.1386.

9-azidononyl pivalate. In a flame-dried 50 mL round-bottom flask equipped with a magnetic stir bar, 9-azidononan-1-ol (**S9f**, 1 mmol) was dissolved in 5 mL of anhydrous DCM at 0 ˚C. To this stirring solution was added triethyl amine (2 mmol) followed by pivaloyl chloride (2 mmol) and the reaction was stirred at room temperature for 6 h. Upon completion of the reaction, 50 mL of deionized water was added. The organic layer was extracted with DCM (3 × 50 mL), and the combined organic extracts were washed successively with 50 mL of water and 50 mL of saturated brine. The organic layer was dried and purified by flash chromatography on silica gel, using gradient elution from 100% hexanes to 10% hexanes/ethyl acetate. Compound **S10h** was obtained as a pale-yellow oil in 85% yield. ^1H NMR (500 MHz, $CDCl_3$) δ 3.95 (q, J = 6.7 Hz, 1H), 3.50 (q, J = 6.7 Hz, 1H), 3.15 (t, J = 7.0 Hz, 2H), 1.49 (ddp, J = 20.9, 14.1, 6.9, 6.3 Hz, 5H), 1.25 (dd, J = 18.5, 7.5 Hz, 13H), 1.10 (d, J = 6.2 Hz, 5H); $^{13}C\{^1H\}$ NMR (126 MHz, $CDCl_3$) δ 178.4, 64.3, 51.3, 38.6, 32.6, 29.4, 29.2, 29.0, 28.7, 27.0, 26.6, 25.7; HRMS (FTMS+pAPCI) m/z: $[M+H]^+$ Calcd for $C_{14}H_{28}N_3O_2$ 270.2182; Found 270.2186.

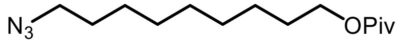

S10h

1-azido-9-fluorononane. In a flame-dried 50 mL round-bottom flask equipped with magnetic stirrer, compound **S10g** (1 mmol) was dissolved in 10 mL of DMF after which cesium fluoride (2 mmol) was added and the reaction mixture allowed to stirred at 90 ˚C for 36 h. Upon completion of the reaction, 50 mL of deionized water was added. The organic layer was extracted with ethyl acetate (3 × 50 mL), and the combined organic extracts were washed successively with 50 mL of water and 50 mL of saturated brine. The organic layer was dried and purified by flash chromatography on silica gel, using gradient elution from 100% hexanes to 10% hexanes/ethyl acetate. Compound **S10i** was obtained as a pale-yellow oil in 82% yield. ^1H NMR (500 MHz, $CDCl_3$) δ 3.49 (t, J = 6.8 Hz, 2H), 3.22 (t, J = 7.0 Hz, 2H), 1.80 – 1.68 (m, 2H), 1.56 (m, H), 1.44 – 1.25 (m, 10H); $^{13}C\{^1H\}$ NMR (126 MHz, $CDCl_3$) δ 84.0 (d, $J_{C\text{-}F}$ = 164.1 Hz), 51.4, 32.6, 29.30, 29.25, 29.0, 28.8, 26.8, 26.6; HRMS (FTMS+pAPCI) m/z: $[M+H]^+$ Calcd for $C_9H_{19}FN_3$ 188.1563; Found 188.1566.

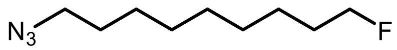

S10i

1-azido-9-bromononane. Prepared following the procedure outlined for compound **S10i**, except cesium fluoride was substituted for sodium bromide. Off-white oil (72% yield).^1H NMR (500 MHz, $CDCl_3$) δ 3.49 (dd, J = 7.8, 5.3 Hz, 1H), 3.37 (dt, J = 9.4, 6.6 Hz, 1H), 3.22 (q, J = 7.4 Hz, 2H), 1.82 (h, J = 7.3, 5.7 Hz, 2H), 1.73 (p, J = 7.0 Hz, 1H), 1.56 (t, J = 7.2 Hz, 1H), 1.44 – 1.36 (m, 3H), 1.37 – 1.25

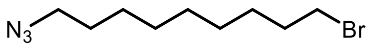

S10j

(m, 7H); $^{13}C\{^1H\}$ NMR (126 MHz, $CDCl_3$) δ 51.4, 33.8, 32.8, 29.3, 29.0, 28.8, 28.6, 28.1, 26.6; HRMS (FTMS+pAPCI) m/z: $[M+H]^+$ Calcd for $C_9H_{19}BrN_3$ 248.0762; Found 248.0766.

10-azidodecanenitrile. Prepared following the procedure outlined for compound **S10i**, except cesium fluoride was substituted for potassium cyanide. Off-white oil (68% yield). ^1H NMR (500 MHz, $CDCl_3$) δ 4.38 (t, J = 6.2 Hz, 2H), 3.26 (t, J = 7.0 Hz, 7H), 1.83 – 1.54 (m, 13H), 1.43 – 1.29 (m, 36H); $^{13}C\{^1H\}$ NMR (126 MHz, $CDCl_3$) δ 84.8, 51.44, 29.3, 29.12, 29.10, 29.02, 28.97, 28.80, 28.75, 26.7; HRMS (FTMS+pAPCI) m/z: $[M+H]^+$ Calcd for $C_{10}H_{19}N_4$ 195.1610; Found 195.1612.

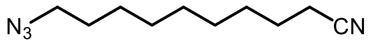

S10k

2-(9-azidononyl)oxirane. In a flame-dried 50 mL round-bottom flask equipped with a magnetic stir bar, 11-azidoundec-1-ene (**S9c**, 1 mmol) was dissolved in 5 mL of anhydrous DCM at 0 ˚C. To this stirring solution was added *m*CPBA (2 mmol) and the reaction was stirred at room temperature for 16 h. Upon completion of the reaction, 50 mL of deionized water was added. The organic layer was extracted with DCM (3 × 50 mL), and the combined organic extracts were washed successively with 50 mL of water and 50 mL of saturated brine. The organic layer was dried and purified by flash chromatography on silica gel, using gradient elution from 100% hexanes to 20% hexanes/ethyl acetate. Compound **S10l** was obtained as a pale-yellow oil in 84% yield. ^1H NMR (500 MHz, $CDCl_3$) δ 4.05 (t, J = 6.7 Hz, 1H), 3.25 (t, J = 6.9, 4.0 Hz, 2H), 2.29 (t, J = 7.5 Hz, 1H), 1.67 – 1.53 (m, 5H), 1.34 (dt, J = 16.5, 6.4 Hz, 12H); $^{13}C\{^1H\}$ NMR (126 MHz, $CDCl_3$) δ 52.4, 51.4, 47.1, 32.5, 29.41, 29.37, 29.35, 29.1, 28.8, 26.7, 26.0; HRMS (FTMS+pAPCI) m/z: $[M+H]^+$ Calcd for $C_{11}H_{22}FN_3O$ 212.1763; Found 212.1766.

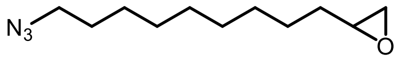

S10l

11-azido-1-iodoundecan-2-ol. In a flame-dried 50 mL round-bottom flask equipped with magnetic stir bar, **S10l** (1 mmol) was dissolved in 10 mL of DMF/H_2O (3:1) solution after which potassium iodide (2 mmol) was added and the reaction mixture allowed to stirred at room temperature for 24 h. Upon completion of the reaction, 50 mL of deionized water was added. The organic layer was extracted with ethyl acetate (3 × 50 mL), and the combined organic extracts were washed successively with 50 mL of water and 50 mL of saturated brine. The organic layer was dried and purified by flash chromatography on silica gel, using gradient elution from 100% hexanes to 80% hexanes/ethyl acetate. Compound **S10m** was obtained as a pale-yellow oil in 64% yield. ^1H NMR (500 MHz, $CDCl_3$) 3.55 – 3.45 (m, 1H), 3.36 (dd, J = 10.2, 3.7 Hz, 1H), 3.27 – 3.18 (m, 3H), 2.30 (d, J = 5.4 Hz, 1H), 1.63 – 1.50 (m, 3H), 1.44 – 1.26 (m, 13H); $^{13}C\{^1H\}$ NMR (126 MHz, $CDCl_3$) δ 70.9, 51.5, 36.6, 29.40, 29.37, 29.37, 29.1, 28.8, 26.7, 25.6, 16.7; HRMS (FTMS+pAPCI) m/z: $[M+H]^+$ Calcd for $C_{11}H_{23}IN_3O$ 340.0886; Found 340.0882.

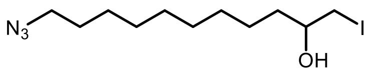

S10m

11-azido-1-fluoroundecan-2-ol. Prepared following the procedure outlined for compound **S10m**, except potassium iodide was substituted for cesium fluoride. Pale yellow oil (68% yield).^{1}H NMR (500 MHz, $CDCl_3$) δ 3.73 – 3.58 (m, 1H), 3.45 (ddd, J = 29.3, 11.0, 7.3 Hz, 1H), 3.24 (d, J = 6.9 Hz, 2H), 1.59 (p, J = 7.0 Hz, 3H), 1.36 (d, J = 16.1 Hz, 1H), 1.35 (s, 3H), 1.30 (m, 11H); $^{13}C\{^1H\}$ NMR (126 MHz, $CDCl_3$) δ 87.0 (d, J_{C-F} = 168.6 Hz), 72.3, 51.4, 34.2, 33.1, 29.6, 29.32, 29.31, 29.1, 28.8, 26.7; HRMS (FTMS+pAPCI) m/z: $[M+H]^+$ Calcd for $C_{11}H_{23}FN_3O$ 232.1825; Found 232.1828.

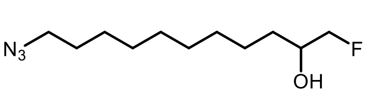

S10n

11-azido-1-(*tert*-butylamino)undecan-2-ol. In a flame-dried 50 mL round-bottom flask equipped with magnetic stir bar, **S10l** (1 mmol) was dissolved in 10 mL of 1,4-dioxane/H_2O (1:1) solution after which 2-methylpropan-2-amine (2 mmol) was added and the reaction mixture allowed to stirred at 0 ˚C for 16 h. Upon completion of the reaction, 50 mL of deionized water was added. The organic layer was extracted with ethyl acetate (3 × 50 mL), and the combined organic extracts were washed successively with 50 mL of water and 50 mL of saturated brine. The organic layer was dried and purified by flash chromatography on silica gel, using gradient elution from 100% DCM to 5% DCM/MeOH. Compound **S10o** was obtained as a pale-yellow oil in 74% yield. ^{1}H NMR (500 MHz, $CDCl_3$) 4.10 – 3.97 (m, 1H), 3.27 (q, J = 7.6, 6.9 Hz, 3H), 2.96 (dd, J = 12.0, 2.4 Hz, 1H), 2.72 (q, J = 11.7, 8.8 Hz, 1H), 1.61 (t, J = 12.2 Hz, 2H), 1.54 – 1.45 (m, 1H), 1.44 (s, 5H), 1.41 – 1.24 (m, 17H); $^{13}C\{^1H\}$ NMR (126 MHz, $CDCl_3$) δ 67.3, 56.1, 51.5, 48.4, 34.9, 29.5, 29.4, 29.1, 28.8, 26.7, 26.4, 25.5; HRMS (FTMS+pAPCI) m/z: $[M+H]^+$ Calcd for $C_{15}H_{31}N_4O$ 285.2654; Found 285.2652.

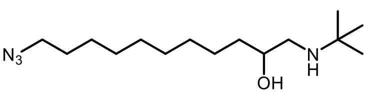

S10o

11-azido-1-(phenethylamino)undecan-2-ol. Prepared following the procedure outlined for compound **S10o**, except 2-methylpropan-2-amine was substituted for 2-phenylethan-1-amine. Pale yellow oil (78% yield).^{1}H NMR (500 MHz, $CDCl_3$) δ 7.37 – 7.29 (m, 2H), 7.23 (tt, J = 7.9, 1.6 Hz, 3H), 3.27 (td, J = 6.9, 2.0 Hz, 3H), 2.94 – 2.77 (m, 3H), 2.75 (dd, J = 12.0, 3.0 Hz, 1H), 2.50 – 2.42 (m, 1H), 1.64 – 1.59 (m, 2H), 1.37 (s, 6H), 1.31 – 1.27 (m, 10H); $^{13}C\{^1H\}$ NMR (126 MHz, $CDCl_3$) δ 139.7, 128.7, 128.5, 126.3, 69.3, 55.1, 51.5, 50.8, 36.3, 35.1, 29.7, 29.5, 29.4, 29.1, 28.8, 26.7, 25.7; HRMS (FTMS+pAPCI) m/z: $[M+H]^+$ Calcd for $C_{19}H_{33}N_4O$ 333.2654; Found 333.2656.

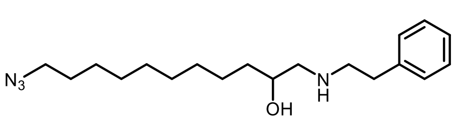

S10q

11-azido-1-(benzhydrylamino)undecan-2-ol. Prepared following the procedure outlined for compound **S10o**, except 2-methylpropan-2-amine was substituted for 2-phenylethan-1-amine. Pale yellow oil (78% yield).^{1}H NMR (500 MHz, $CDCl_3$) δ 7.40 (m, 4H), 7.33 (m, 4H), 7.27 – 7.21 (m, 2H), 4.87 (s, 1H),

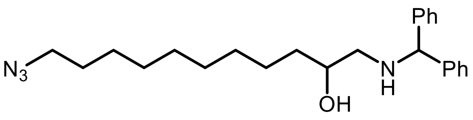

S10r

3.70 – 3.60 (m, 1H), 3.27 (t, *J* = 6.9 Hz, 2H), 2.73 (dd, *J* = 11.9, 3.0 Hz, 1H), 2.47 (dd, *J* = 12.0, 9.0 Hz, 1H), 1.61 (p, *J* = 7.0 Hz, 2H), 1.49 – 1.31 (m, 3H), 1.30 (s, 11H); $^{13}C\{^1H\}$ NMR (126 MHz, $CDCl_3$) δ 143.9, 143.5, 128.6, 127.3, 127.23, 127.16, 127.14, 70.3, 67.1, 53.8, 51.5, 35.0, 29.6, 29.44, 29.41, 29.1, 28.8, 26.7, 25.6; HRMS (FTMS+pAPCI) m/z: $[M+H]^+$ Calcd for $C_{24}H_{35}N_4O$ 395.2811; Found 395.2814.

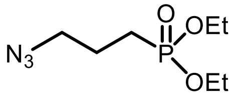

S10s

diethyl (3-azidopropyl)phosphonate. In a flame-dried 50 mL round-bottom flask equipped with a magnetic stirrer, 1,3-dibromopropane (3 mmol) and triethyl phosphite (1 mmol) were added and stirred at 90 ˚C for 16 h. Upon completion of the reaction, the crude mixture was purified by flash chromatography on silica gel, using gradient elution from 100% hexanes to 80% hexanes/ethyl acetate to afford diethyl (2-bromoethyl)phosphonate intermediate in 87% yield. The conversion of diethyl (2-bromoethyl)phosphonate to compound **S10s** followed the same procedure outlined for compound **S9a**. Compound **S10s** was obtained as a pale-yellow oil in 68% yield. ^1H NMR (500 MHz, $CDCl_3$) δ 3.92 – 3.75 (m, 5H), 3.13 (t, *J* = 6.5 Hz, 2H), 1.73 – 1.46 (m, 3H), 1.06 (m, 6H); $^{13}C\{^1H\}$ NMR (126 MHz, $CDCl_3$) δ 61.4, 61.3, 51.2, 51.1, 23.1, 22.2, 22.1, 22.0, 16.2, 16.1; HRMS (FTMS+pAPCI) m/z: $[M+H]^+$ Calcd for $C_7H_{17}N_3O_3P$ 222.1008; Found 222.1004.

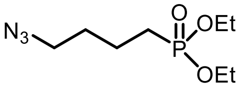

S10t

diethyl (4-azidobutyl)phosphonate. Prepared following the procedure outlined for compound **S10s**, except 1,3-dibromopropane was substituted for 1,4-dibromobutane. Pale yellow oil (78% yield). ^1H NMR (500 MHz, $CDCl_3$) δ 4.02 – 3.89 (m, 4H), 3.22 – 3.13 (m, 2H), 1.70 – 1.55 (m, 4H), 1.55 (dd, *J* = 6.6, 3.4 Hz, 2H), 1.19 (ddd, *J* = 11.1, 7.7, 5.2 Hz, 6H); $^{13}C\{^1H\}$ NMR (126 MHz, $CDCl_3$) δ 61.42, 61.37, 50.7, 29.5, 29.3, 25.6, 24.4, 19.8, 19.7, 16.32, 16.27; HRMS (FTMS+pAPCI) m/z: $[M+H]^+$ Calcd for $C_8H_{19}N_3O_3P$ 236.1164; Found 236.1168.

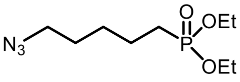

S10u

diethyl (5-azidopentyl)phosphonate. Prepared following the procedure outlined for compound **S10s**, except 1,3-dibromopropane was substituted for 1,5-dibromobutane. Pale yellow oil (74% yield). ^1H NMR (500 MHz, $CDCl_3$) δ 3.64 – 3.50 (m, 4H), 2.78 (q, *J* = 6.6 Hz, 2H), 1.22 (dd, *J* = 15.9, 7.2 Hz, 2H), 1.13 (ddd, *J* = 21.4, 17.4, 9.7 Hz, 4H), 1.02 – 0.93 (m, 2H), 0.81 (q, *J* = 6.9 Hz, 6H); $^{13}C\{^1H\}$ NMR (126 MHz, $CDCl_3$) δ 60.8, 60.7, 50.6, 27.9, 27.1, 27.0, 25.5, 24.4, 21.59, 21.55, 15.90, 15.85; HRMS (FTMS+pAPCI) m/z: $[M+H]^+$ Calcd for $C_9H_{21}N_3O_3P$ 250.1321; Found 250.1325.

2. Synthesis of Oxadiargyl-triazole derivatives 11p and 11v-w

3-(5-((1-(11-amino-10-hydroxyundecyl)-1*H*-1,2,3-triazol-4-yl)methoxy)-2,4-dichlorophenyl)-5-(*tert*-butyl)-1,3,4-oxadiazol-2(3*H*)-one. In a flame-dried 50 mL round-bottom flask equipped with a magnetic stir bar, compound **11l** (1 mmol) was dissolved in 10 mL of DMF, and sodium azide (2 mmol) was added. The reaction mixture was stirred at 90 °C for 16 h. After completion, the reaction was quenched by adding 50 mL of deionized water. The mixture was extracted with ethyl acetate (3 × 50 mL), and the combined organic layers were washed successively with 50 mL of water and 50 mL of saturated brine. The organic phase was dried, yielding a pale-yellow viscous oil corresponding to the azide intermediate, which was used in the subsequent step without further purification. In a separate flame-dried 25 mL round-bottom flask equipped with a magnetic stir bar, the azide intermediate (1 mmol) was dissolved in 5 mL of wet methanol. Pearlman's catalyst (0.2 mmol) was then added, and the flask was sealed and secured with parafilm to ensure airtightness. The reaction vessel was evacuated under vacuum, and a hydrogen balloon was introduced through the septum. The mixture was stirred at room temperature for 2 h. Upon completion, the solvent was evaporated, and the crude product was purified by flash chromatography on silica gel using a gradient elution from 100% hexanes to 60% hexanes/ethyl acetate. Compound **11p** was obtained as a pale-yellow viscous oil (86% yield); IR: (neat) ν (cm^{-1}): 3562, 3452, 2989, 1763, 1638, 1563, 1428, 1439, 1374, 1269, 1132, 1068, 939, 738, 647, 547; ^{1}H NMR (500 MHz, $CDCl_3$) δ 7.75 (s, 1H), 7.51 (s, 1H), 7.28 (s, 1H), 5.27 (s, 2H), 4.33 (m, 2H), 3.96 – 3.93 (m, 3H), 3.11 (m, 2H), 2.95 (m, 2H), 1.89 (m, 3H), 1.36 (s, 9H), 1.24 (m, 10H); $^{13}C\{^{1}H\}$ NMR (126 MHz, $CDCl_3$) δ 163.6, 152.9, 152.2, 142.5, 131.43, 131.36, 125.0, 123.9, 123.3, 113.8, 68.0, 63.6, 50.5, 45.4, 34.9, 33.0, 30.2, 29.51 29.40, 29.35, 28.9, 27.03, 27.01, 26.43, 25.41; HRMS (ESI–TOF) m/z: $[M+H]^+$ Calcd for $C_{26}H_{39}Cl_2N_6O_4$ 569.2410; Found 569.2412.

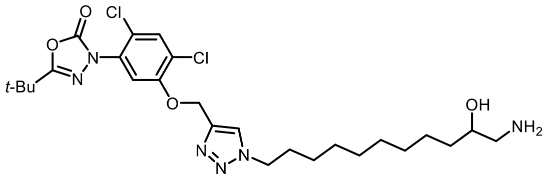

11p

(3-(4-((5-(5-(*tert*-butyl)-2-oxo-1,3,4-oxadiazol-3(2H)-yl)-2,4-dichlorophenoxy)methyl)-1H-1,2,3-triazol-1-yl)propyl)phosphonic acid. In a flame-dried 25 mL round-bottom flask equipped with a magnetic stirrer, compound **11s** (1 mmol) was dissolved in 5 mL of DCM at 0 ˚C. Bromotrimethylsilane (3 mmol) was added dropwise to the solution, and the reaction mixture was stirred at room temperature for 4 h. Upon completion of the reaction, 5 mL of methanol was added, and the mixture was evaporated under reduced pressure. This process was repeated two additional times. The resulting crude material was purified by reverse-phase chromatography on C-18 silica gel using a gradient elution system, starting with 100% water and transitioning to

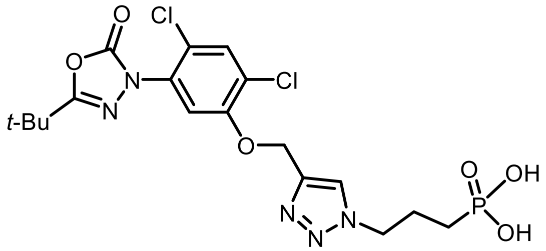

11v

15% water in methanol. The product was isolated by rotary evaporation. The target compound **11v** was obtained as a pale-yellow viscous oil (68% yield); IR: (neat) ν (cm^{-1}): 2982, 1763, 1672, 1617, 1568, 1439, 1364, 1233, 1127, 1051, 928, 874, 741, 653, 528; ^{1}H NMR (500 MHz, CD_3OD) δ 8.27 (s, 1H), 7.67 (s, 1H), 7.65 (s, 1H), 5.33 (s, 2H), 4.57 (m, 2H), 2.11 (m, 3H), 1.84 (m, 3H), 1.40 (s, 9H); $^{13}C\{^{1}H\}$ NMR (126 MHz, CD_3OD) δ 163.5, 153.1, 152.5, 131.6, 130.7, 125.0, 124.9, 123.5, 114.5, 62.6, 32.6, 26.0; HRMS (ESI–TOF) m/z: $[M+H]^+$ Calcd for $C_{18}H_{23}Cl_2N_5O_6P$ 506.0763; Found 506.0765.

(4-(4-((5-(5-(*tert*-butyl)-2-oxo-1,3,4-oxadiazol-3(2H)-yl)-2,4-dichlorophenoxy)methyl)-1H-1,2,3-triazol-1-yl)butyl)phosphonic acid. Prepared using the procedure outlined for **11v** using compound **11t** as starting material to provide **11w** as a pale-yellow viscous oil in 74% yield. ^{1}H NMR (500 MHz, CD_3OD); IR: (neat) ν (cm^{-1}): 2986, 1772, 1663, 1598, 1523, 1434, 1341, 1232, 1124, 1036, 928, 821, 753, 674, 521; δ 8.24 (s, 1H), 7.68 (s, 1H), 7.63 (s, 1H), 5.32 (s, 2H), 4.49 (t, *J* = 6.9 Hz, 2H), 2.04 (m, 3H), 1.65 (m, 6H), 1.40 (s, 9H); $^{13}C\{^{1}H\}$ NMR (126 MHz, CD_3OD) δ 163.5, 153.1, 152.5, 142.1, 131.6, 130.7, 124.9, 124.8, 123.6, 114.4, 62.6, 49.9, 32.6, 26.0; HRMS (ESI–TOF) m/z: $[M+H]^+$ Calcd for $C_{19}H_{25}Cl_2N_5O_6P$ 519.0841; Found 519.0845.

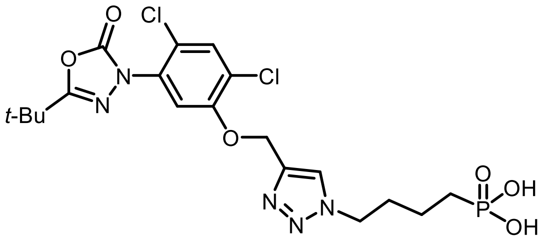

11w

3. Dose Response Curves for Compounds 11b, 11i, and 11k.

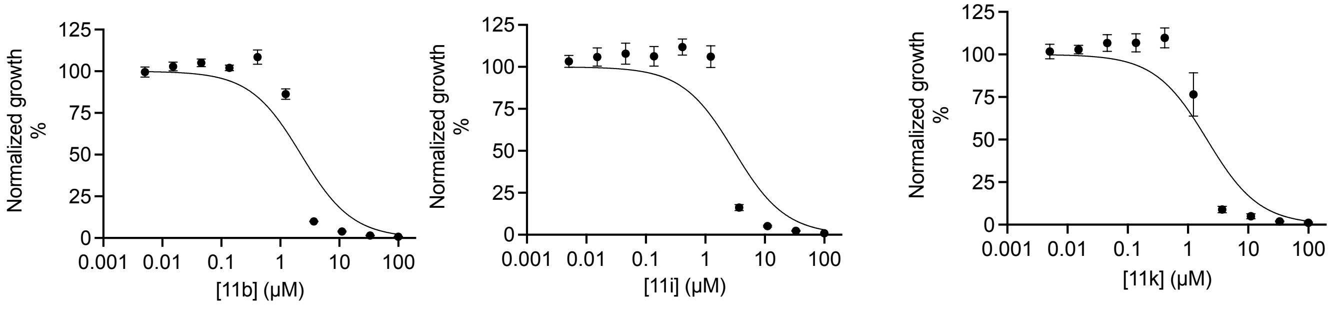

Figure S1. Dose Response Curves for Compounds **11b**, **11i**, and **11k**.

4. NMR spectral data

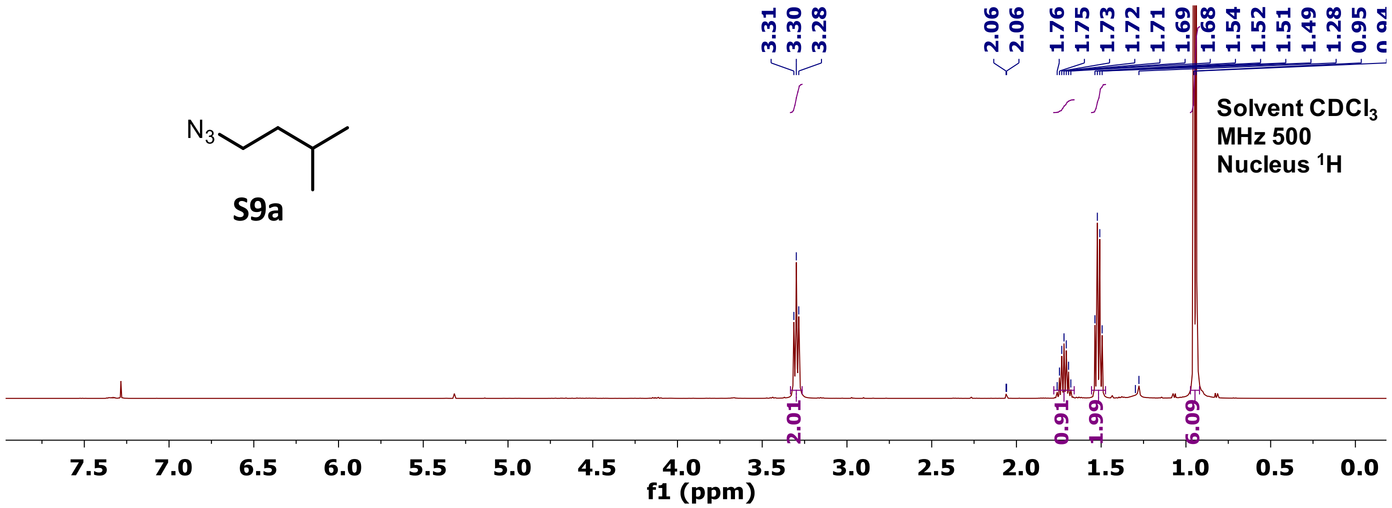

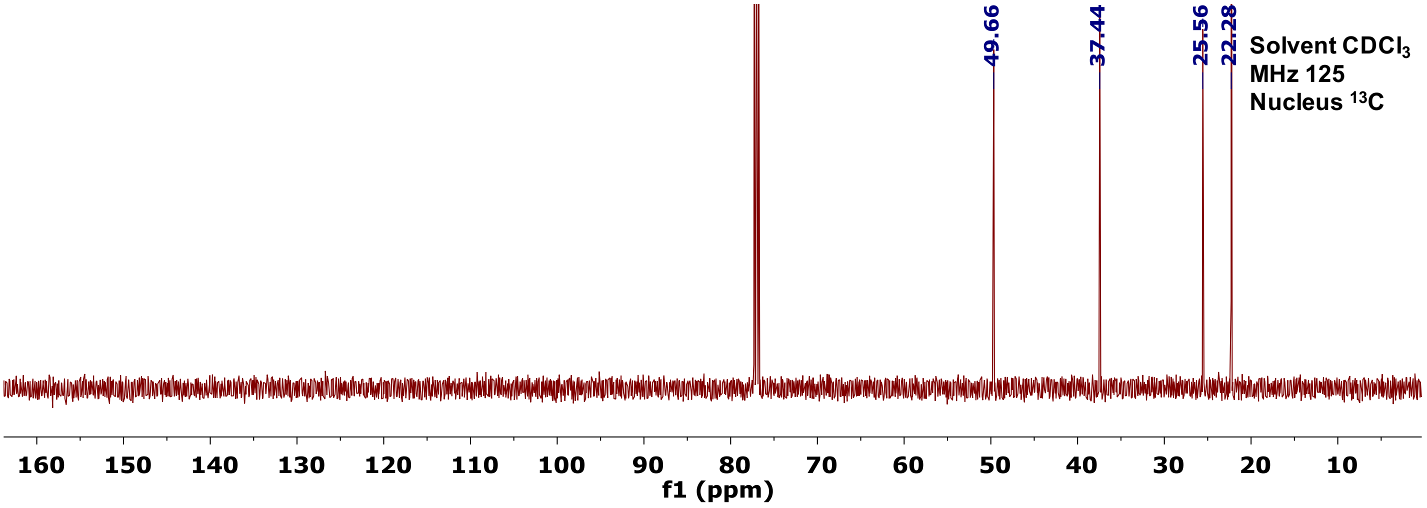

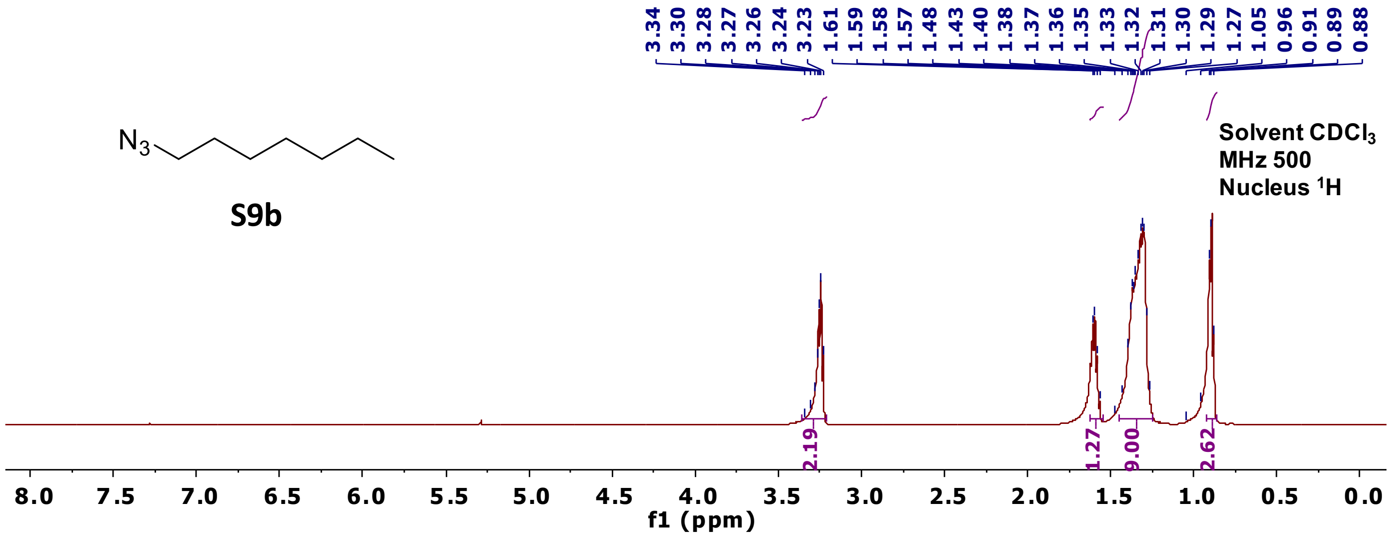
N3
3.34
3.30
3.28
3.27
3.26
3.24
3.23
1.61
1.59
1.58
1.57
1.48
1.43
1.40
1.38
1.37
1.36
1.35
1.33
1.32
1.31
1.30
1.29
1.27
1.05
0.96
0.91
0.89
0.88
Solvent CDCl3
MHz 500
Nucleus 1H
2.19
1.27
9.00
2.62
8.0 7.5 7.0 6.5 6.0 5.5 5.0 4.5 4.0 3.5 3.0 2.5 2.0 1.5 1.0 0.5 0.0
f1 (ppm)

S9b

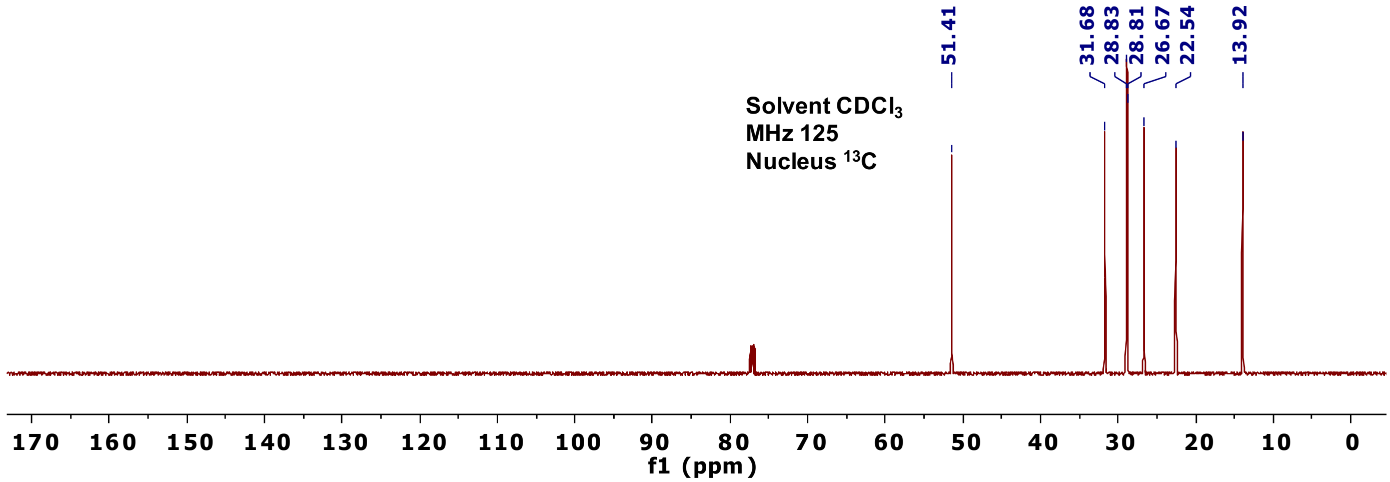
51.41
31.68
28.83
28.81
26.67
22.54
13.92
Solvent CDCl3
MHz 125
Nucleus 13C
170 160 150 140 130 120 110 100 90 80 70 60 50 40 30 20 10 0
f1 (ppm)

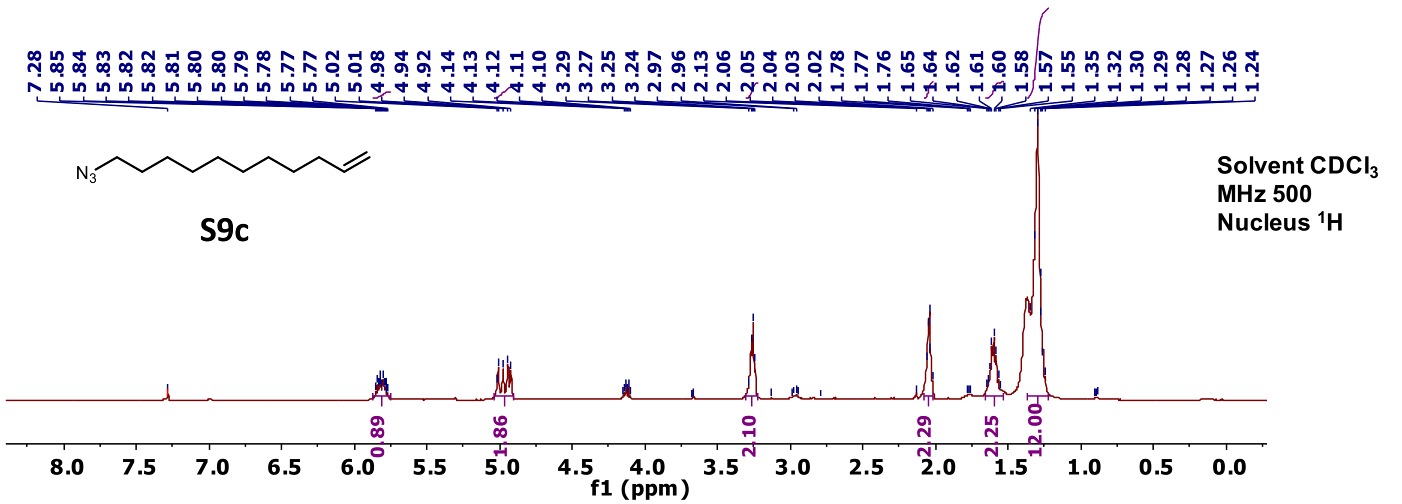
7.28
5.85
5.84
5.83
5.82
5.82
5.81
5.80
5.80
5.79
5.78
5.77
5.77
5.02
5.01
4.98
4.94
4.92
4.14
4.13
4.12
4.11
4.10
3.29
3.27
3.25
3.24
2.97
2.96
2.13
2.06
2.05
2.04
2.03
2.02
1.78
1.77
1.76
1.65
1.64
1.62
1.61
1.60
1.58
1.57
1.55
1.35
1.32
1.30
1.29
1.28
1.27
1.26
1.24
N3
S9c
Solvent CDCl3
MHz 500
Nucleus 1H
0.89
1.86
2.10
2.29
2.25
12.00
8.0 7.5 7.0 6.5 6.0 5.5 5.0 4.5 4.0 3.5 3.0 2.5 2.0 1.5 1.0 0.5 0.0
f1 (ppm)

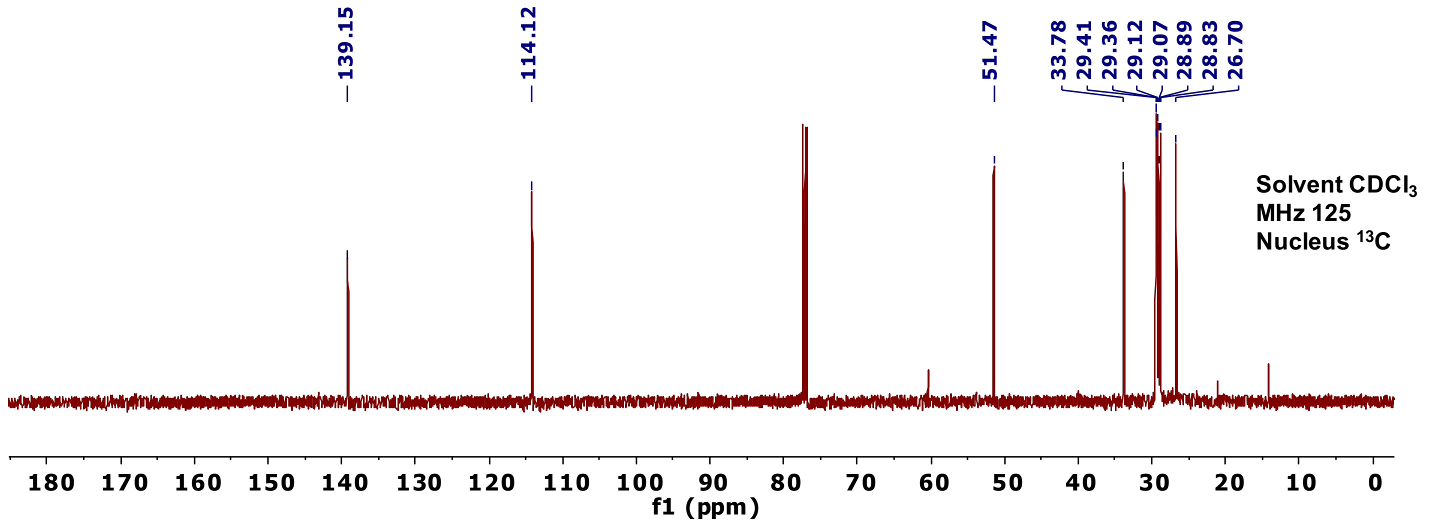
139.15
114.12
51.47
33.78
29.41
29.36
29.12
29.07
28.89
28.83
26.70
Solvent CDCl3
MHz 125
Nucleus 13C
180 170 160 150 140 130 120 110 100 90 80 70 60 50 40 30 20 10 0
f1 (ppm)

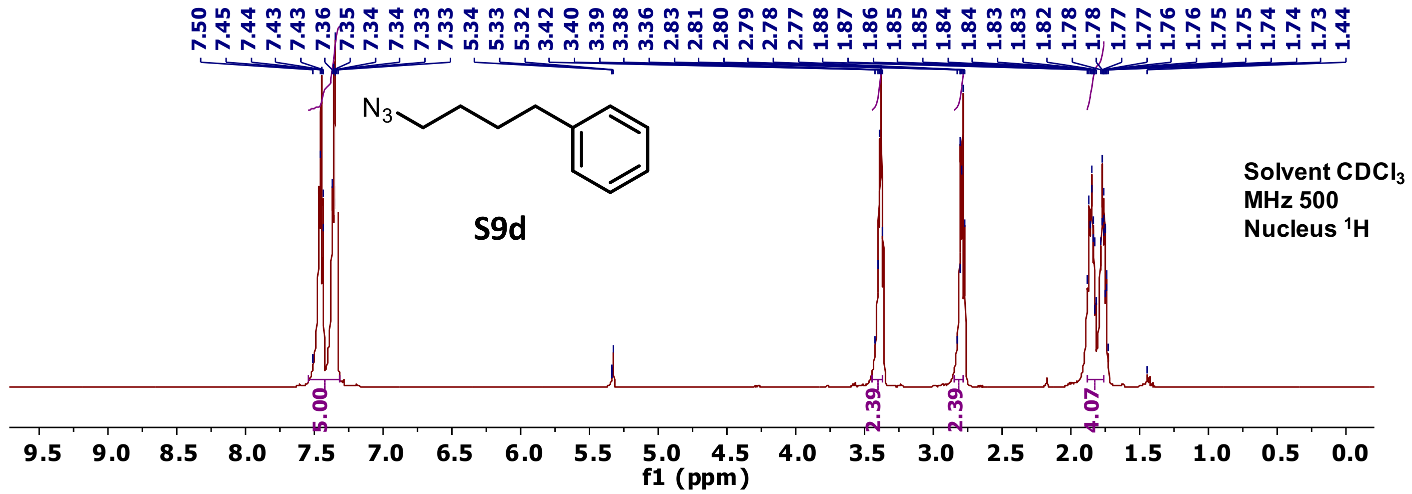
7.50 7.45 7.44 7.43 7.43 7.36 7.35 7.34 7.34 7.33 7.33 5.34 5.33 5.32 3.42 3.40 3.39 3.38 3.36 2.83 2.81 2.80 2.79 2.78 2.77 1.88 1.87 1.86 1.85 1.85 1.84 1.84 1.83 1.83 1.82 1.78 1.78 1.77 1.77 1.76 1.76 1.75 1.75 1.74 1.74 1.73 1.44
N_3
S9d
Solvent $CDCl_3$
MHz 500
Nucleus ^1H
5.00
2.39
2.39
4.07
9.5 9.0 8.5 8.0 7.5 7.0 6.5 6.0 5.5 5.0 4.5 4.0 3.5 3.0 2.5 2.0 1.5 1.0 0.5 0.0
f1 (ppm)

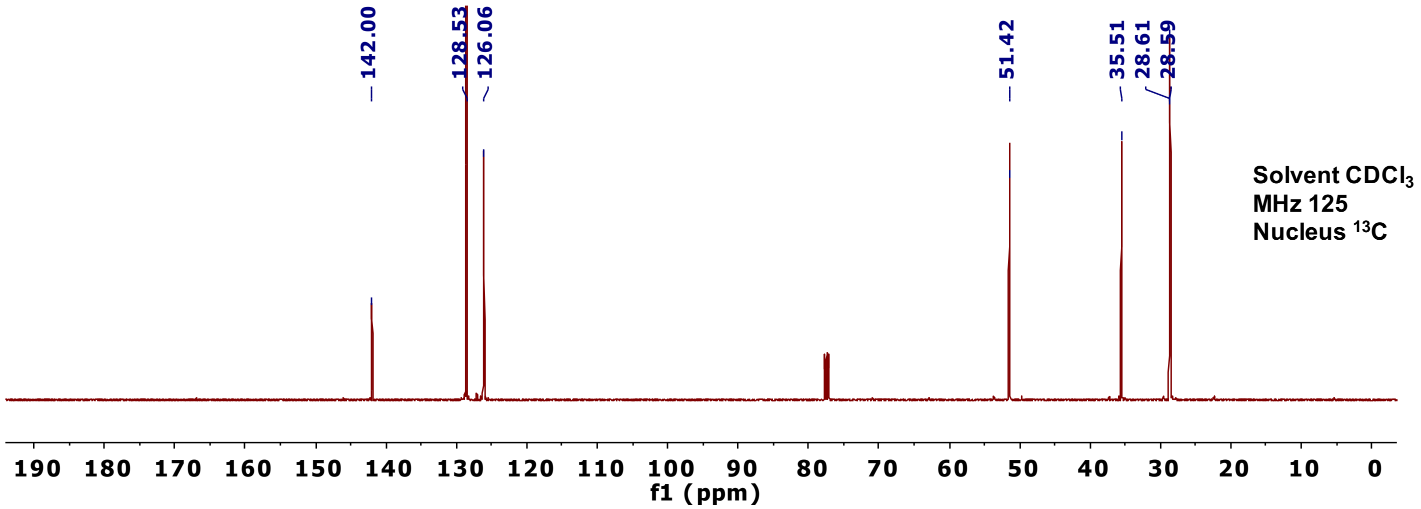
142.00
128.53
126.06
51.42
35.51
28.61
28.59
Solvent $CDCl_3$
MHz 125
Nucleus ^{13}C
190 180 170 160 150 140 130 120 110 100 90 80 70 60 50 40 30 20 10 0
f1 (ppm)

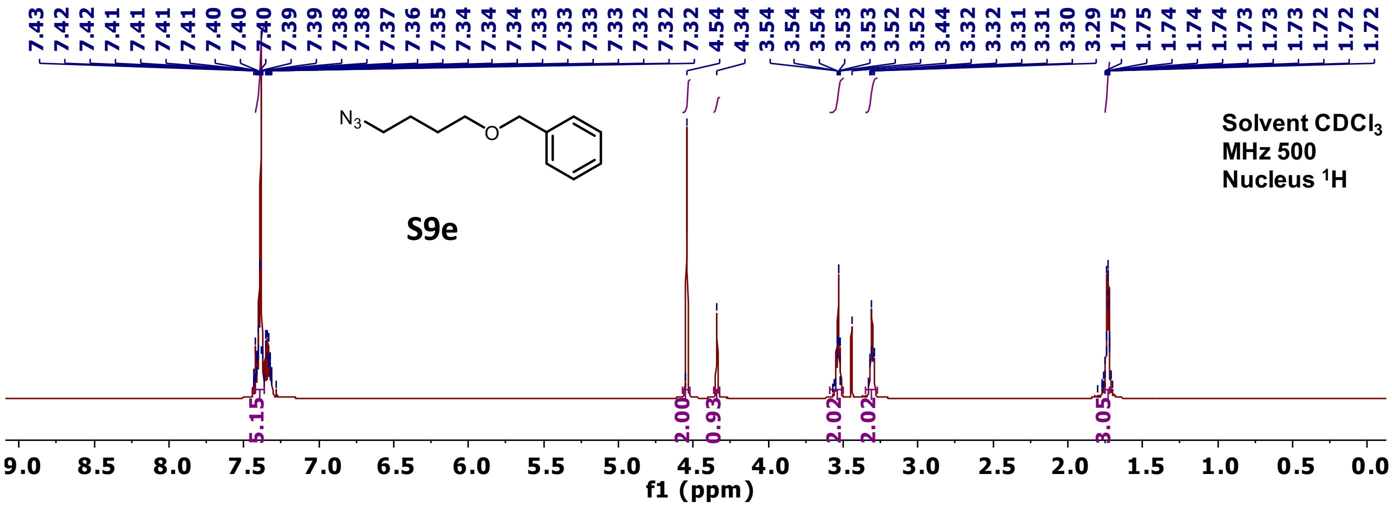
7.43 7.42 7.42 7.41 7.41 7.41 7.41 7.40 7.40 7.40 7.39 7.39 7.38 7.38 7.37 7.36 7.35 7.34 7.34 7.33 7.33 7.33 7.33 7.32 7.32 7.32 4.54 4.34 3.54 3.54 3.54 3.53 3.53 3.52 3.52 3.44 3.32 3.32 3.31 3.31 3.30 3.29 1.75 1.75 1.74 1.74 1.74 1.73 1.73 1.73 1.72 1.72 1.72
N3
O
S9e
Solvent CDCl3
MHz 500
Nucleus 1H
5.15
2.00
0.93
2.02
2.02
3.05
9.0 8.5 8.0 7.5 7.0 6.5 6.0 5.5 5.0 4.5 4.0 3.5 3.0 2.5 2.0 1.5 1.0 0.5 0.0
f1 (ppm)

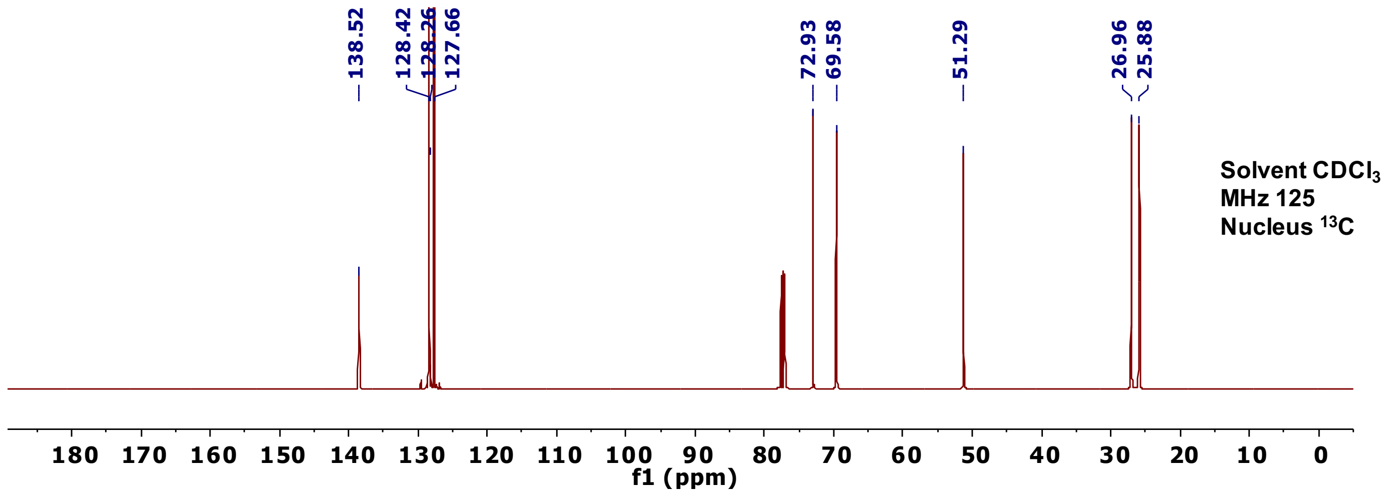
138.52
128.42
128.26
127.66
72.93
69.58
51.29
26.96
25.88
Solvent CDCl3
MHz 125
Nucleus 13C
180 170 160 150 140 130 120 110 100 90 80 70 60 50 40 30 20 10 0
f1 (ppm)

S9f

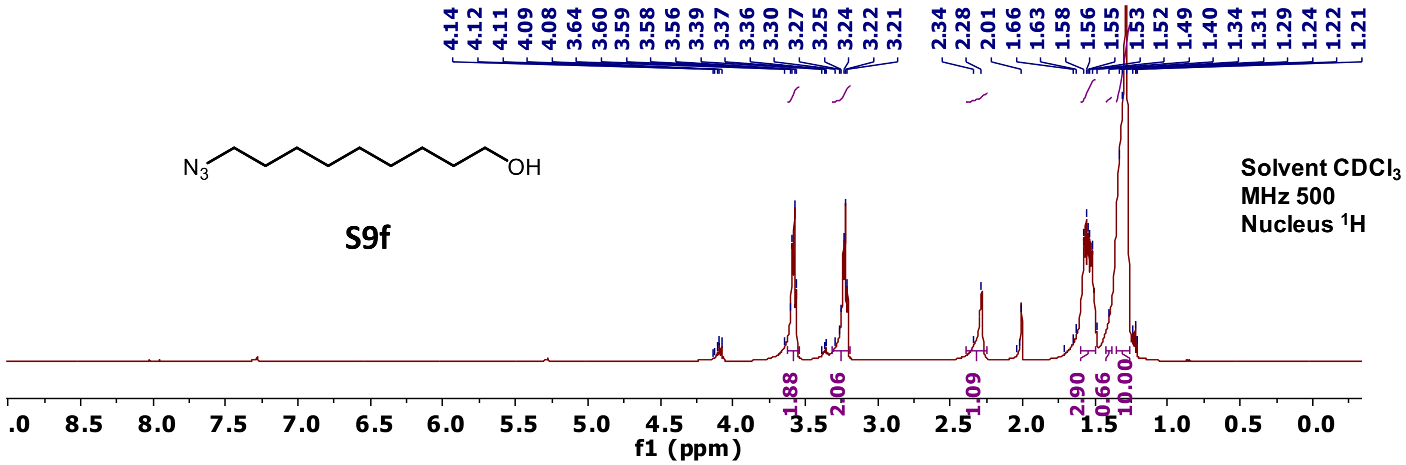
4.14 4.12 4.11 4.09 4.08 3.64 3.60 3.59 3.58 3.56 3.39 3.37 3.36 3.30 3.27 3.25 3.24 3.22 3.21 2.34 2.28 2.01 1.66 1.63 1.58 1.56 1.55 1.53 1.52 1.49 1.40 1.34 1.31 1.29 1.24 1.22 1.21
N3
OH
Solvent CDCl3
MHz 500
Nucleus 1H
1.88
2.06
1.09
2.90
0.66
10.00
.0 8.5 8.0 7.5 7.0 6.5 6.0 5.5 5.0 4.5 4.0 3.5 3.0 2.5 2.0 1.5 1.0 0.5 0.0
f1 (ppm)

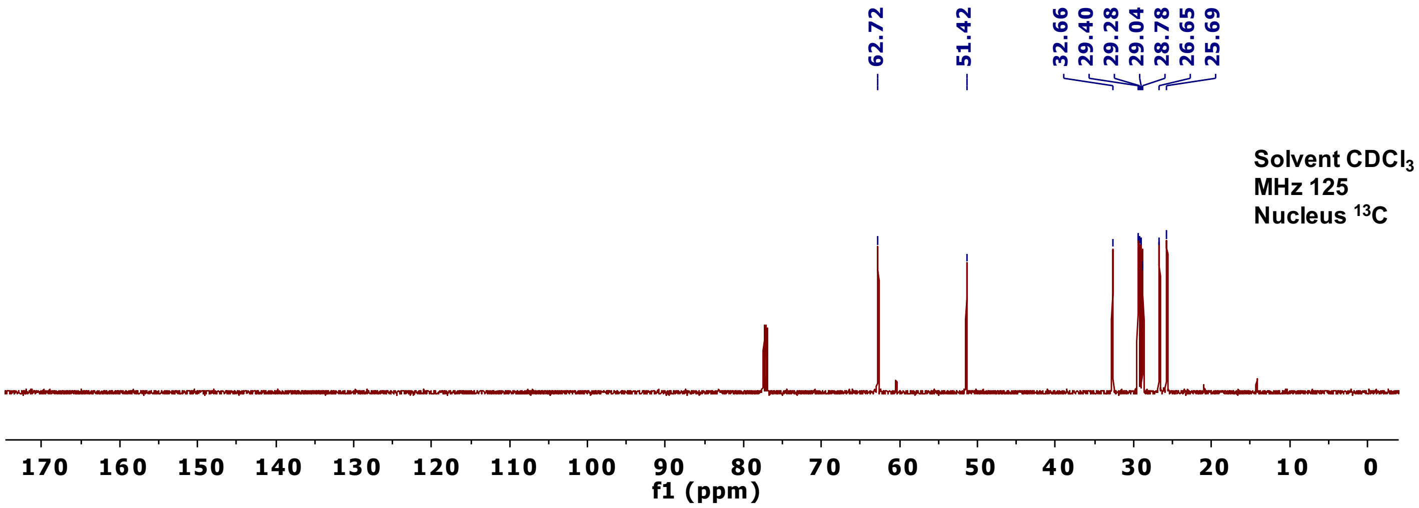
62.72
51.42
32.66 29.40 29.28 29.04 28.78 26.65 25.69
Solvent CDCl3
MHz 125
Nucleus 13C
170 160 150 140 130 120 110 100 90 80 70 60 50 40 30 20 10 0
f1 (ppm)

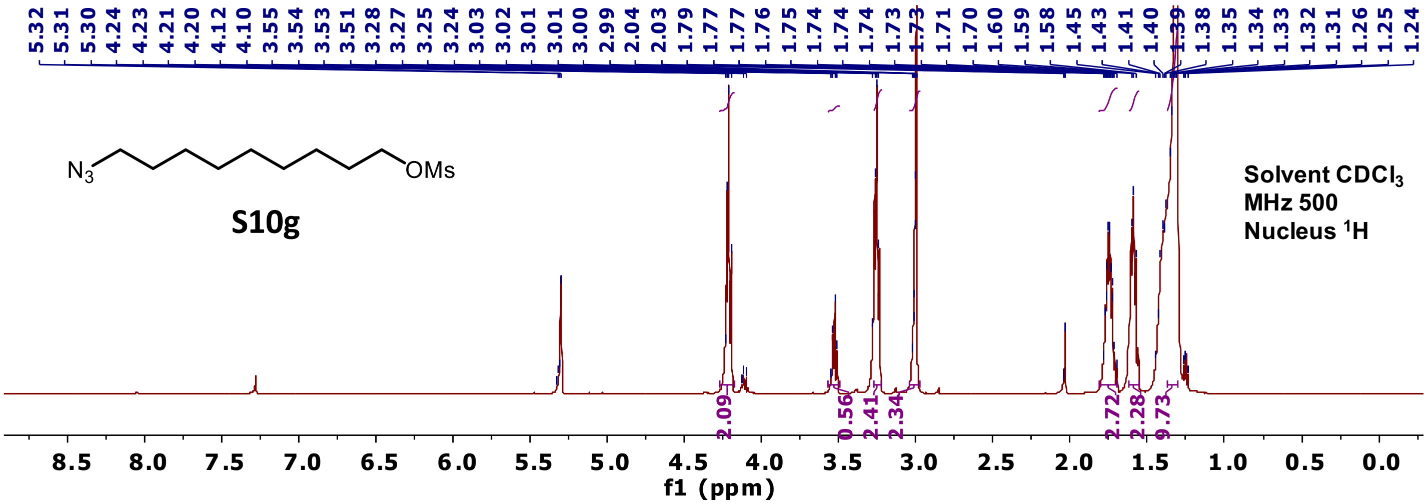
N3
OMs
S10g
Solvent CDCl3
MHz 500
Nucleus 1H
5.32 5.31 5.30 4.24 4.23 4.21 4.20 4.12 4.10 3.55 3.54 3.53 3.51 3.28 3.27 3.25 3.24 3.03 3.02 3.01 3.01 3.00 2.99 2.04 2.03 1.79 1.77 1.77 1.76 1.75 1.74 1.74 1.74 1.73 1.73 1.71 1.70 1.60 1.59 1.58 1.45 1.43 1.41 1.40 1.39 1.38 1.35 1.34 1.33 1.32 1.31 1.26 1.25 1.24
2.09
0.56
2.41
2.34
2.72
2.28
9.73
8.5 8.0 7.5 7.0 6.5 6.0 5.5 5.0 4.5 4.0 3.5 3.0 2.5 2.0 1.5 1.0 0.5 0.0
f1 (ppm)
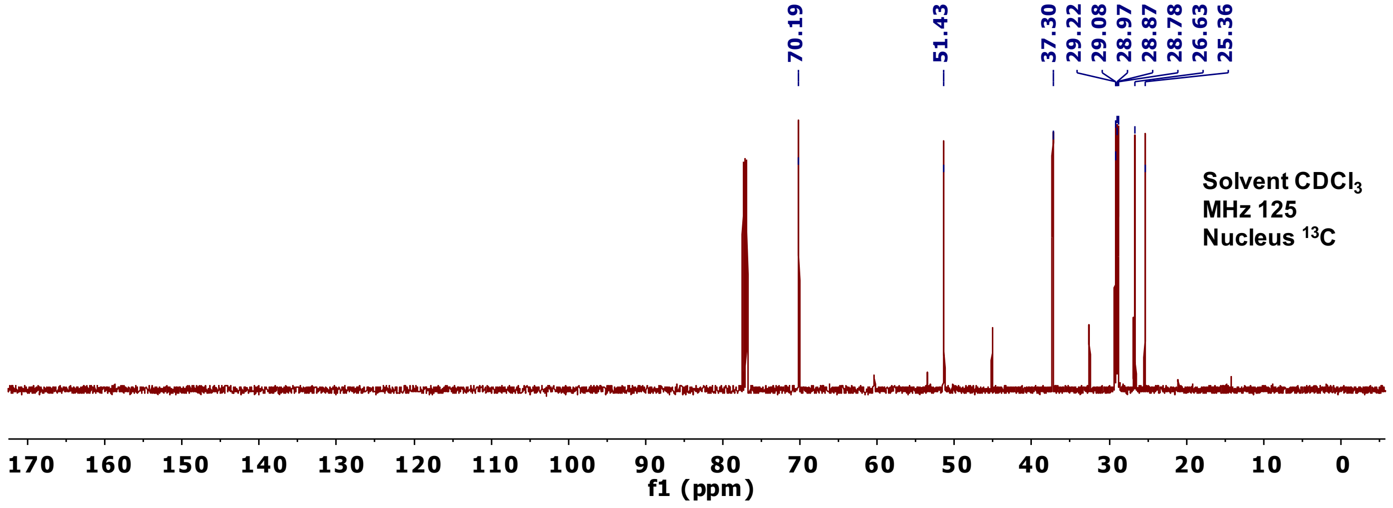
70.19
51.43
37.30 29.22 29.08 28.97 28.87 28.78 26.63 25.36
Solvent CDCl3
MHz 125
Nucleus 13C
170 160 150 140 130 120 110 100 90 80 70 60 50 40 30 20 10 0
f1 (ppm)

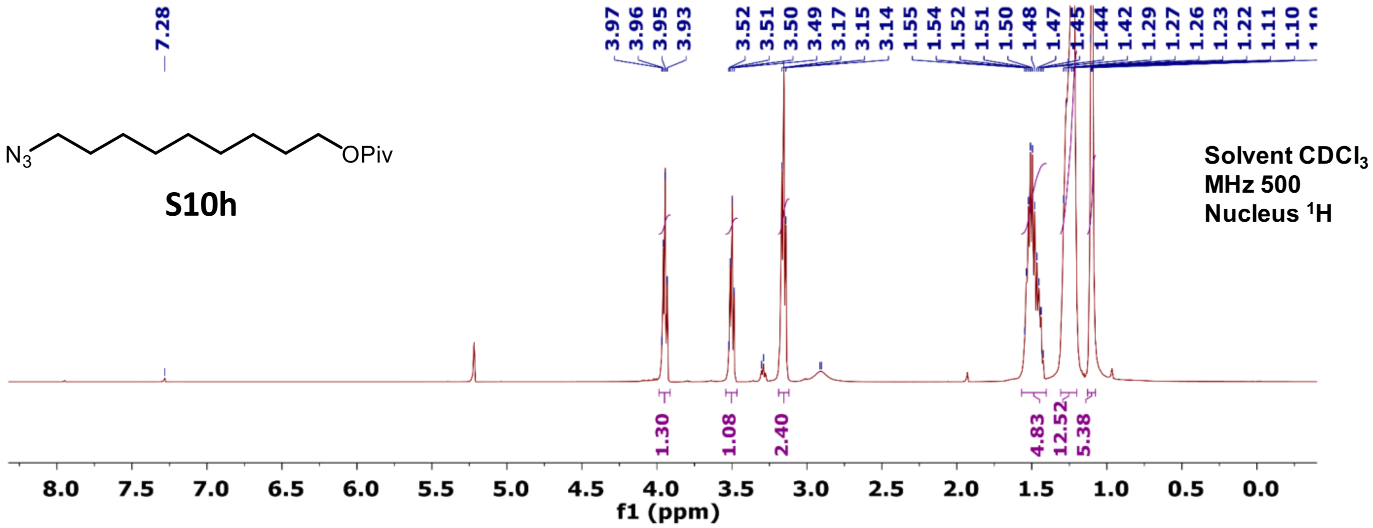

N3
OPiv
S10h
Solvent CDCl3
MHz 500
Nucleus 1H
7.28
3.97
3.96
3.95
3.93
3.52
3.51
3.50
3.49
3.17
3.15
3.14
1.55
1.54
1.52
1.51
1.50
1.48
1.47
1.45
1.44
1.42
1.29
1.27
1.26
1.23
1.22
1.11
1.10
1.30
1.08
2.40
4.83
12.52
5.38
8.0 7.5 7.0 6.5 6.0 5.5 5.0 4.5 4.0 3.5 3.0 2.5 2.0 1.5 1.0 0.5 0.0
f1 (ppm)

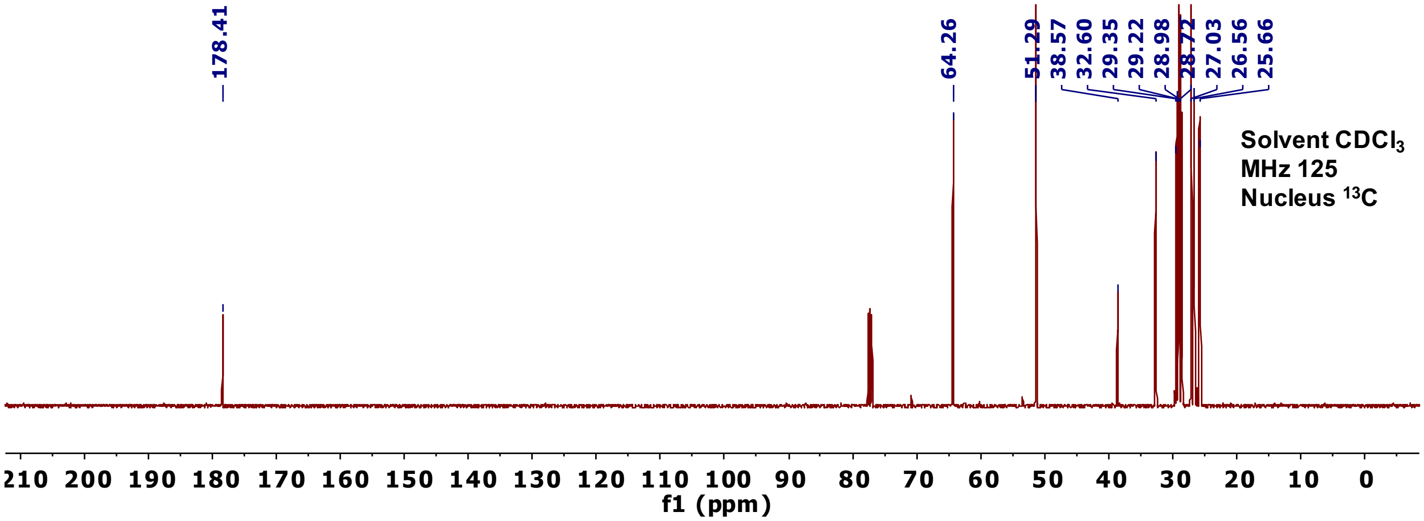

178.41
64.26
51.29
38.57
32.60
29.35
29.22
28.98
28.72
27.03
26.56
25.66
Solvent CDCl3
MHz 125
Nucleus 13C
210 200 190 180 170 160 150 140 130 120 110 100 90 80 70 60 50 40 30 20 10 0
f1 (ppm)

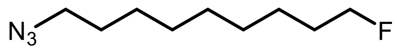

S10i

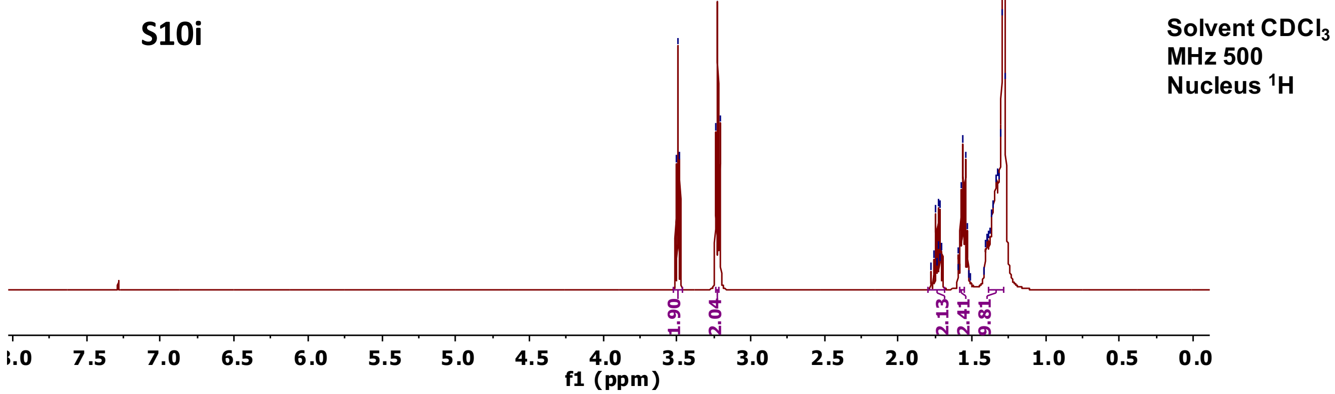

3.51 3.49 3.48 3.24 3.22 3.21 1.78 1.76 1.74 1.74 1.73 1.72 1.72 1.71 1.70 1.59 1.59 1.57 1.56 1.54 1.53 1.52 1.51 1.42 1.41 1.40 1.39 1.38 1.36 1.36 1.34 1.33 1.32 1.30 1.29 1.28 1.27
1.90
2.04
2.13
2.41
9.81
Solvent CDCl3
MHz 500
Nucleus 1H
f1 (ppm)

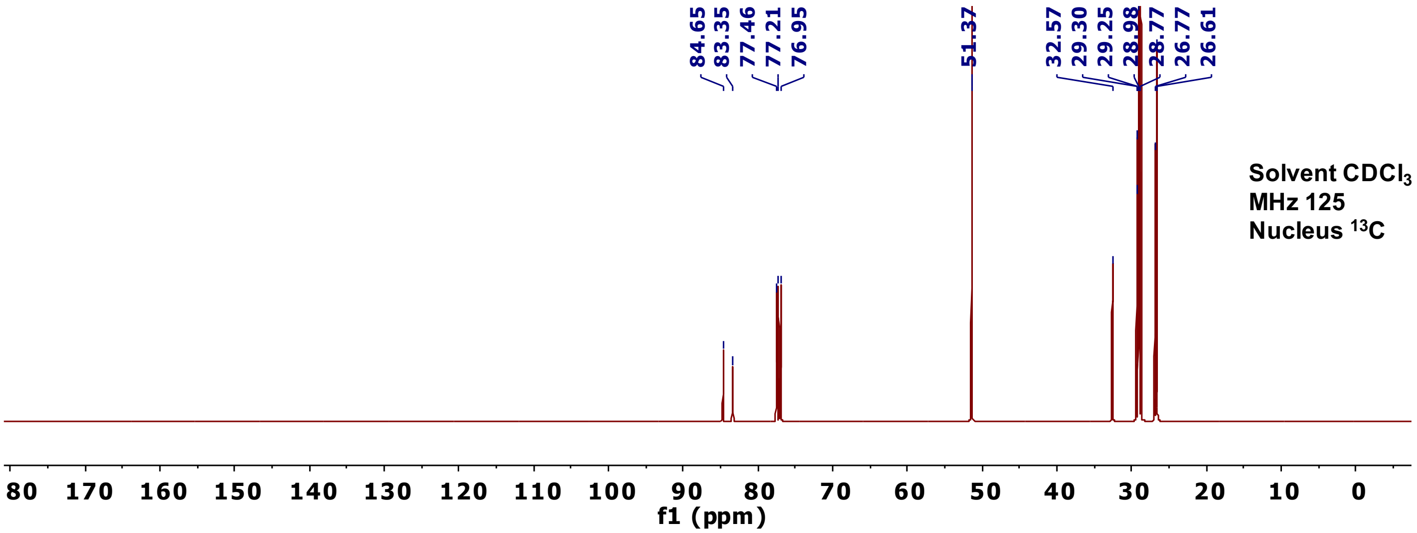

84.65 83.35 77.46 77.21 76.95
51.37
32.57 29.30 29.25 28.98 28.77 26.77 26.61
Solvent CDCl3
MHz 125
Nucleus 13C
f1 (ppm)

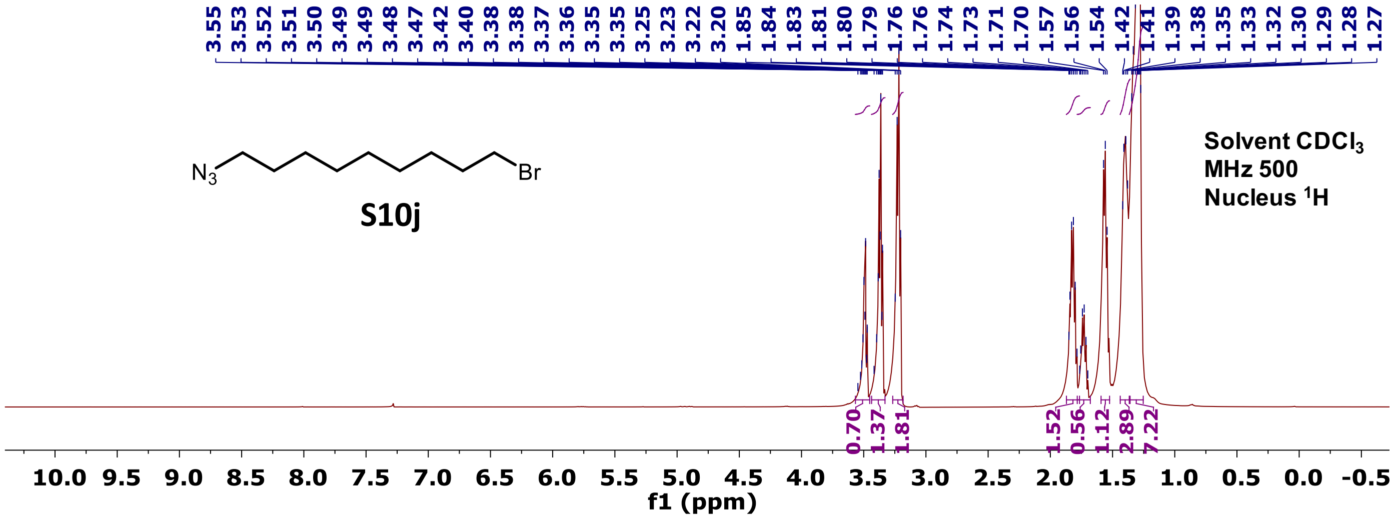

3.55 3.53 3.52 3.51 3.50 3.49 3.49 3.48 3.47 3.42 3.40 3.38 3.38 3.37 3.36 3.35 3.35 3.25 3.23 3.22 3.20 1.85 1.84 1.83 1.81 1.80 1.79 1.76 1.76 1.74 1.73 1.71 1.70 1.57 1.56 1.54 1.42 1.41 1.39 1.38 1.35 1.33 1.32 1.30 1.29 1.28 1.27
N_3
Br
S10j
Solvent $CDCl_3$
MHz 500
Nucleus ^1H
0.70 1.37 1.81 1.52 0.56 1.12 2.89 7.22
10.0 9.5 9.0 8.5 8.0 7.5 7.0 6.5 6.0 5.5 5.0 4.5 4.0 3.5 3.0 2.5 2.0 1.5 1.0 0.5 0.0 -0.5
f1 (ppm)

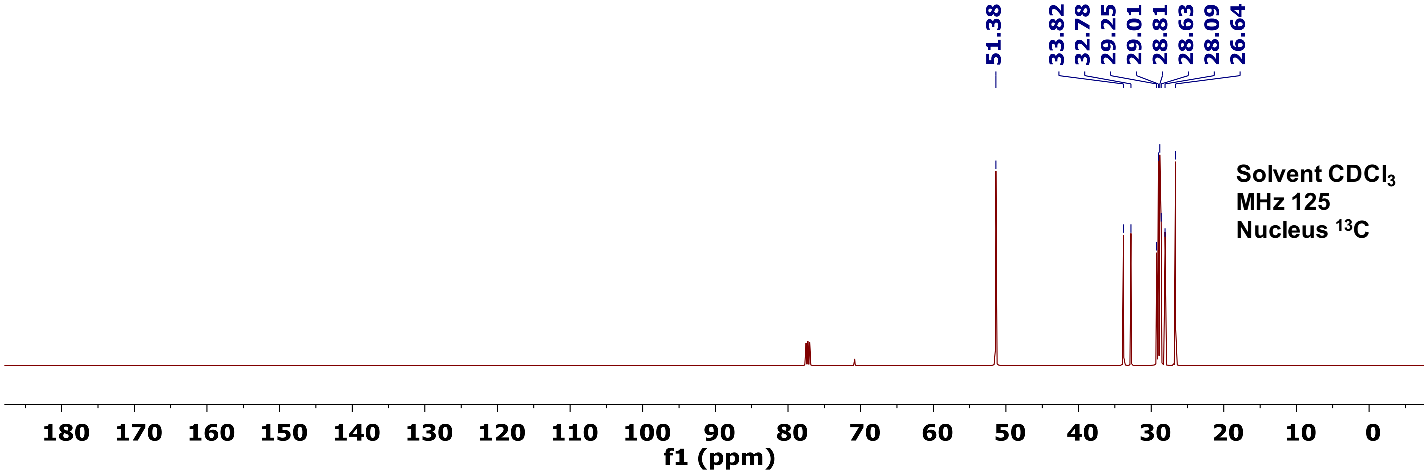

51.38
33.82 32.78 29.25 29.01 28.81 28.63 28.09 26.64
Solvent $CDCl_3$
MHz 125
Nucleus ^{13}C
180 170 160 150 140 130 120 110 100 90 80 70 60 50 40 30 20 10 0
f1 (ppm)

S10k

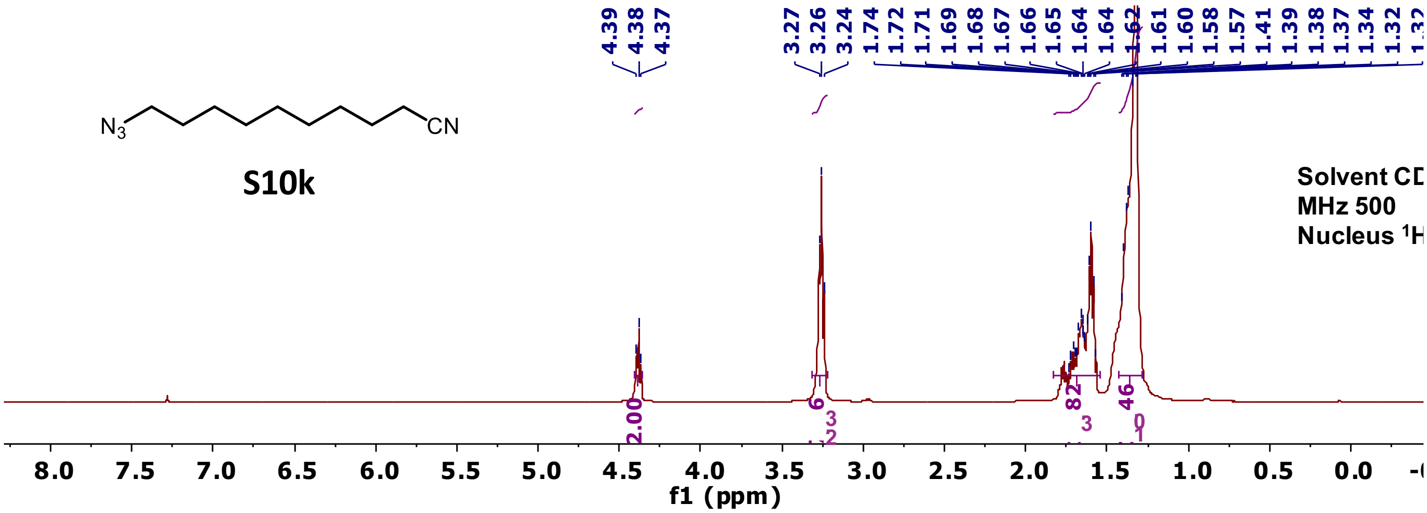
N3
CN
4.39
4.38
4.37
3.27
3.26
3.24
1.74
1.72
1.71
1.69
1.68
1.67
1.66
1.65
1.64
1.64
1.62
1.61
1.60
1.58
1.57
1.41
1.39
1.38
1.37
1.34
1.32
Solvent CD
MHz 500
Nucleus 1H
2.00
8.0 7.5 7.0 6.5 6.0 5.5 5.0 4.5 4.0 3.5 3.0 2.5 2.0 1.5 1.0 0.5 0.0
f1 (ppm)

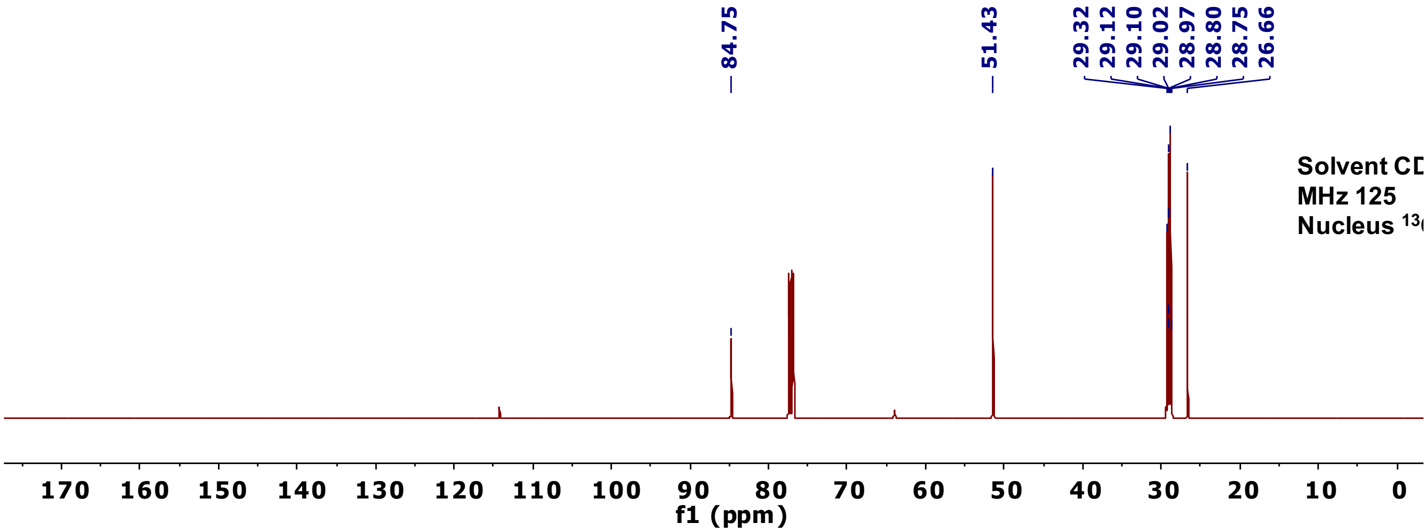
84.75
51.43
29.32
29.12
29.10
29.02
28.97
28.80
28.75
26.66
Solvent CD
MHz 125
Nucleus 13
170 160 150 140 130 120 110 100 90 80 70 60 50 40 30 20 10 0
f1 (ppm)

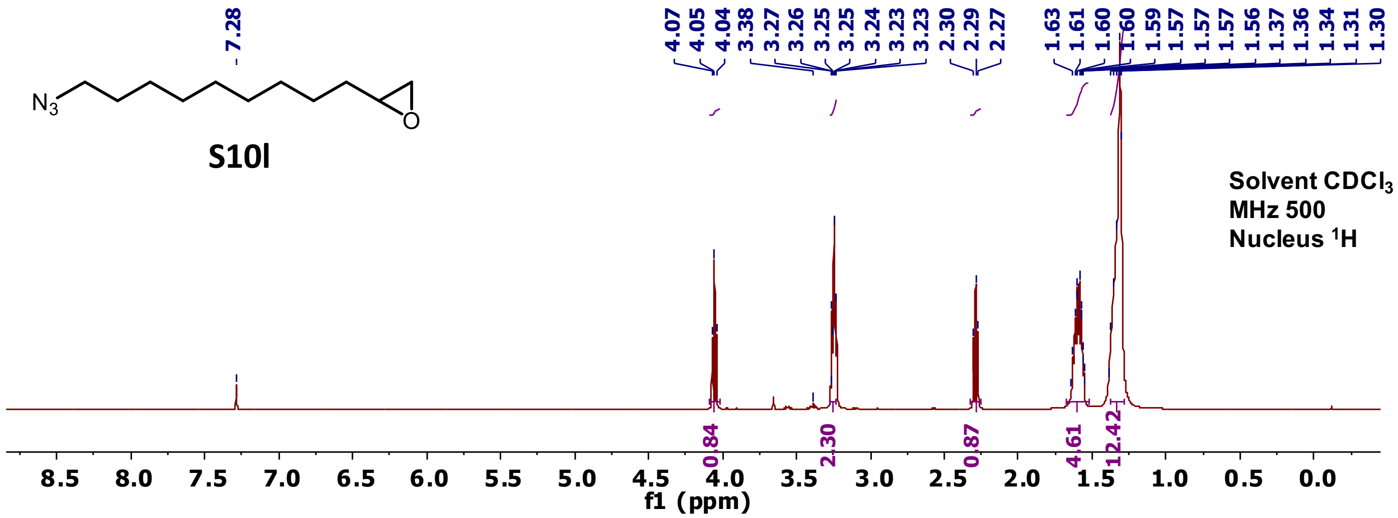
S10l
N3
O
7.28
4.07 4.05 4.04 3.38 3.27 3.26 3.25 3.25 3.24 3.23 3.23 2.30 2.29 2.27
1.63 1.61 1.60 1.60 1.59 1.57 1.57 1.57 1.56 1.37 1.36 1.34 1.31 1.30
Solvent CDCl3
MHz 500
Nucleus 1H
0.84
2.30
0.87
4.61
12.42
8.5 8.0 7.5 7.0 6.5 6.0 5.5 5.0 4.5 4.0 3.5 3.0 2.5 2.0 1.5 1.0 0.5 0.0
f1 (ppm)

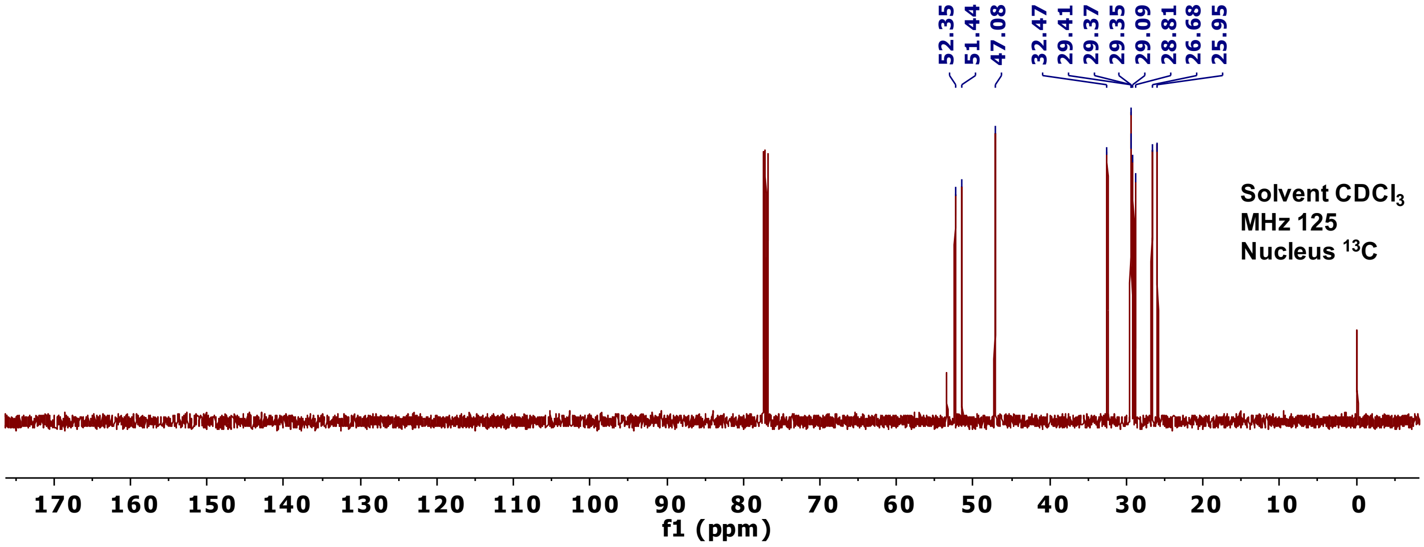
52.35 51.44 47.08
32.47 29.41 29.37 29.35 29.09 28.81 26.68 25.95
Solvent CDCl3
MHz 125
Nucleus 13C
170 160 150 140 130 120 110 100 90 80 70 60 50 40 30 20 10 0
f1 (ppm)

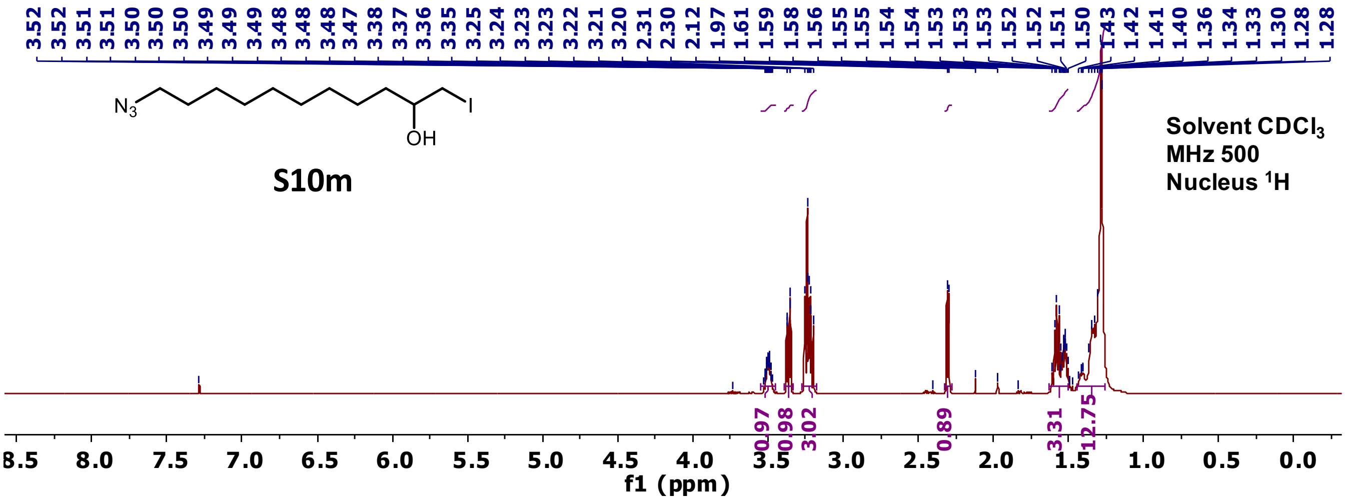
3.52 3.52 3.51 3.51 3.50 3.50 3.50 3.49 3.49 3.49 3.48 3.48 3.48 3.47 3.38 3.37 3.36 3.35 3.25 3.24 3.23 3.23 3.22 3.21 3.20 2.31 2.30 2.12 1.97 1.61 1.59 1.58 1.56 1.55 1.55 1.54 1.54 1.53 1.53 1.53 1.52 1.52 1.51 1.50 1.43 1.42 1.41 1.40 1.36 1.34 1.33 1.30 1.28 1.28
N_3
OH
I
S10m
Solvent $CDCl_3$
MHz 500
Nucleus ^1H
0.97 0.98 3.02 0.89 3.31 12.75
8.5 8.0 7.5 7.0 6.5 6.0 5.5 5.0 4.5 4.0 3.5 3.0 2.5 2.0 1.5 1.0 0.5 0.0
f1 (ppm)

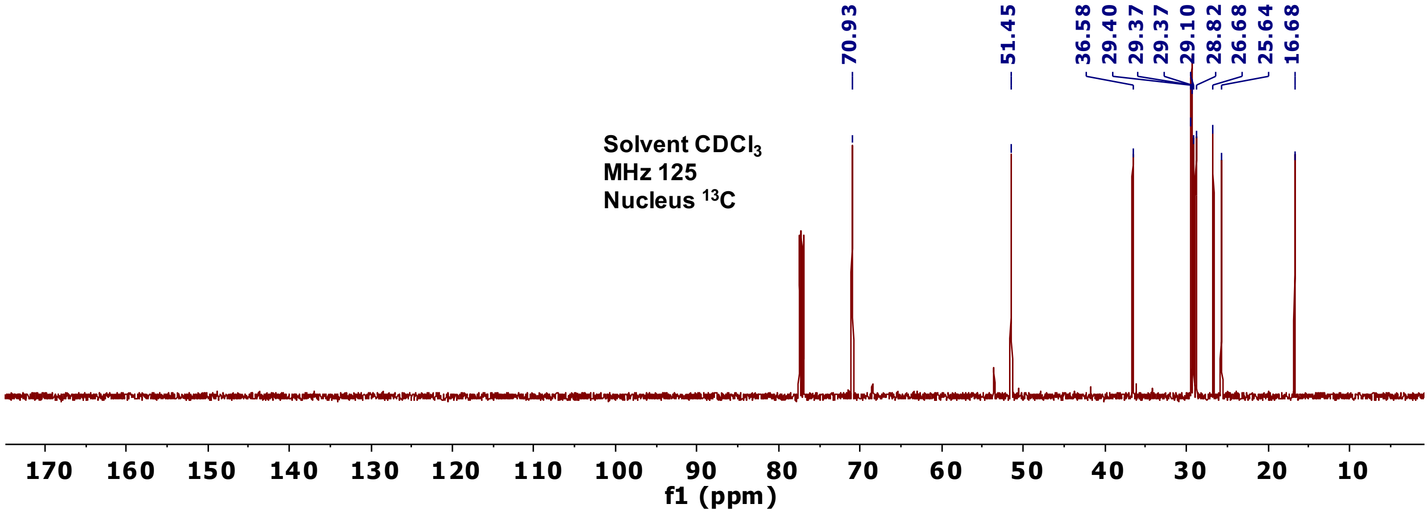
70.93
51.45
36.58 29.40 29.37 29.37 29.10 28.82 26.68 25.64 16.68
Solvent $CDCl_3$
MHz 125
Nucleus ^{13}C
170 160 150 140 130 120 110 100 90 80 70 60 50 40 30 20 10
f1 (ppm)

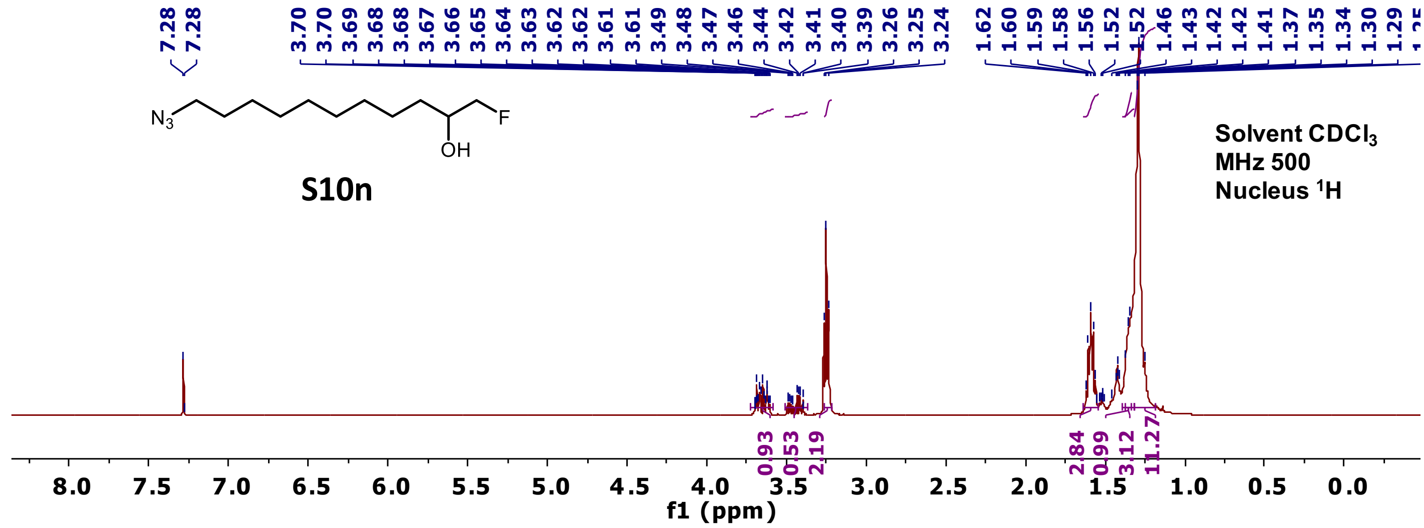
7.28
7.28
3.70
3.70
3.69
3.68
3.68
3.67
3.66
3.65
3.64
3.63
3.62
3.62
3.61
3.61
3.49
3.48
3.47
3.46
3.44
3.42
3.41
3.40
3.39
3.26
3.25
3.24
1.62
1.60
1.59
1.58
1.56
1.52
1.52
1.46
1.43
1.42
1.42
1.41
1.37
1.35
1.34
1.30
1.29
N_3
OH
F
S10n
Solvent $CDCl_3$
MHz 500
Nucleus ^1H
0.93
0.53
2.19
2.84
0.99
3.12
11.27
8.0 7.5 7.0 6.5 6.0 5.5 5.0 4.5 4.0 3.5 3.0 2.5 2.0 1.5 1.0 0.5 0.0
f1 (ppm)

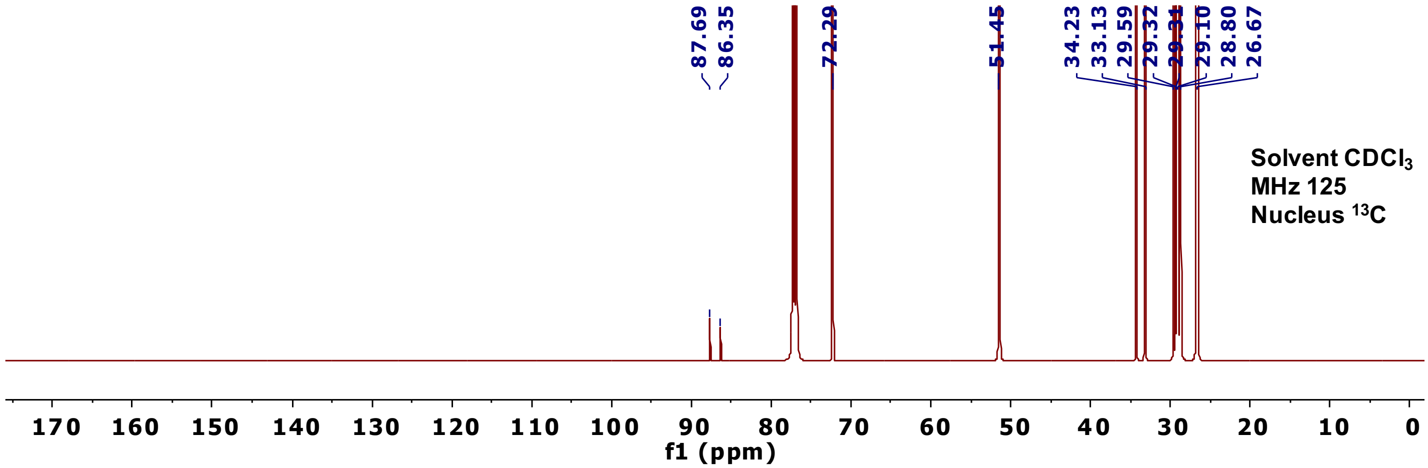
87.69
86.35
72.29
51.45
34.23
33.13
29.59
29.32
29.31
29.10
28.80
26.67
Solvent $CDCl_3$
MHz 125
Nucleus ^{13}C
170 160 150 140 130 120 110 100 90 80 70 60 50 40 30 20 10 0
f1 (ppm)

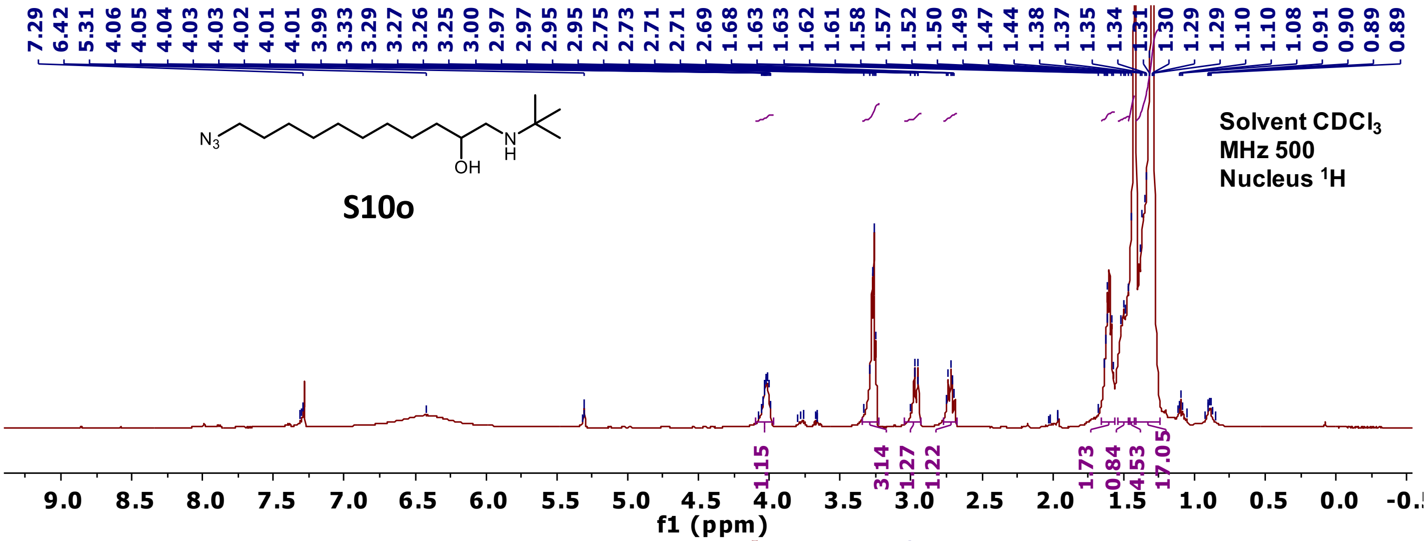
7.29 6.42 5.31 4.06 4.05 4.04 4.03 4.03 4.02 4.01 4.01 3.99 3.33 3.29 3.27 3.26 3.25 3.00 2.97 2.97 2.95 2.95 2.75 2.73 2.71 2.71 2.69 1.68 1.63 1.63 1.62 1.61 1.58 1.57 1.52 1.50 1.49 1.47 1.44 1.38 1.37 1.35 1.34 1.31 1.30 1.29 1.29 1.10 1.10 1.08 0.91 0.90 0.89 0.89
N3
OH
N
H
S10o
Solvent CDCl3
MHz 500
Nucleus 1H
1.15 3.14 1.27 1.22 1.73 0.84 4.53 17.05
9.0 8.5 8.0 7.5 7.0 6.5 6.0 5.5 5.0 4.5 4.0 3.5 3.0 2.5 2.0 1.5 1.0 0.5 0.0
f1 (ppm)

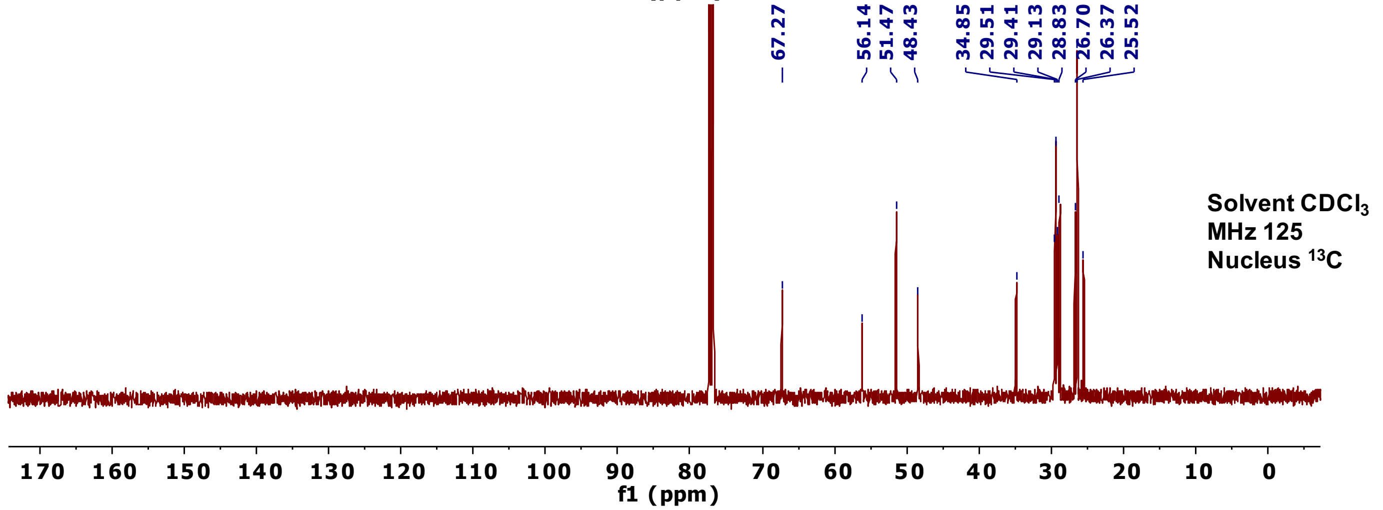
67.27
56.14 51.47 48.43
34.85 29.51 29.41 29.13 28.83 26.70 26.37 25.52
Solvent CDCl3
MHz 125
Nucleus 13C
170 160 150 140 130 120 110 100 90 80 70 60 50 40 30 20 10 0
f1 (ppm)

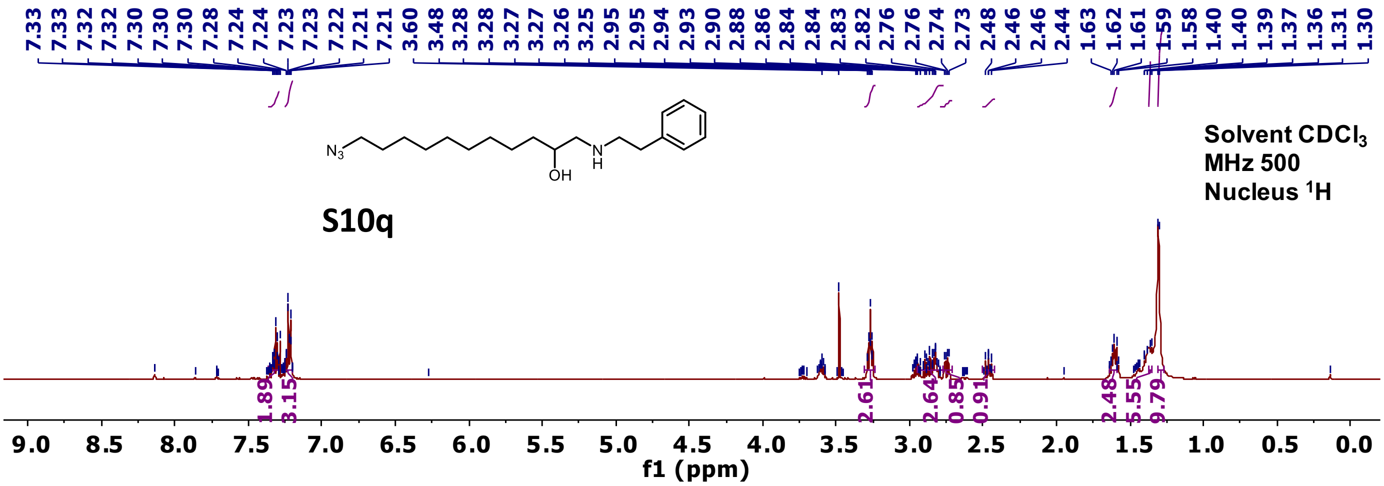
N3
OH
N
H
S10q
Solvent CDCl3
MHz 500
Nucleus 1H
f1 (ppm)

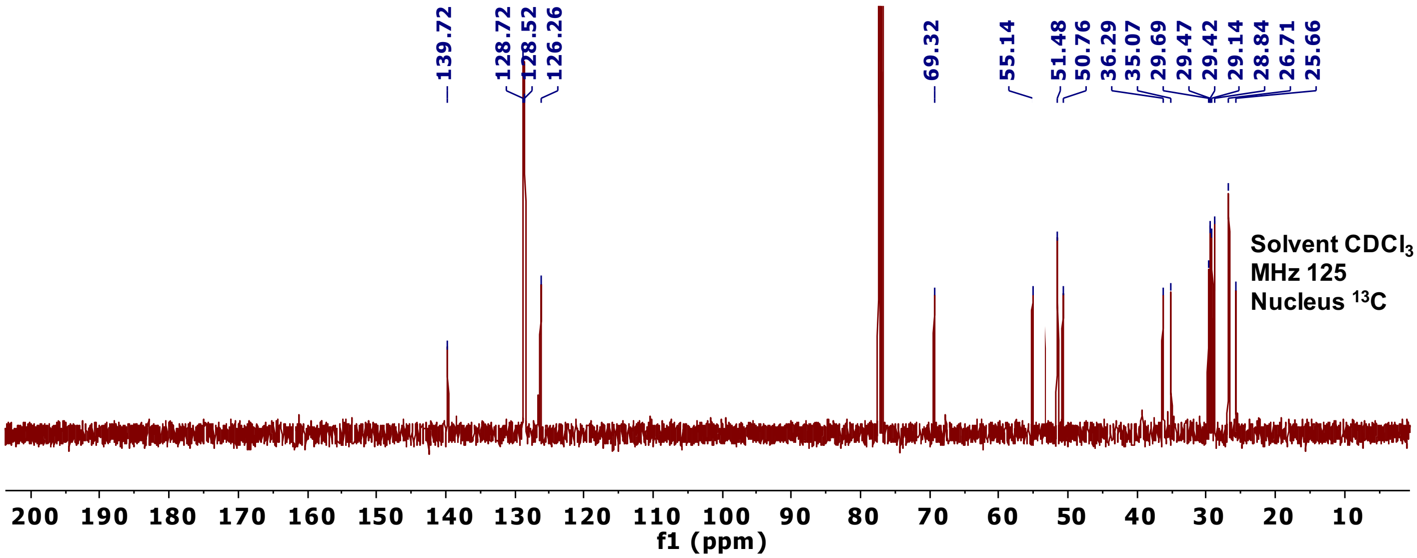
139.72
128.72
128.52
126.26
69.32
55.14
51.48
50.76
36.29
35.07
29.69
29.47
29.42
29.14
28.84
26.71
25.66
Solvent CDCl3
MHz 125
Nucleus 13C
f1 (ppm)

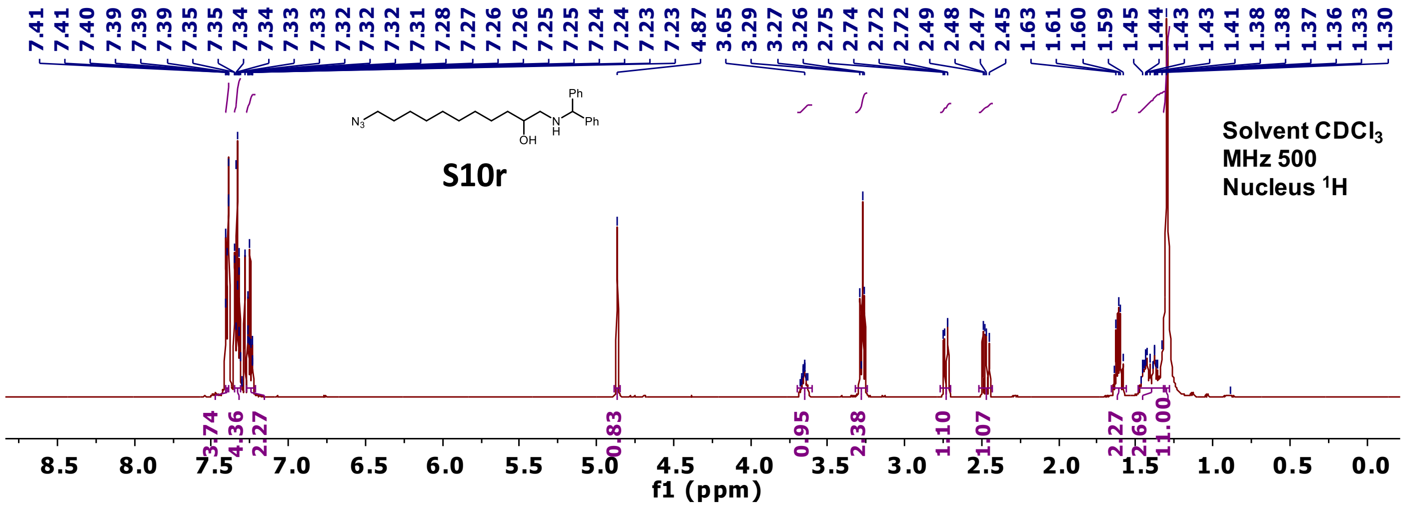

7.41 7.41 7.40 7.39 7.39 7.39 7.35 7.35 7.34 7.34 7.33 7.33 7.32 7.32 7.32 7.31 7.28 7.27 7.26 7.26 7.25 7.25 7.24 7.24 7.23 7.23 4.87 3.65 3.29 3.27 3.26 2.75 2.74 2.72 2.72 2.49 2.48 2.47 2.45 1.63 1.61 1.60 1.59 1.45 1.44 1.43 1.43 1.41 1.38 1.38 1.37 1.36 1.33 1.30
N3
OH
N
H
Ph
Ph
S10r
Solvent $CDCl_3$
MHz 500
Nucleus ^1H
3.74 4.36 2.27 0.83 0.95 2.38 1.10 1.07 2.27 2.69 11.00
8.5 8.0 7.5 7.0 6.5 6.0 5.5 5.0 4.5 4.0 3.5 3.0 2.5 2.0 1.5 1.0 0.5 0.0
f1 (ppm)

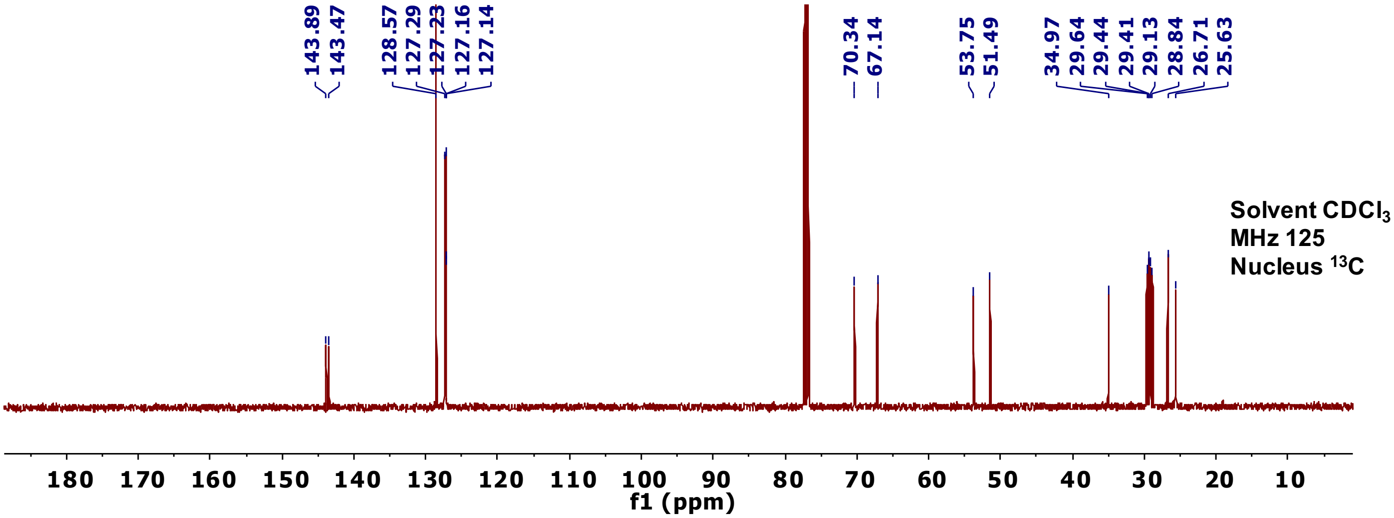

143.89 143.47 128.57 127.29 127.23 127.16 127.14 70.34 67.14 53.75 51.49 34.97 29.64 29.44 29.41 29.13 28.84 26.71 25.63
Solvent $CDCl_3$
MHz 125
Nucleus ^{13}C
180 170 160 150 140 130 120 110 100 90 80 70 60 50 40 30 20 10
f1 (ppm)

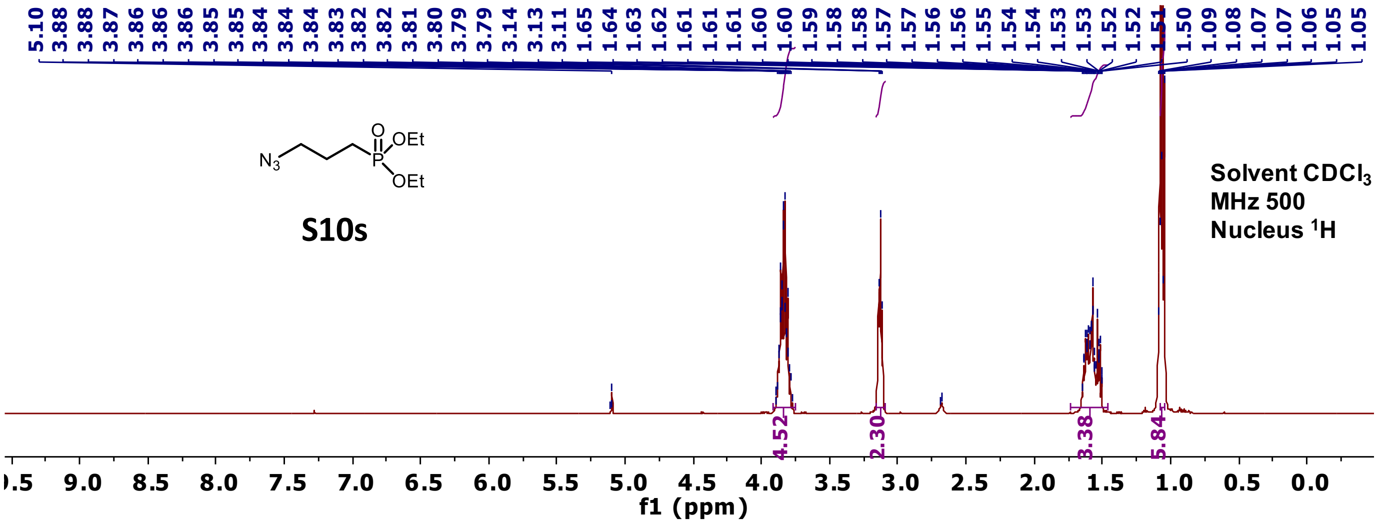
5.10 3.88 3.88 3.87 3.86 3.86 3.86 3.85 3.85 3.84 3.84 3.84 3.83 3.82 3.82 3.81 3.80 3.79 3.14 3.13 3.11 1.65 1.64 1.63 1.62 1.61 1.61 1.61 1.60 1.60 1.59 1.58 1.58 1.57 1.57 1.56 1.56 1.55 1.54 1.54 1.53 1.53 1.52 1.52 1.51 1.50 1.09 1.08 1.07 1.07 1.06 1.05 1.05
O
OEt
N3
P
OEt
S10s
Solvent CDCl3
MHz 500
Nucleus 1H
4.52
2.30
3.38
5.84
f1 (ppm)

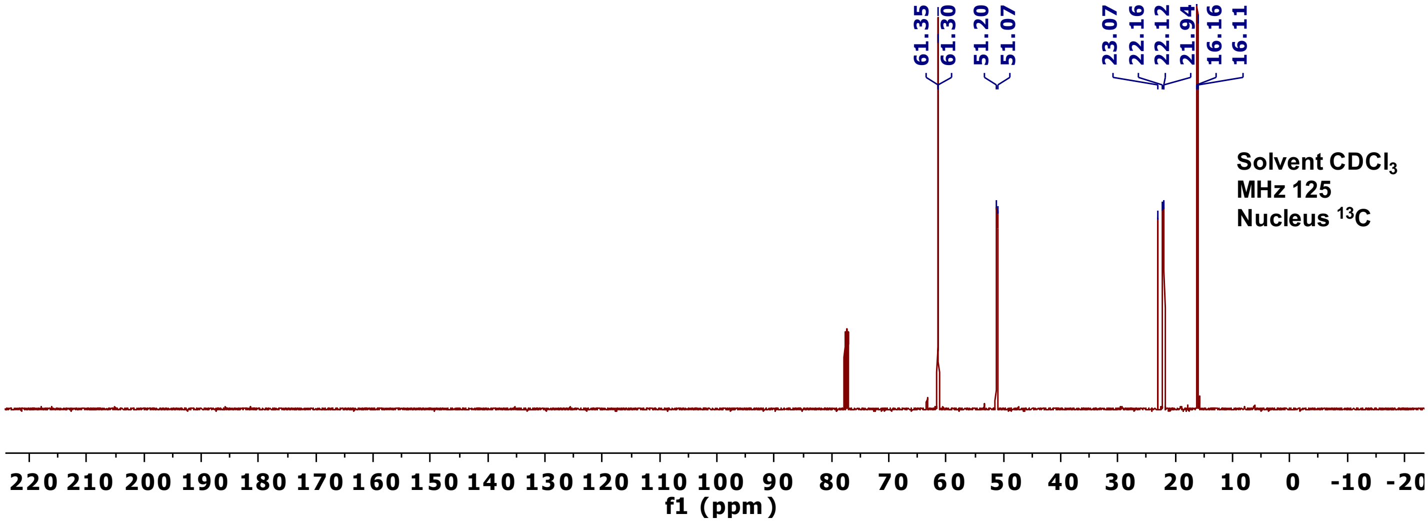
61.35 61.30 51.20 51.07 23.07 22.16 22.12 21.94 16.16 16.11
Solvent CDCl3
MHz 125
Nucleus 13C
f1 (ppm)

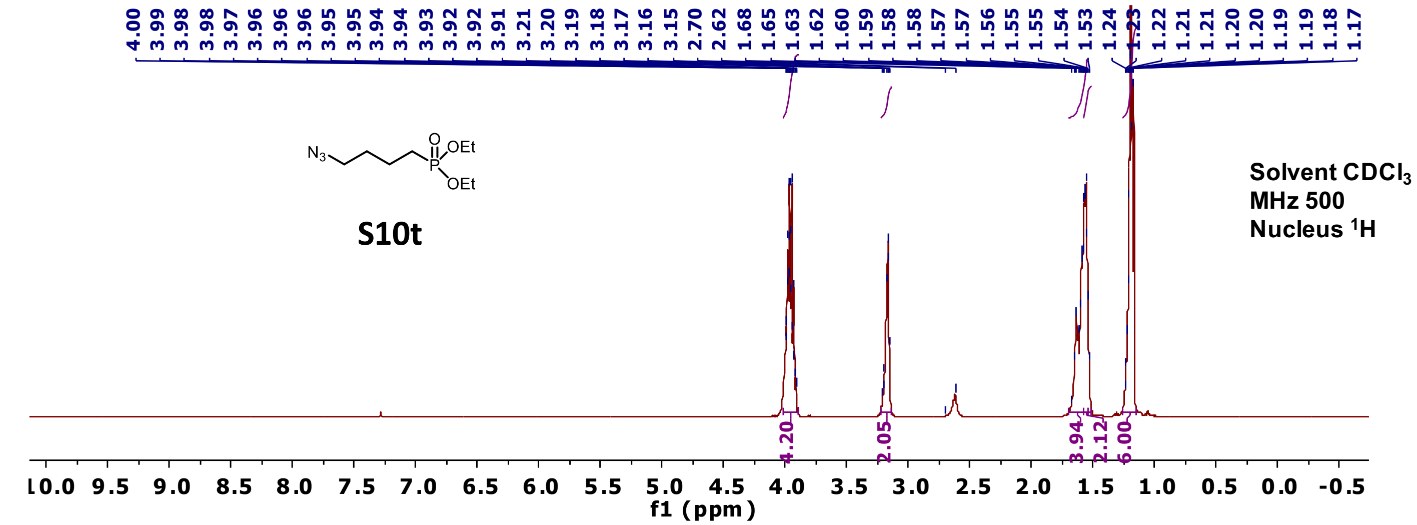
4.00 3.99 3.98 3.98 3.97 3.96 3.96 3.96 3.95 3.95 3.94 3.94 3.93 3.92 3.92 3.91 3.21 3.20 3.19 3.18 3.17 3.16 3.15 2.70 2.62 1.68 1.65 1.63 1.62 1.60 1.59 1.58 1.58 1.57 1.57 1.56 1.55 1.55 1.54 1.53 1.24 1.23 1.22 1.21 1.21 1.20 1.20 1.19 1.19 1.18 1.17
N_3
O
OEt
P
OEt
S10t
Solvent $CDCl_3$
MHz 500
Nucleus ^1H
4.20
2.05
3.94
2.12
6.00
10.0 9.5 9.0 8.5 8.0 7.5 7.0 6.5 6.0 5.5 5.0 4.5 4.0 3.5 3.0 2.5 2.0 1.5 1.0 0.5 0.0 -0.5
f1 (ppm)

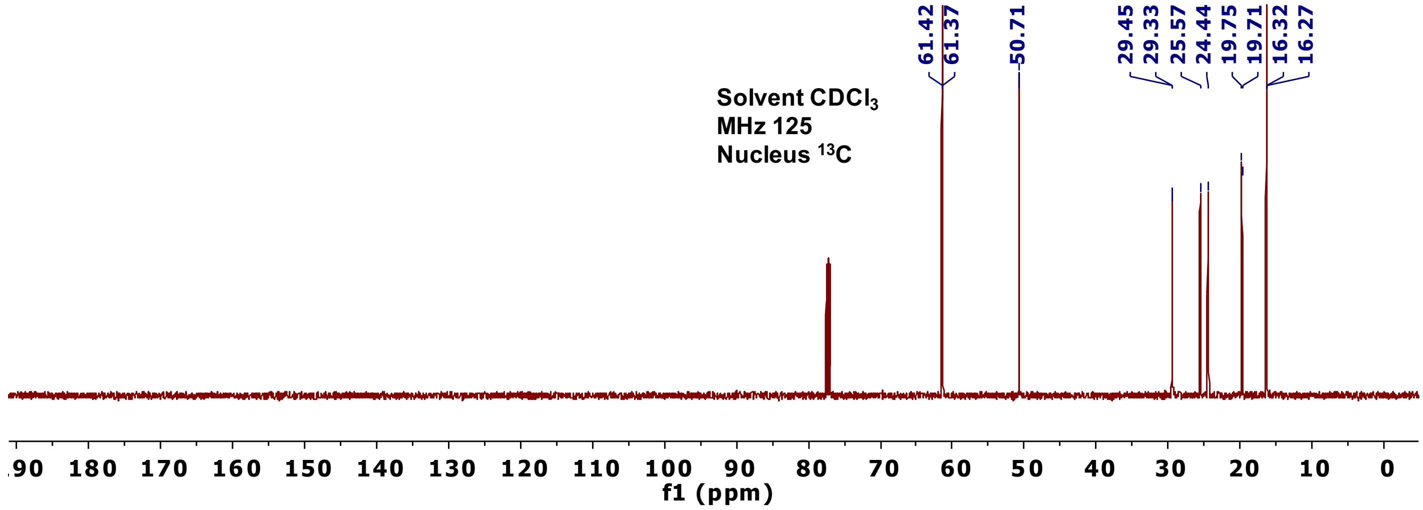
61.42 61.37 50.71 29.45 29.33 25.57 24.44 19.75 19.71 16.32 16.27
Solvent $CDCl_3$
MHz 125
Nucleus ^{13}C
190 180 170 160 150 140 130 120 110 100 90 80 70 60 50 40 30 20 10 0
f1 (ppm)

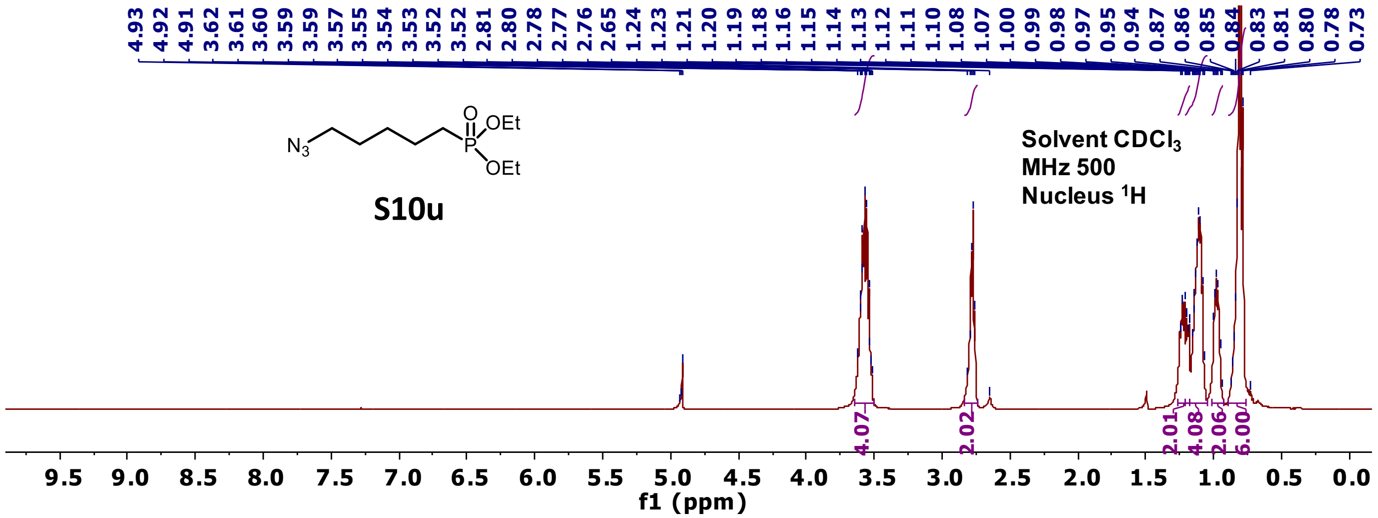
4.93
4.92
4.91
3.62
3.61
3.60
3.59
3.59
3.57
3.55
3.54
3.53
3.52
2.81
2.80
2.78
2.77
2.76
2.65
1.24
1.23
1.21
1.20
1.19
1.18
1.16
1.15
1.14
1.13
1.12
1.11
1.10
1.08
1.07
1.00
0.99
0.98
0.97
0.95
0.94
0.87
0.86
0.85
0.84
0.83
0.81
0.80
0.78
0.73
S10u
Solvent CDCl3
MHz 500
Nucleus 1H
4.07
2.02
2.01
4.08
2.06
6.00
f1 (ppm)

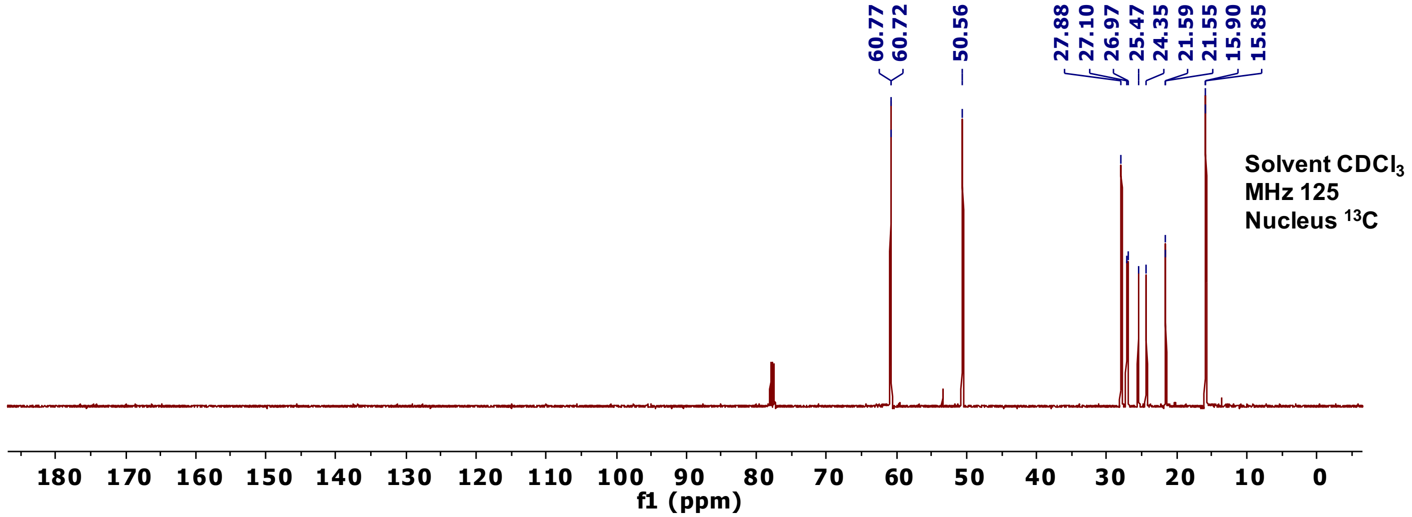
60.77
60.72
50.56
27.88
27.10
26.97
25.47
24.35
21.59
21.55
15.90
15.85
Solvent CDCl3
MHz 125
Nucleus 13C
f1 (ppm)

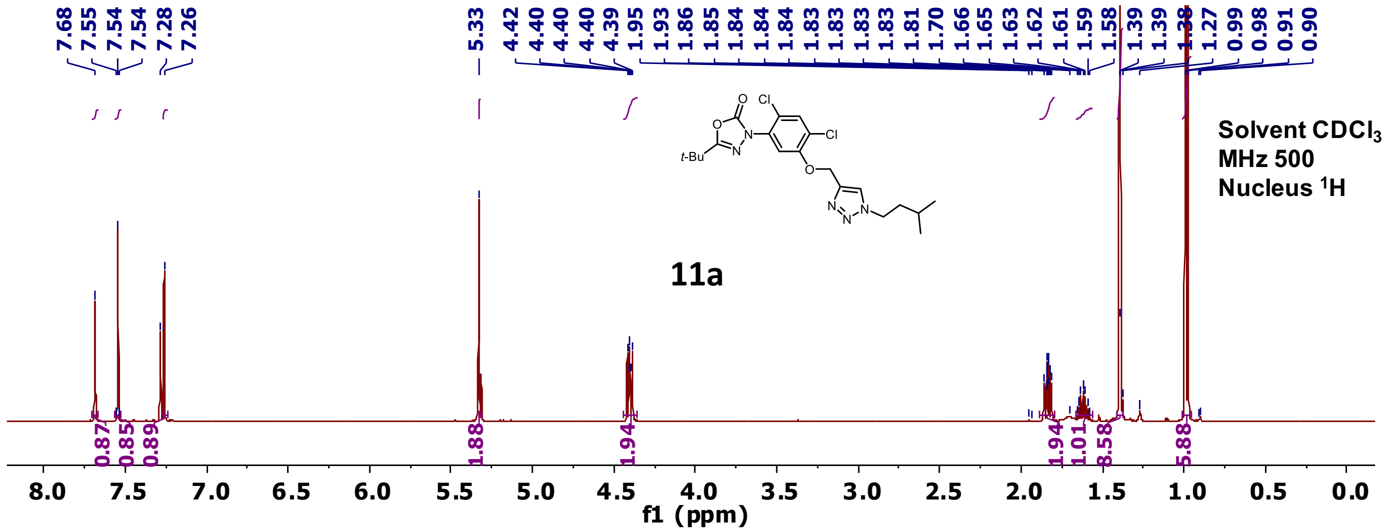
7.68
7.55
7.54
7.54
7.28
7.26
5.33
4.42
4.40
4.40
4.40
4.39
1.95
1.93
1.86
1.85
1.84
1.84
1.84
1.83
1.83
1.83
1.83
1.81
1.70
1.66
1.65
1.63
1.62
1.61
1.59
1.58
1.39
1.39
1.38
1.27
0.99
0.98
0.91
0.90
11a
Solvent CDCl3
MHz 500
Nucleus 1H
0.87
0.85
0.89
1.88
1.94
1.94
1.01
8.58
5.88
8.0 7.5 7.0 6.5 6.0 5.5 5.0 4.5 4.0 3.5 3.0 2.5 2.0 1.5 1.0 0.5 0.0
f1 (ppm)

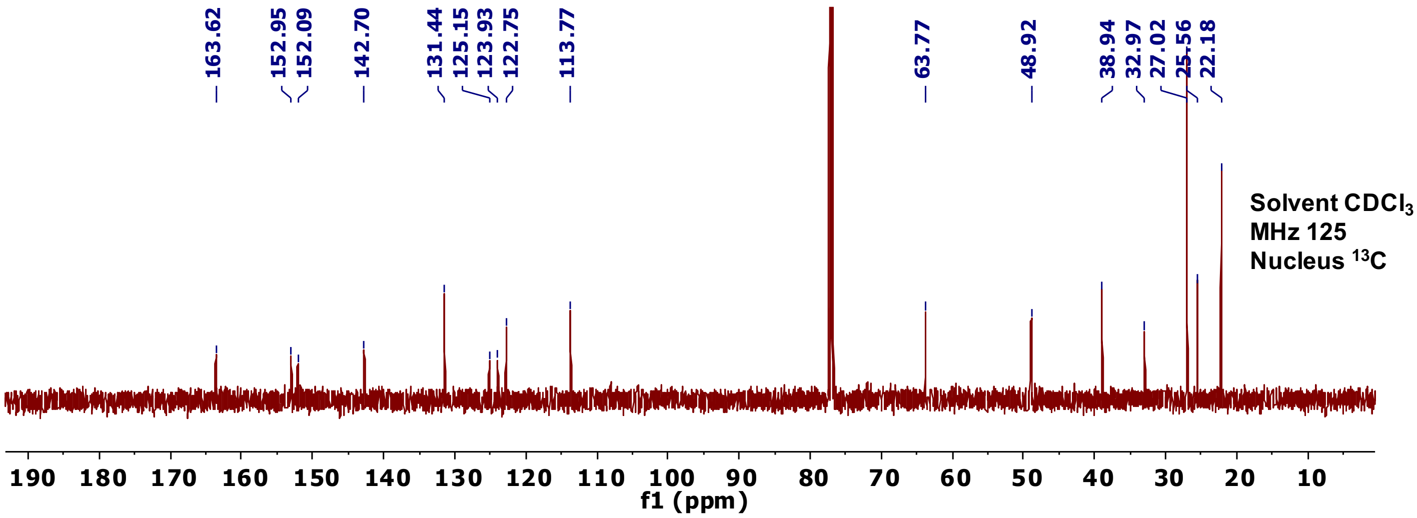
163.62
152.95
152.09
142.70
131.44
125.15
123.93
122.75
113.77
63.77
48.92
38.94
32.97
27.02
25.56
22.18
Solvent CDCl3
MHz 125
Nucleus 13C
190 180 170 160 150 140 130 120 110 100 90 80 70 60 50 40 30 20 10
f1 (ppm)

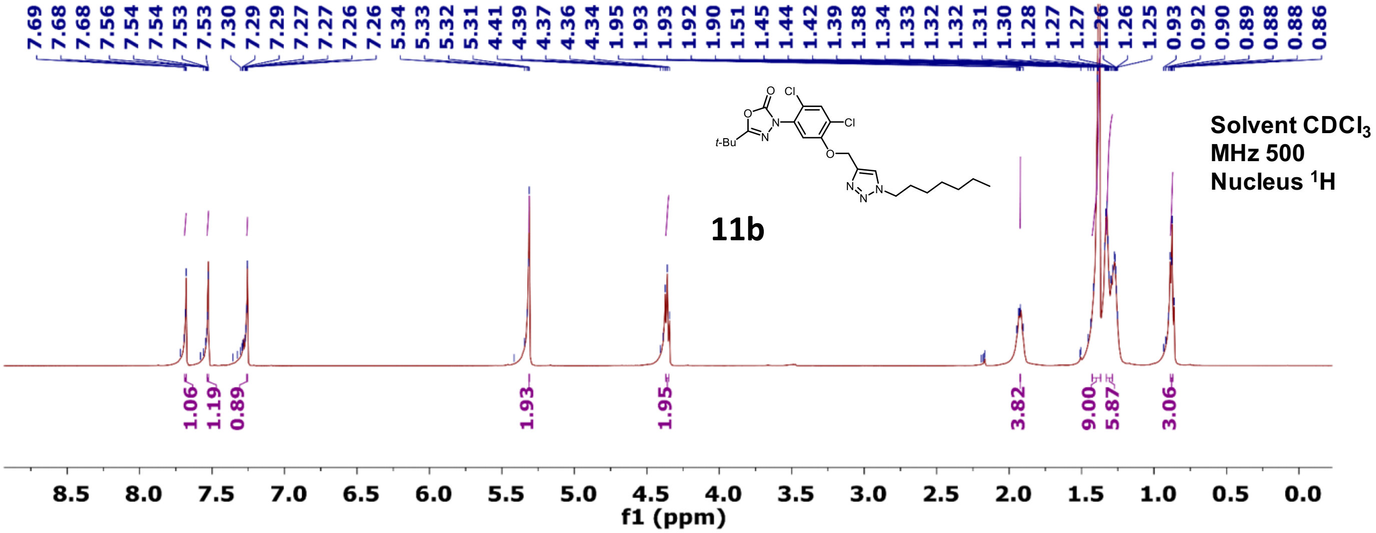

7.69 7.68 7.68 7.56 7.54 7.54 7.53 7.53 7.30 7.29 7.29 7.27 7.27 7.26 7.26 5.34 5.33 5.32 5.31 4.41 4.39 4.37 4.36 4.34 1.95 1.93 1.93 1.92 1.90 1.51 1.45 1.44 1.42 1.39 1.38 1.34 1.33 1.32 1.32 1.31 1.30 1.28 1.27 1.27 1.26 1.26 1.25 0.93 0.92 0.90 0.89 0.88 0.88 0.86
11b
Solvent CDCl3
MHz 500
Nucleus 1H
1.06 1.19 0.89 1.93 1.95 3.82 9.00 5.87 3.06
8.5 8.0 7.5 7.0 6.5 6.0 5.5 5.0 4.5 4.0 3.5 3.0 2.5 2.0 1.5 1.0 0.5 0.0
f1 (ppm)

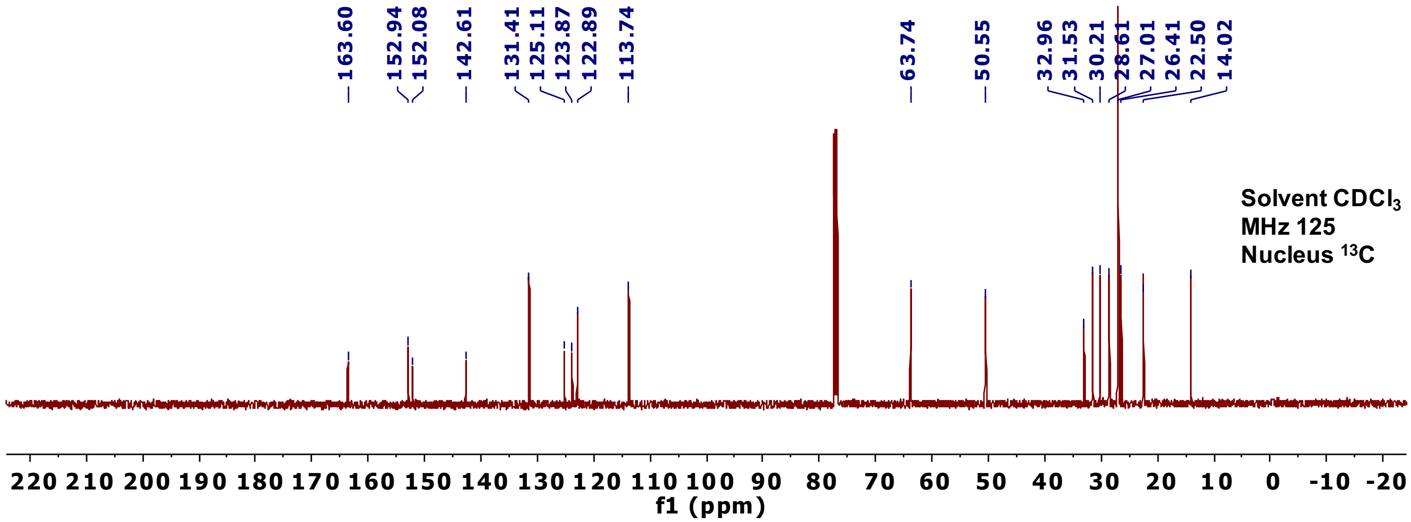

163.60 152.94 152.08 142.61 131.41 125.11 123.87 122.89 113.74 63.74 50.55 32.96 31.53 30.21 28.61 27.01 26.41 22.50 14.02
Solvent CDCl3
MHz 125
Nucleus 13C
220 210 200 190 180 170 160 150 140 130 120 110 100 90 80 70 60 50 40 30 20 10 0 -10 -20
f1 (ppm)

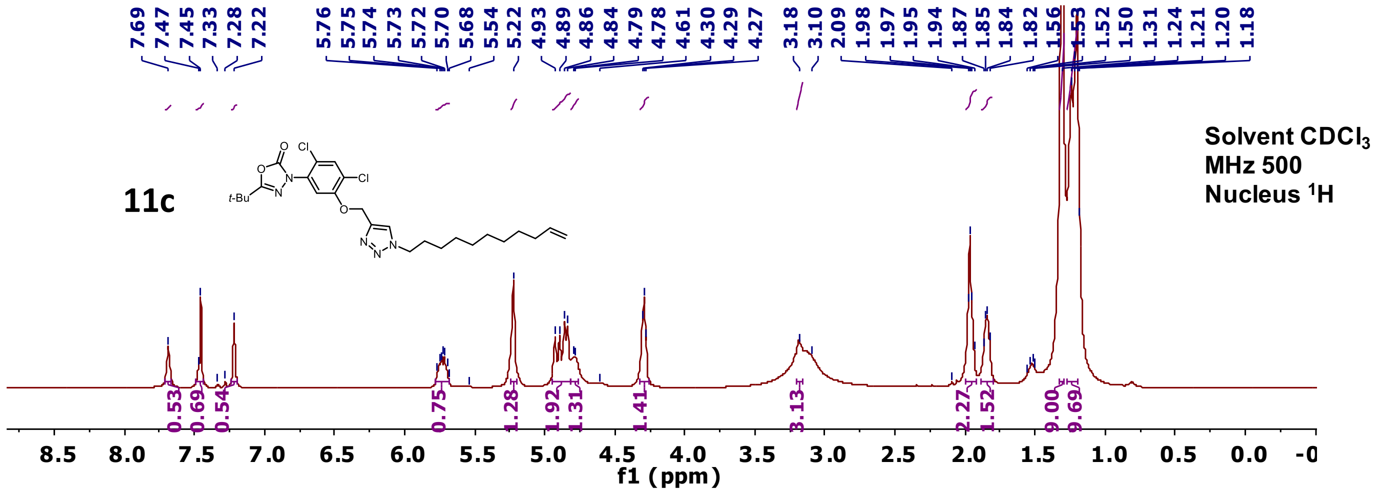

7.69 7.47 7.45 7.33 7.28 7.22 5.76 5.75 5.74 5.73 5.72 5.70 5.68 5.54 5.22 4.93 4.89 4.86 4.84 4.79 4.78 4.61 4.30 4.29 4.27 3.18 3.10 2.09 1.98 1.97 1.95 1.94 1.87 1.85 1.84 1.82 1.56 1.53 1.52 1.50 1.31 1.24 1.21 1.20 1.18
11c
t-Bu
Solvent CDCl3
MHz 500
Nucleus 1H
0.53 0.69 0.54 0.75 1.28 1.92 1.31 1.41 3.13 2.27 1.52 9.00 9.69
f1 (ppm)

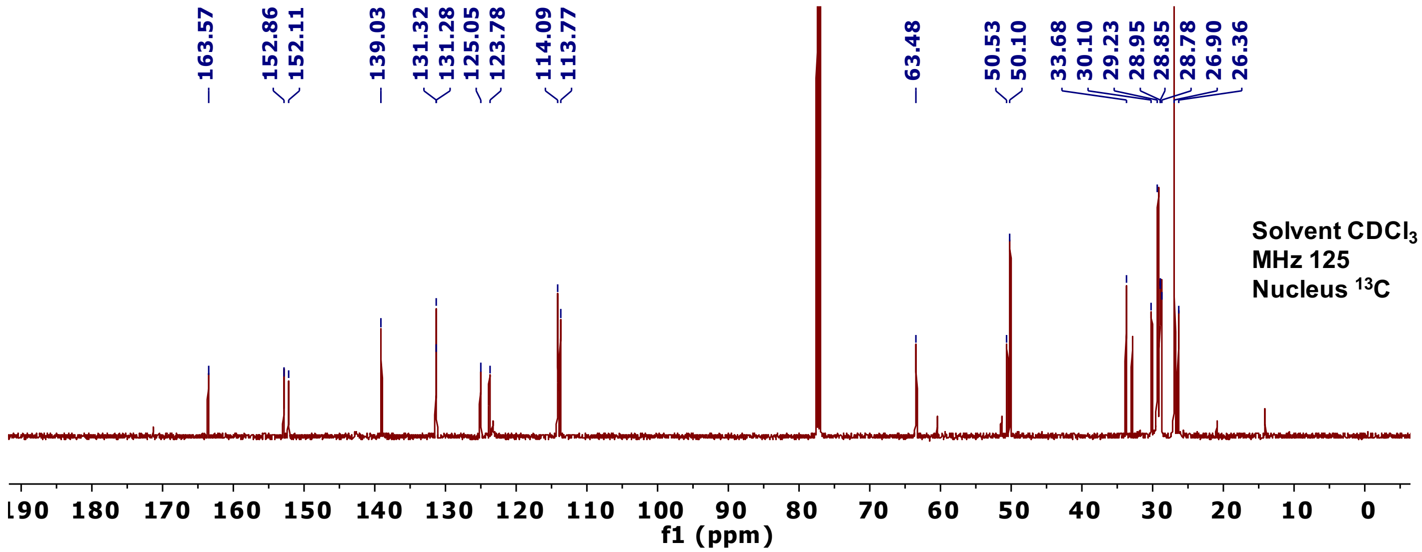

163.57 152.86 152.11 139.03 131.32 131.28 125.05 123.78 114.09 113.77 63.48 50.53 50.10 33.68 30.10 29.23 28.95 28.85 28.78 26.90 26.36
Solvent CDCl3
MHz 125
Nucleus 13C
f1 (ppm)

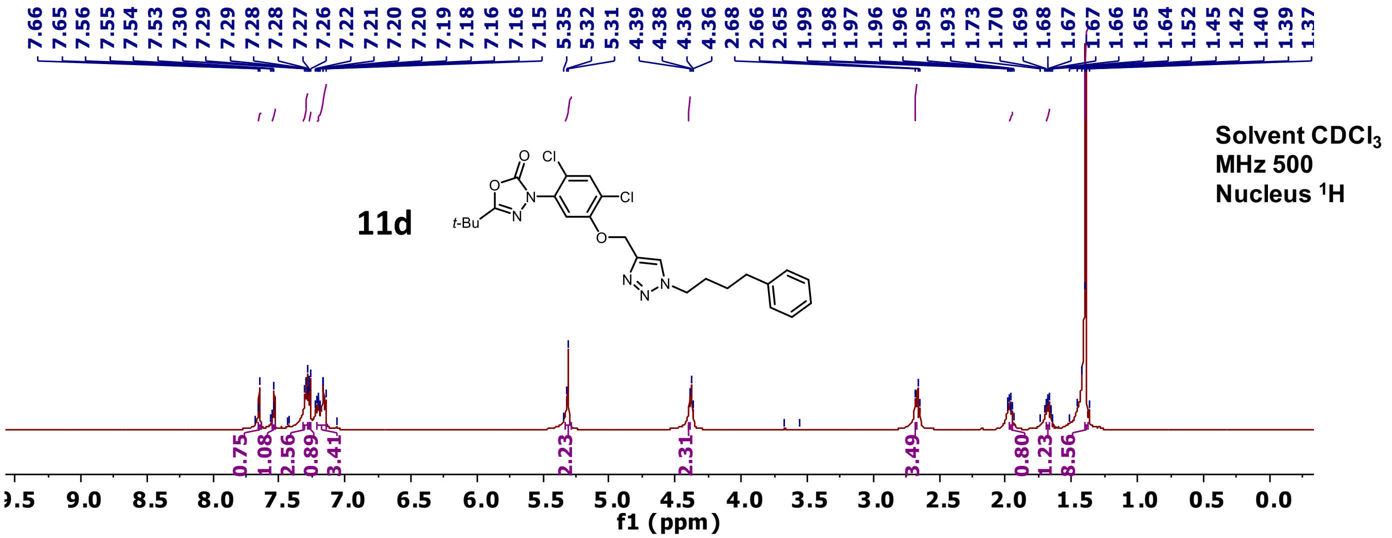
7.66 7.65 7.56 7.55 7.54 7.53 7.30 7.29 7.29 7.28 7.28 7.27 7.26 7.22 7.21 7.20 7.20 7.19 7.18 7.16 7.16 7.15 5.35 5.32 5.31 4.39 4.38 4.36 4.36 2.68 2.66 2.65 1.99 1.98 1.97 1.96 1.96 1.95 1.93 1.73 1.70 1.69 1.68 1.67 1.67 1.66 1.65 1.64 1.52 1.45 1.42 1.40 1.39 1.37
11d
t-Bu
Cl
Cl
Solvent $CDCl_3$
MHz 500
Nucleus ^1H
0.75 1.08 2.56 0.89 3.41 2.23 2.31 3.49 0.80 1.23 8.56
f1 (ppm)

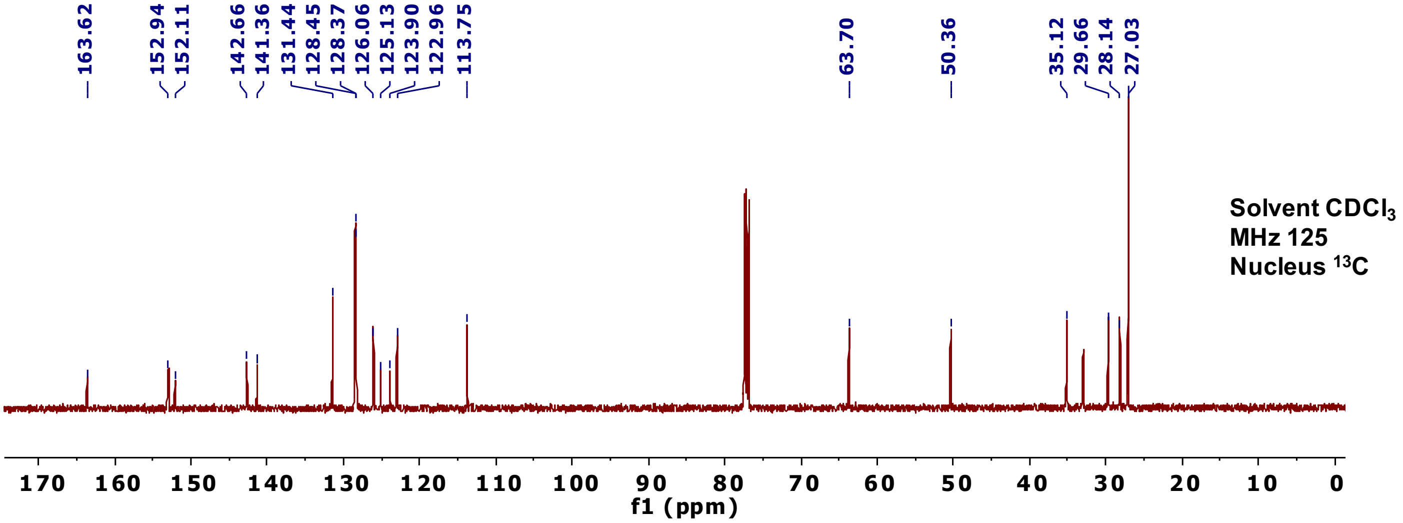
163.62 152.94 152.11 142.66 141.36 131.44 128.45 128.37 126.06 125.13 123.90 122.96 113.75 63.70 50.36 35.12 29.66 28.14 27.03
Solvent $CDCl_3$
MHz 125
Nucleus ^{13}C
f1 (ppm)

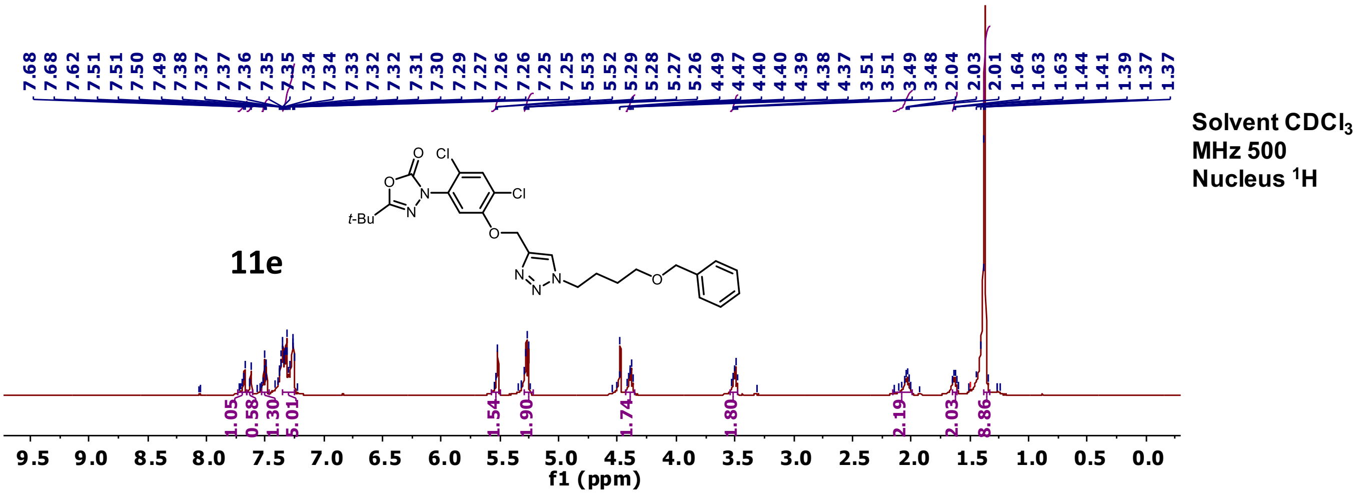
7.68 7.68 7.62 7.51 7.51 7.50 7.49 7.38 7.37 7.37 7.36 7.35 7.35 7.34 7.34 7.33 7.32 7.32 7.31 7.30 7.29 7.27 7.26 7.26 7.25 7.25 5.53 5.52 5.29 5.28 5.27 5.26 4.49 4.47 4.40 4.40 4.39 4.38 4.37 3.51 3.51 3.49 3.48 2.04 2.03 2.01 1.64 1.63 1.63 1.44 1.41 1.39 1.37 1.37
Solvent CDCl3
MHz 500
Nucleus 1H
O
O
N
N
Cl
Cl
t-Bu
N
O
N
N
N
O
11e
1.05
0.58
1.30
5.01
1.54
1.90
1.74
1.80
2.19
2.03
8.86
9.5 9.0 8.5 8.0 7.5 7.0 6.5 6.0 5.5 5.0 4.5 4.0 3.5 3.0 2.5 2.0 1.5 1.0 0.5 0.0
f1 (ppm)

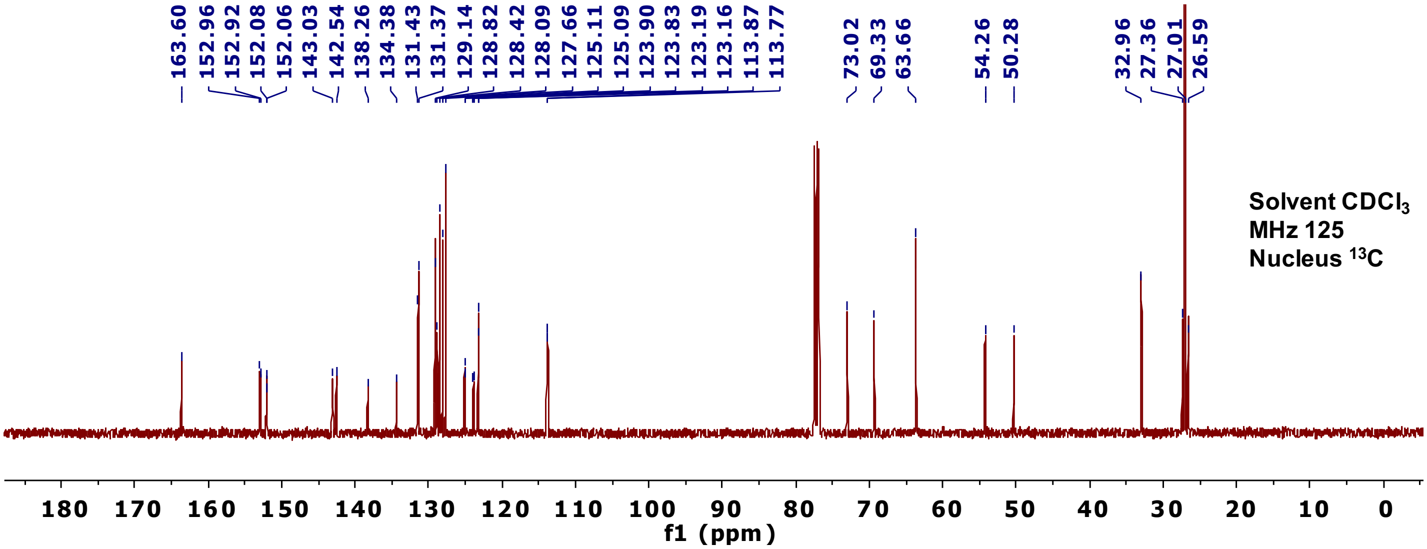
163.60 152.96 152.92 152.08 152.06 143.03 142.54 138.26 134.38 131.43 131.37 129.14 128.82 128.42 128.09 127.66 125.11 125.09 123.90 123.83 123.19 123.16 113.87 113.77 73.02 69.33 63.66 54.26 50.28 32.96 27.36 27.01 26.59
Solvent CDCl3
MHz 125
Nucleus 13C
180 170 160 150 140 130 120 110 100 90 80 70 60 50 40 30 20 10 0
f1 (ppm)

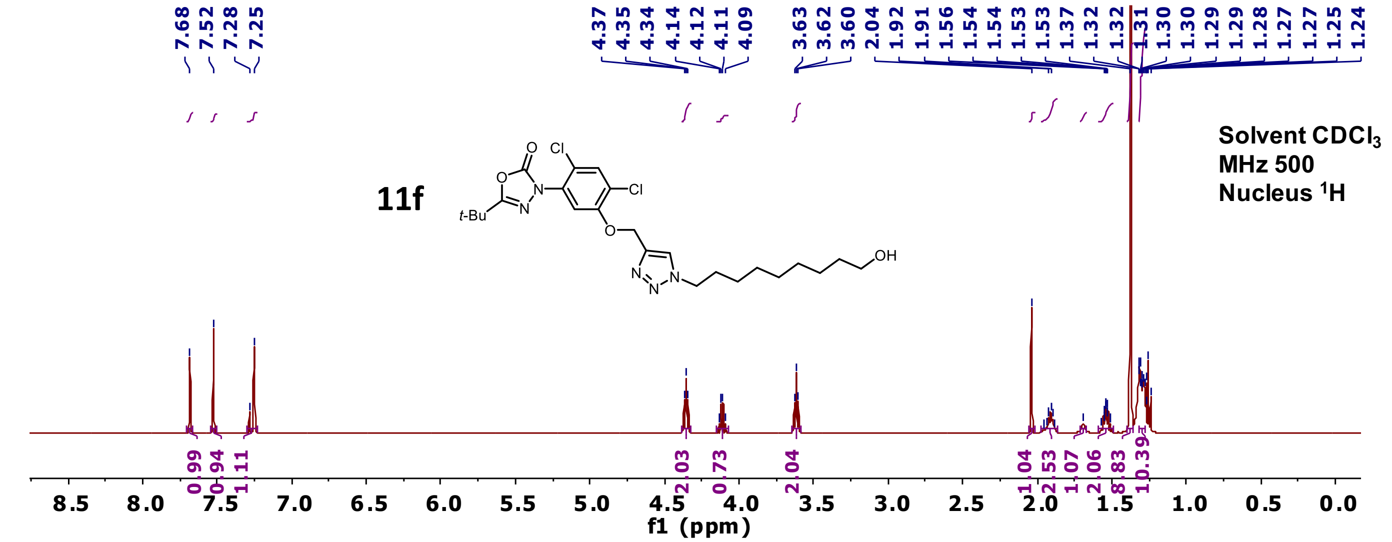
7.68 7.52 7.28 7.25
4.37 4.35 4.34 4.14 4.12 4.11 4.09
3.63 3.62 3.60 2.04 1.92 1.91 1.56 1.54 1.54 1.53 1.53 1.37 1.32 1.32 1.31 1.30 1.29 1.29 1.28 1.27 1.27 1.25 1.24
11f
t-Bu
OH
Solvent CDCl3
MHz 500
Nucleus 1H
0.99 0.94 1.11 2.03 0.73 2.04 1.04 2.53 1.07 2.06 8.83 10.39
8.5 8.0 7.5 7.0 6.5 6.0 5.5 5.0 4.5 4.0 3.5 3.0 2.5 2.0 1.5 1.0 0.5 0.0
f1 (ppm)

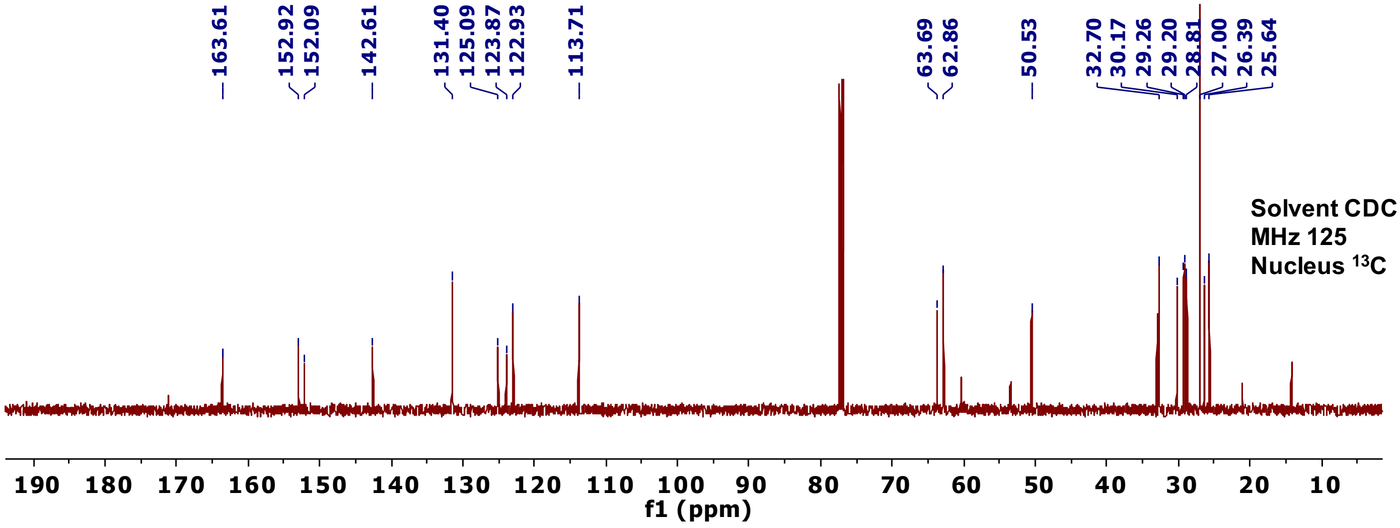
163.61
152.92 152.09
142.61
131.40 125.09 123.87 122.93
113.71
63.69 62.86
50.53
32.70 30.17 29.26 29.20 28.81 27.00 26.39 25.64
Solvent CDCl3
MHz 125
Nucleus 13C
190 180 170 160 150 140 130 120 110 100 90 80 70 60 50 40 30 20 10
f1 (ppm)

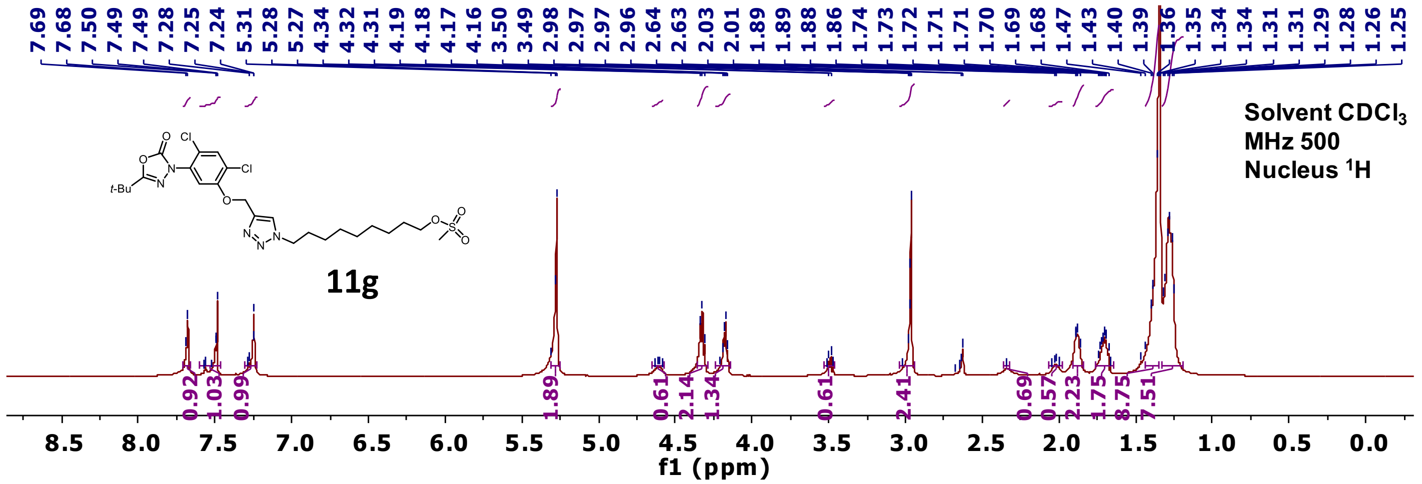
7.69 7.68 7.50 7.49 7.49 7.28 7.25 7.24 5.31 5.28 5.27 4.34 4.32 4.31 4.19 4.18 4.17 4.16 3.50 3.49 2.98 2.97 2.97 2.96 2.64 2.63 2.03 2.01 1.89 1.89 1.88 1.86 1.74 1.73 1.72 1.71 1.71 1.70 1.69 1.68 1.47 1.43 1.40 1.39 1.36 1.35 1.34 1.34 1.31 1.31 1.29 1.28 1.26 1.25
Solvent $CDCl_3$
MHz 500
Nucleus ^1H
11g
t-Bu
0.92 1.03 0.99 1.89 0.61 2.14 1.34 0.61 2.41 0.69 0.57 2.23 1.75 8.75 7.51
f1 (ppm)

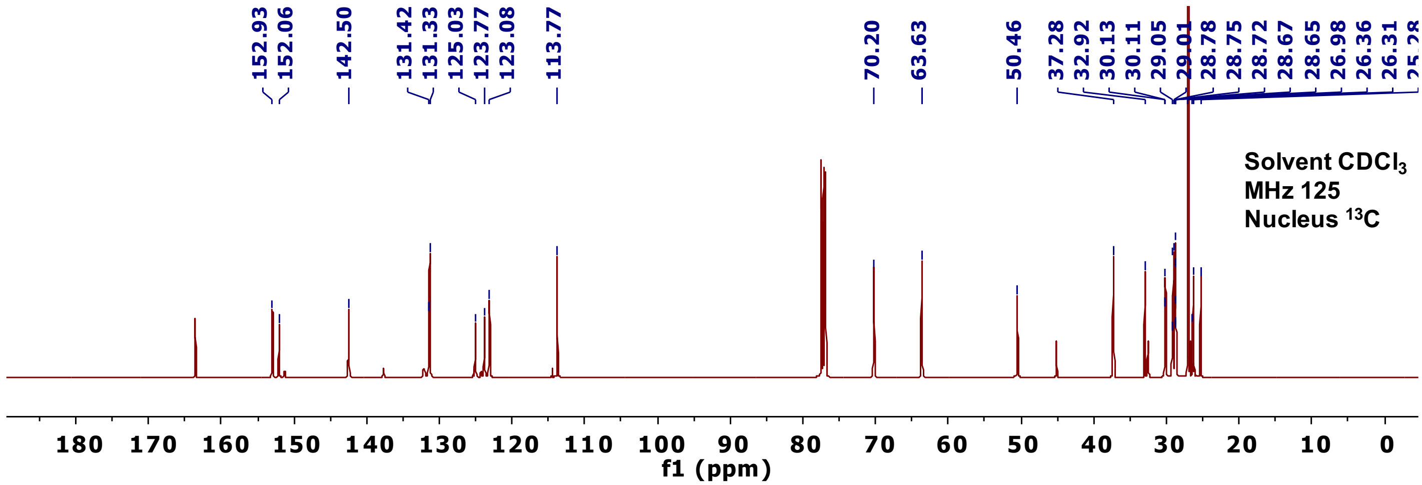
152.93 152.06 142.50 131.42 131.33 125.03 123.77 123.08 113.77 70.20 63.63 50.46 37.28 32.92 30.13 30.11 29.05 29.01 28.78 28.75 28.72 28.67 28.65 26.98 26.36 26.31
Solvent $CDCl_3$
MHz 125
Nucleus ^{13}C
f1 (ppm)

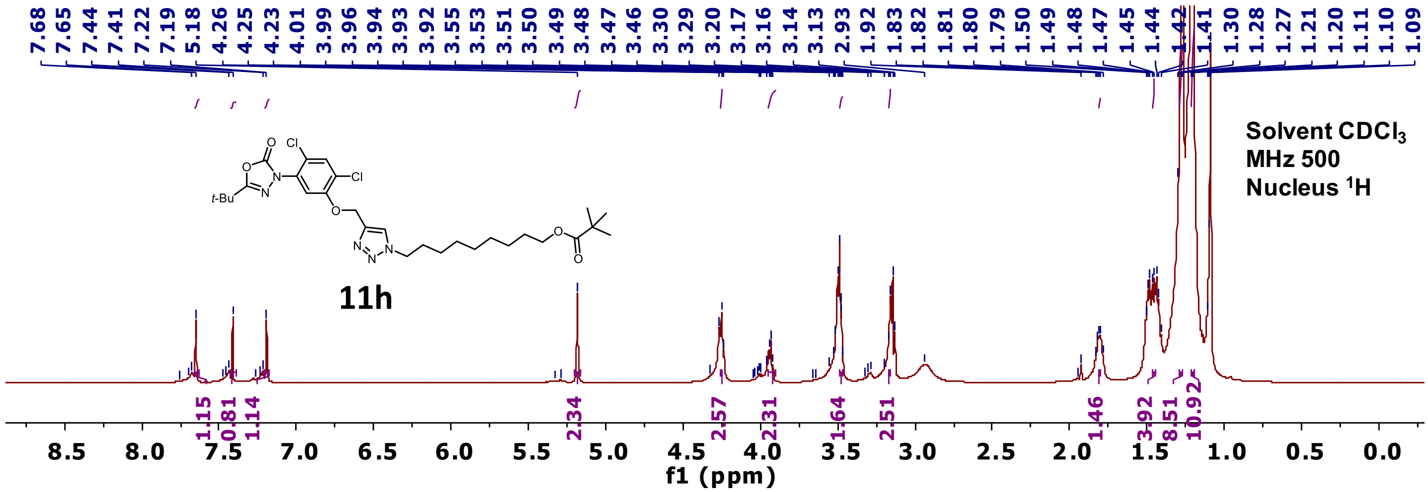
7.68 7.65 7.44 7.41 7.22 7.19 5.18 4.26 4.25 4.23 4.01 3.99 3.96 3.94 3.93 3.92 3.55 3.53 3.51 3.50 3.49 3.48 3.47 3.46 3.30 3.29 3.20 3.17 3.16 3.14 3.13 2.93 1.92 1.83 1.82 1.81 1.80 1.79 1.50 1.49 1.48 1.47 1.45 1.44 1.42 1.41 1.30 1.28 1.27 1.21 1.20 1.11 1.10 1.09
Solvent $CDCl_3$
MHz 500
Nucleus ^1H
t-Bu
11h
1.15 0.81 1.14 2.34 2.57 2.31 1.64 2.51 1.46 3.92 8.51 10.92
8.5 8.0 7.5 7.0 6.5 6.0 5.5 5.0 4.5 4.0 3.5 3.0 2.5 2.0 1.5 1.0 0.5 0.0
f1 (ppm)

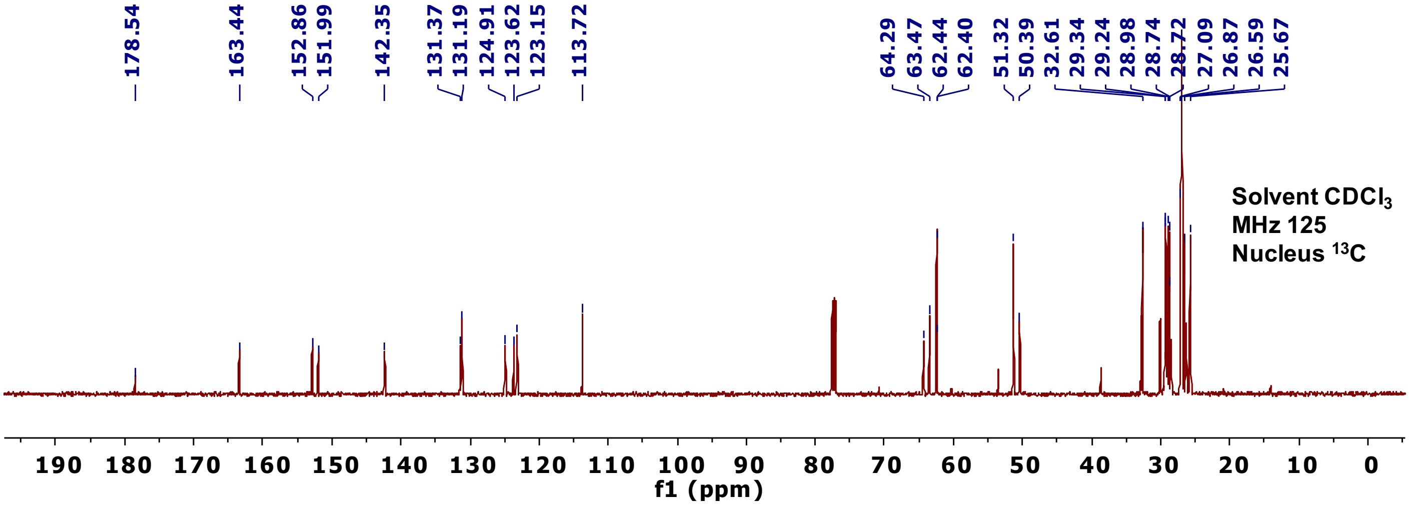
178.54 163.44 152.86 151.99 142.35 131.37 131.19 124.91 123.62 123.15 113.72
64.29 63.47 62.44 62.40 51.32 50.39 32.61 29.34 29.24 28.98 28.74 28.72 27.09 26.87 26.59 25.67
Solvent $CDCl_3$
MHz 125
Nucleus ^{13}C
190 180 170 160 150 140 130 120 110 100 90 80 70 60 50 40 30 20 10 0
f1 (ppm)

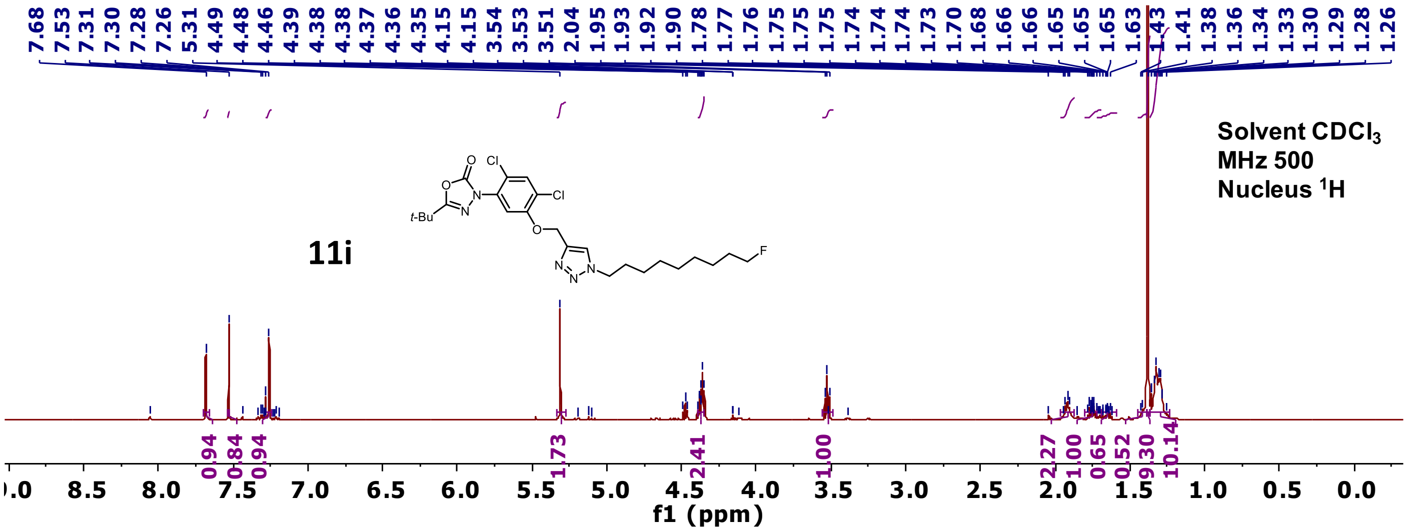

7.68 7.53 7.31 7.30 7.28 7.26 5.31 4.49 4.48 4.46 4.39 4.38 4.38 4.37 4.36 4.35 4.15 4.15 3.54 3.53 3.51 2.04 1.95 1.93 1.92 1.90 1.78 1.77 1.76 1.75 1.75 1.75 1.74 1.74 1.74 1.73 1.70 1.68 1.66 1.66 1.65 1.65 1.65 1.63 1.43 1.41 1.38 1.36 1.34 1.33 1.30 1.29 1.28 1.26
Solvent CDCl3
MHz 500
Nucleus 1H
11i
t-Bu
Cl
Cl
F
0.94 0.84 0.94 1.73 2.41 1.00 2.27 1.00 0.65 0.52 9.30 10.14
f1 (ppm)

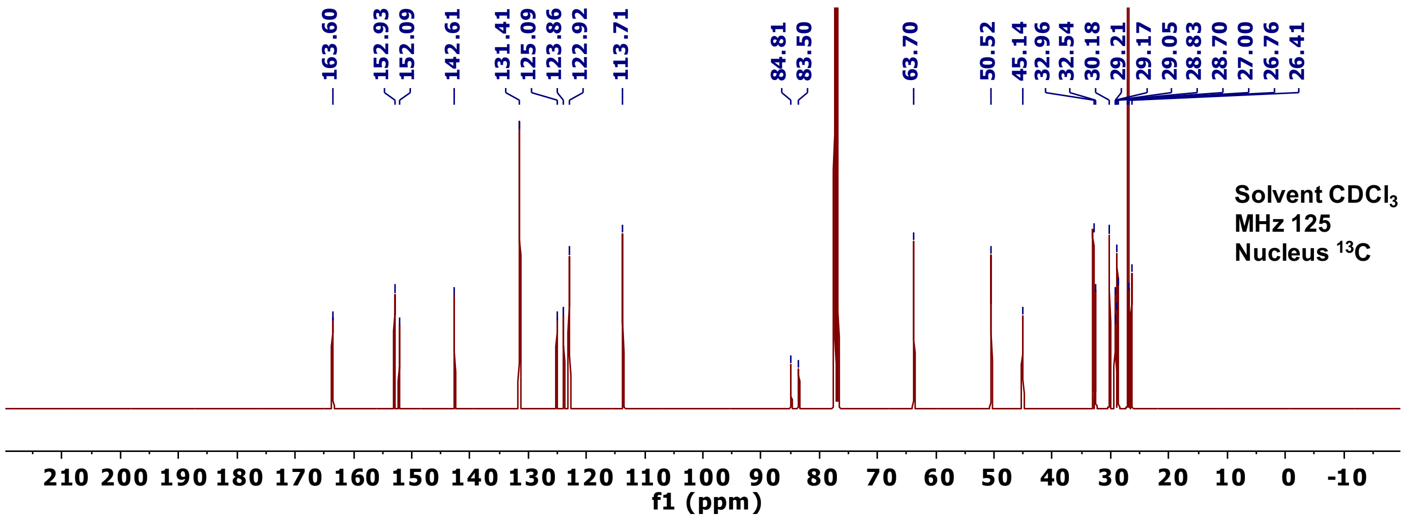

163.60 152.93 152.09 142.61 131.41 125.09 123.86 122.92 113.71 84.81 83.50 63.70 50.52 45.14 32.96 32.54 30.18 29.21 29.17 29.05 28.83 28.70 27.00 26.76 26.41
Solvent CDCl3
MHz 125
Nucleus 13C
f1 (ppm)

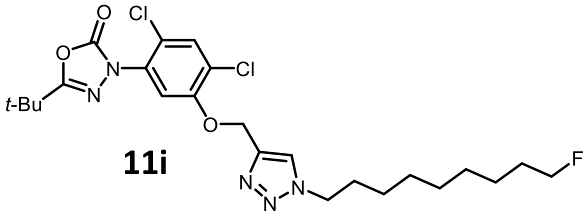

11i

Solvent $CDCl_3$
MHz 470
Nucleus ^{19}F

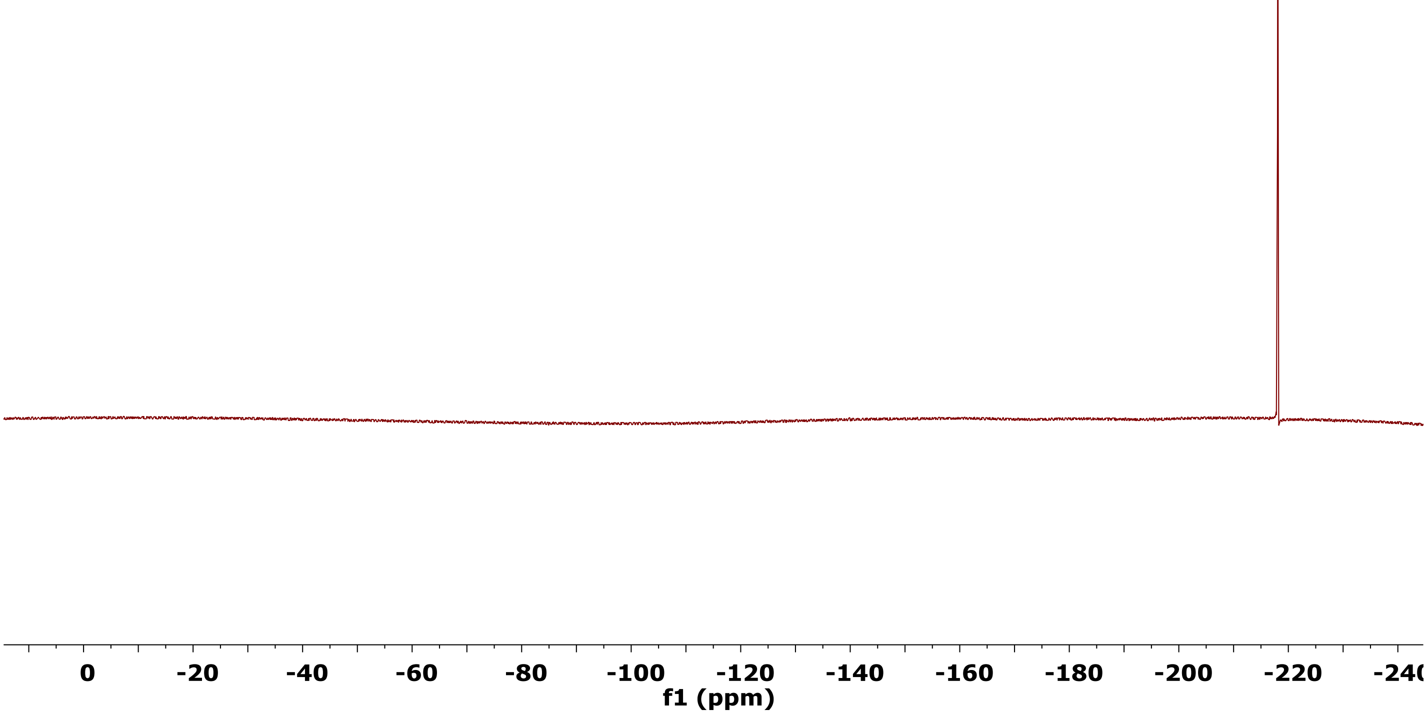

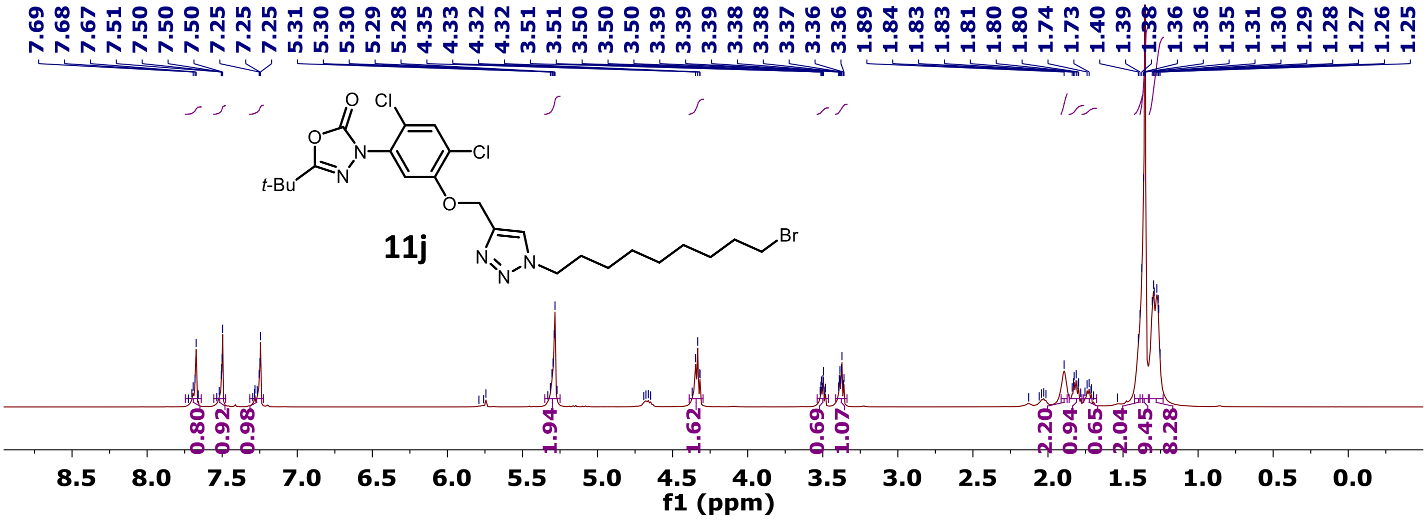
7.69
7.68
7.67
7.51
7.50
7.50
7.50
7.25
7.25
7.25
5.31
5.30
5.29
5.28
4.35
4.33
4.32
4.32
3.51
3.51
3.50
3.50
3.50
3.39
3.39
3.39
3.38
3.38
3.37
3.36
3.36
1.89
1.84
1.83
1.83
1.81
1.80
1.80
1.74
1.73
1.40
1.39
1.38
1.36
1.36
1.35
1.31
1.30
1.29
1.28
1.27
1.26
1.25
11j
t-Bu
Cl
Cl
Br
0.80
0.92
0.98
1.94
1.62
0.69
1.07
2.20
0.94
0.65
2.04
9.45
8.28
8.5
8.0
7.5
7.0
6.5
6.0
5.5
5.0
4.5
4.0
3.5
3.0
2.5
2.0
1.5
1.0
0.5
0.0
f1 (ppm)

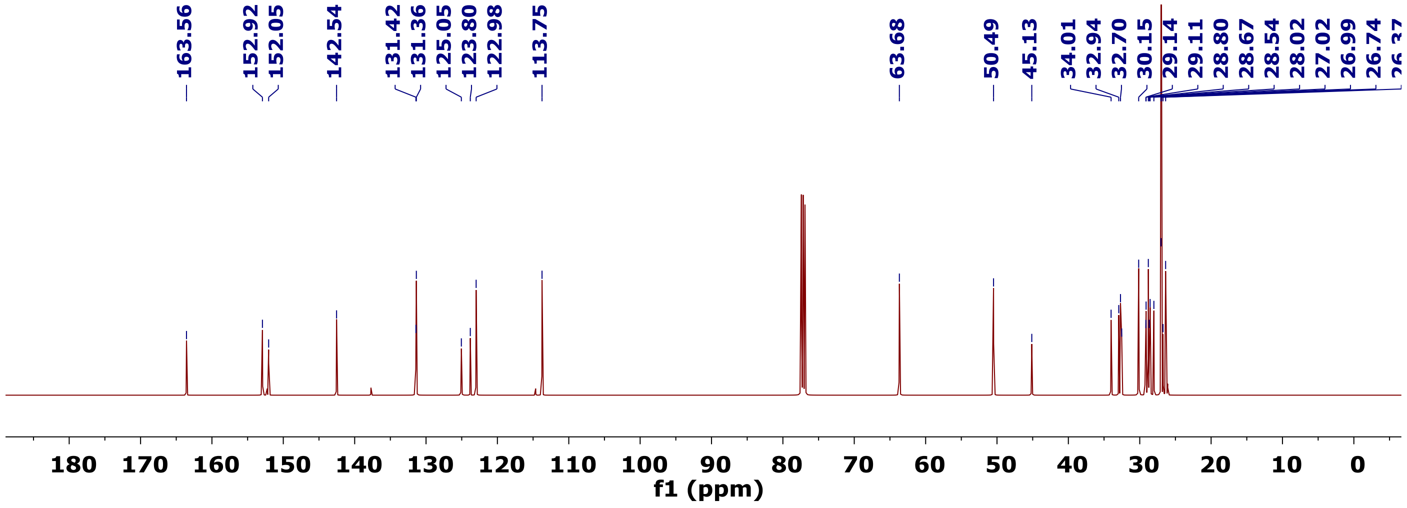
163.56
152.92
152.05
142.54
131.42
131.36
125.05
123.80
122.98
113.75
63.68
50.49
45.13
34.01
32.94
32.70
30.15
29.14
29.11
28.80
28.67
28.54
28.02
27.02
26.99
26.74
180
170
160
150
140
130
120
110
100
90
80
70
60
50
40
30
20
10
0
f1 (ppm)

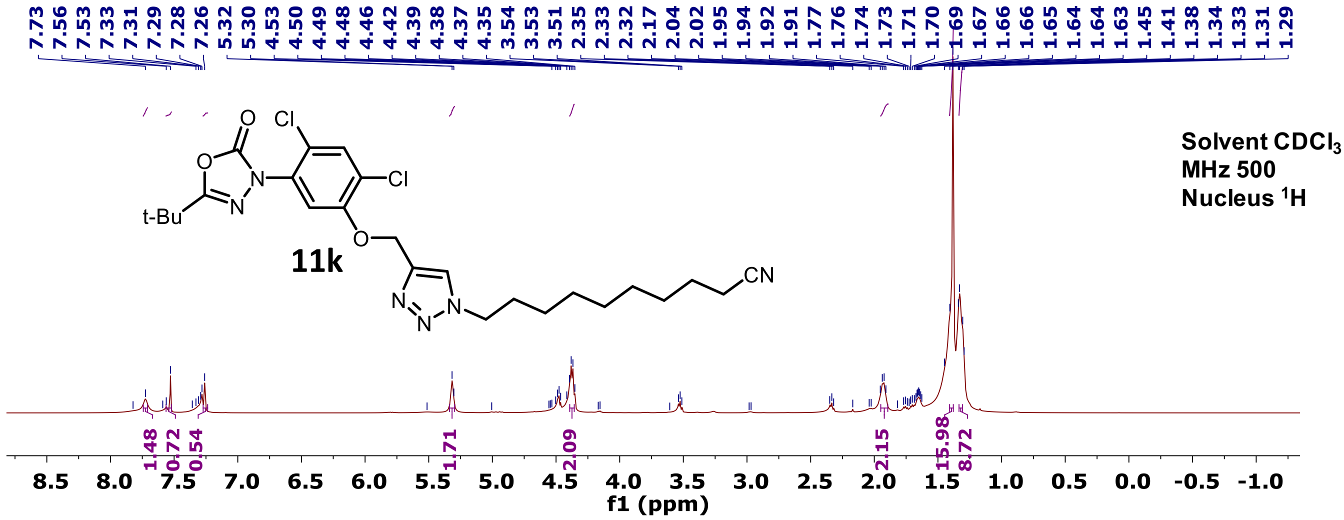

11k
Solvent CDCl3
MHz 500
Nucleus 1H
f1 (ppm)

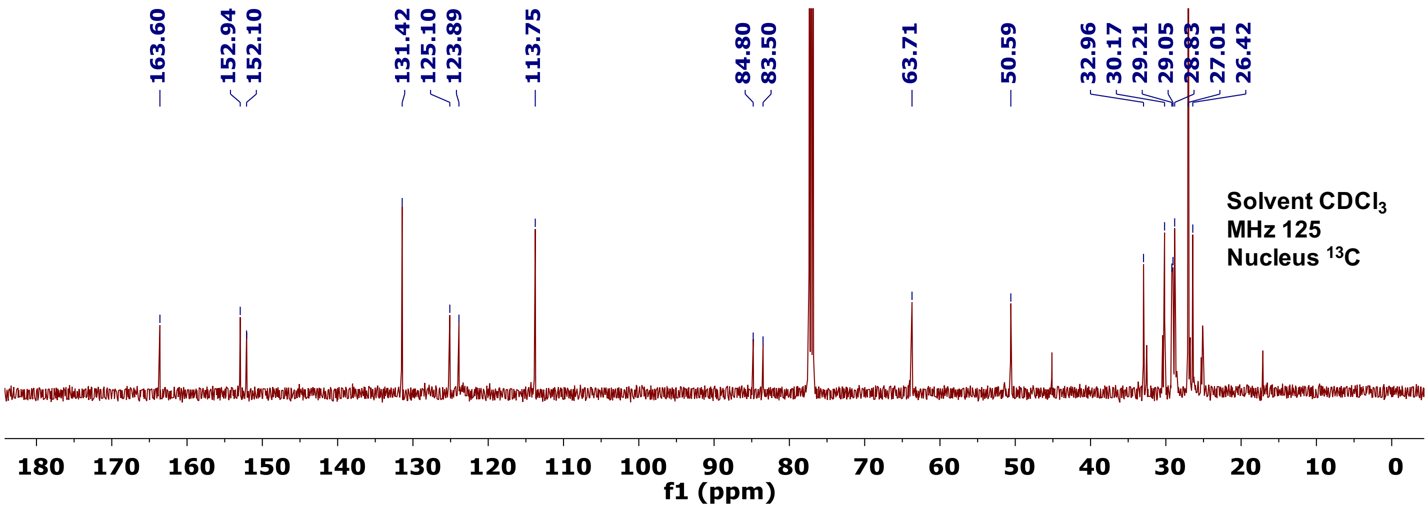

Solvent CDCl3
MHz 125
Nucleus 13C
f1 (ppm)

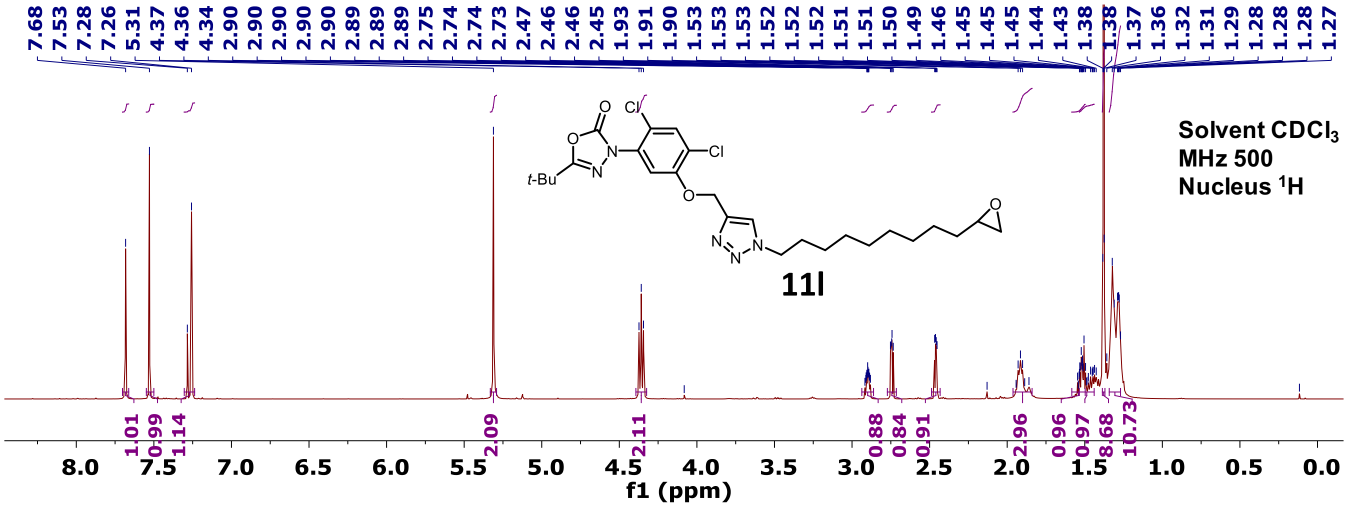
7.68
7.53
7.28
7.26
5.31
4.37
4.36
4.34
2.90
2.90
2.90
2.90
2.90
2.89
2.89
2.89
2.75
2.74
2.74
2.73
2.47
2.46
2.46
2.45
1.93
1.91
1.90
1.53
1.53
1.53
1.52
1.52
1.52
1.51
1.51
1.50
1.49
1.46
1.45
1.45
1.45
1.44
1.43
1.38
1.38
1.37
1.36
1.32
1.31
1.29
1.28
1.28
1.28
1.27
t-Bu
Cl
Cl
11l
Solvent $CDCl_3$
MHz 500
Nucleus ^1H
1.01
0.99
1.14
2.09
2.11
0.88
0.84
0.91
2.96
0.96
0.97
8.68
10.73
8.0
7.5
7.0
6.5
6.0
5.5
5.0
4.5
4.0
3.5
3.0
2.5
2.0
1.5
1.0
0.5
0.0
f1 (ppm)

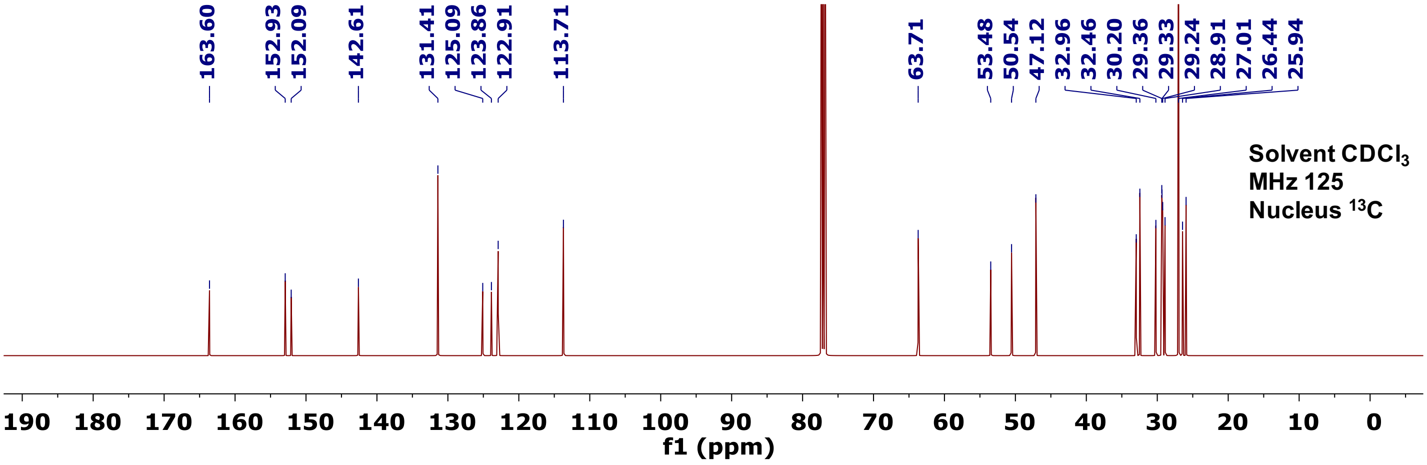
163.60
152.93
152.09
142.61
131.41
125.09
123.86
122.91
113.71
63.71
53.48
50.54
47.12
32.96
32.46
30.20
29.36
29.33
29.24
28.91
27.01
26.44
25.94
Solvent $CDCl_3$
MHz 125
Nucleus ^{13}C
190
180
170
160
150
140
130
120
110
100
90
80
70
60
50
40
30
20
10
0
f1 (ppm)

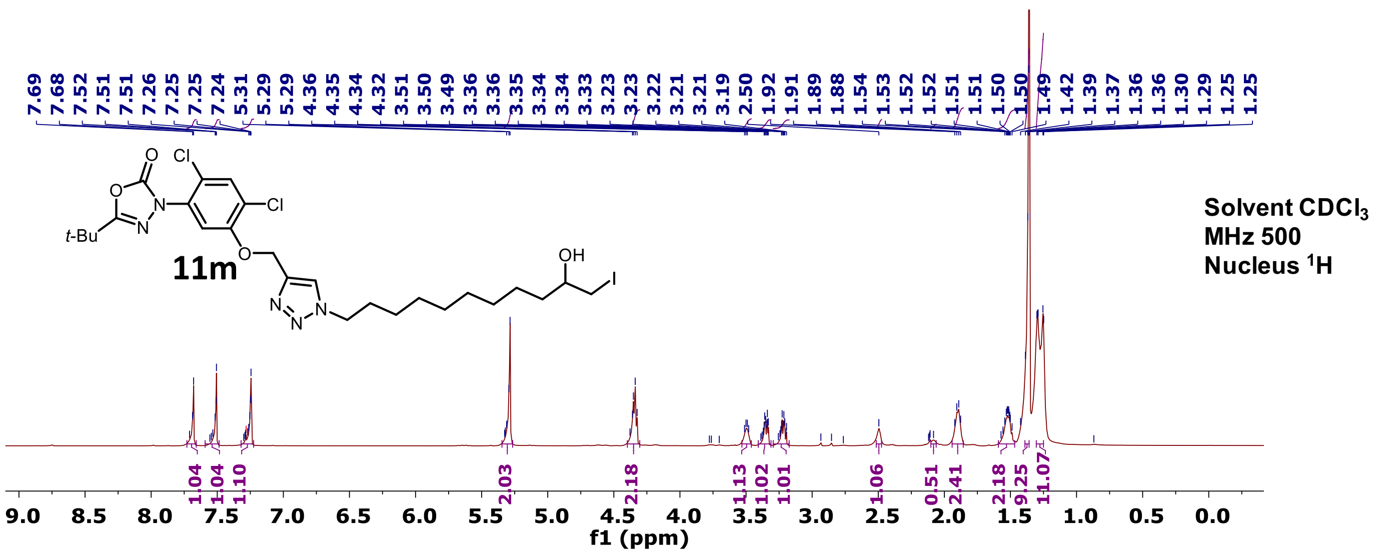

7.69 7.68 7.52 7.51 7.51 7.26 7.25 7.25 7.24 5.31 5.29 4.36 4.35 4.34 4.32 3.51 3.50 3.49 3.36 3.36 3.35 3.34 3.34 3.33 3.23 3.23 3.22 3.21 3.21 3.19 2.50 1.92 1.91 1.89 1.88 1.54 1.53 1.52 1.52 1.51 1.51 1.50 1.50 1.49 1.42 1.39 1.37 1.36 1.36 1.30 1.29 1.25 1.25
O
Cl
Cl
N
O
N
t-Bu
O
11m
OH
I
N
N
N
Solvent CDCl3
MHz 500
Nucleus 1H
1.04 1.04 1.10 2.03 2.18 1.13 1.02 1.01 1.06 0.51 2.41 2.18 9.25 11.07
9.0 8.5 8.0 7.5 7.0 6.5 6.0 5.5 5.0 4.5 4.0 3.5 3.0 2.5 2.0 1.5 1.0 0.5 0.0
f1 (ppm)

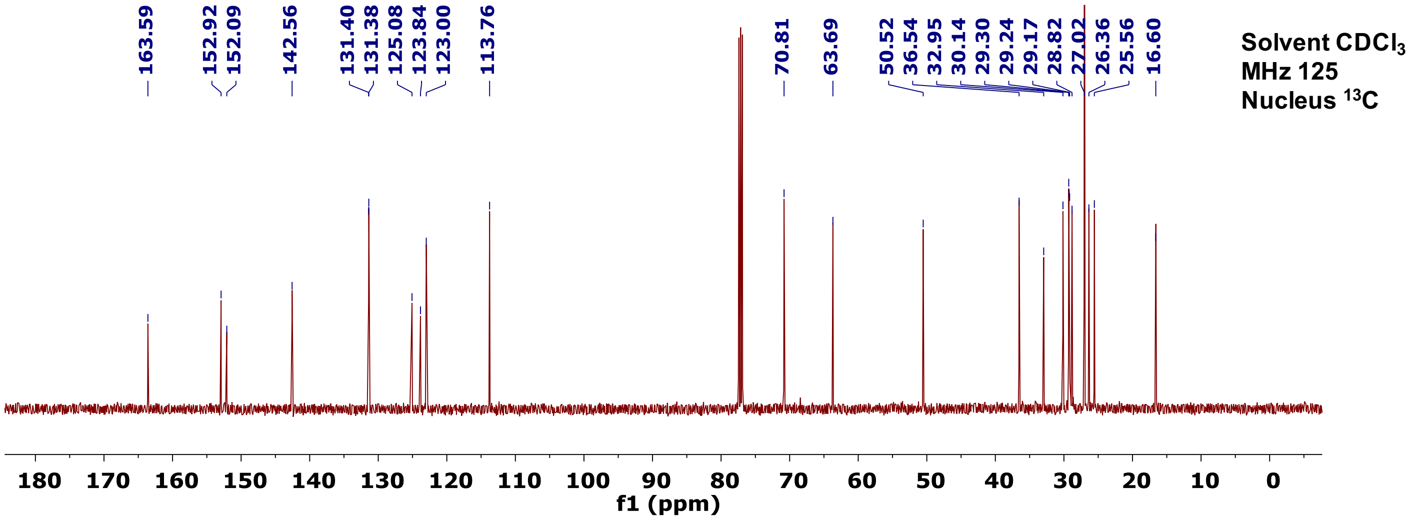

163.59 152.92 152.09 142.56 131.40 131.38 125.08 123.84 123.00 113.76 70.81 63.69 50.52 36.54 32.95 30.14 29.30 29.24 29.17 28.82 27.02 26.36 25.56 16.60
Solvent CDCl3
MHz 125
Nucleus 13C
180 170 160 150 140 130 120 110 100 90 80 70 60 50 40 30 20 10 0
f1 (ppm)

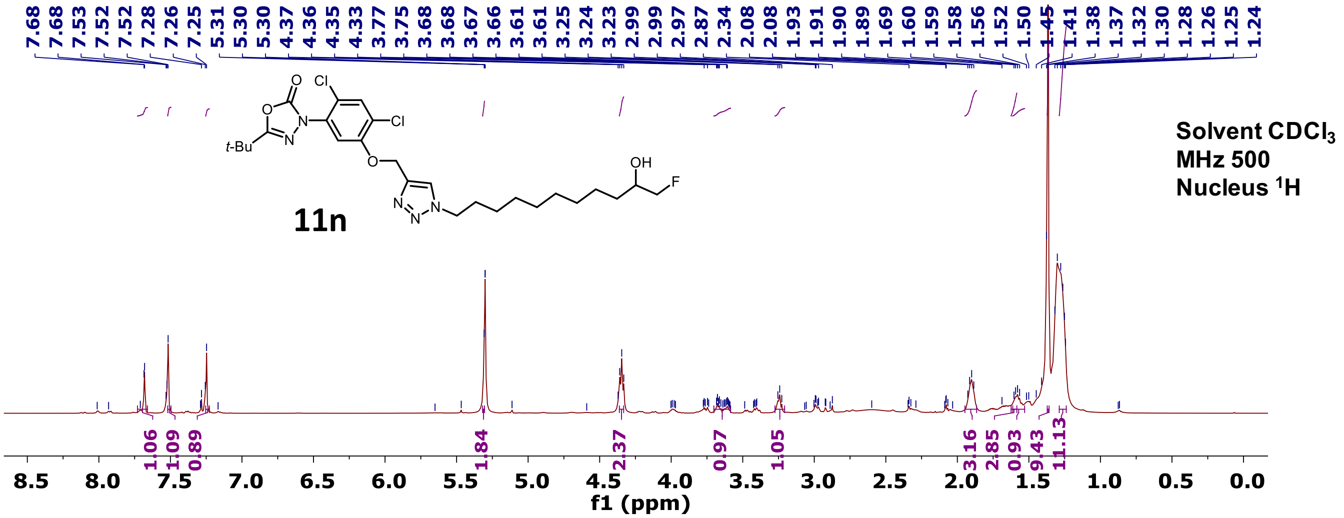
7.68 7.68 7.53 7.52 7.52 7.28 7.26 7.25 5.31 5.30 4.37 4.36 4.35 4.33 3.77 3.75 3.68 3.68 3.67 3.66 3.61 3.61 3.25 3.24 3.23 2.99 2.99 2.97 2.87 2.34 2.08 2.08 1.93 1.91 1.90 1.89 1.69 1.60 1.59 1.58 1.56 1.52 1.50 1.45 1.41 1.38 1.37 1.32 1.30 1.28 1.26 1.25 1.24
11n
Solvent $CDCl_3$
MHz 500
Nucleus ^1H
1.06 1.09 0.89 1.84 2.37 0.97 1.05 3.16 2.85 0.93 9.43 11.13
f1 (ppm)

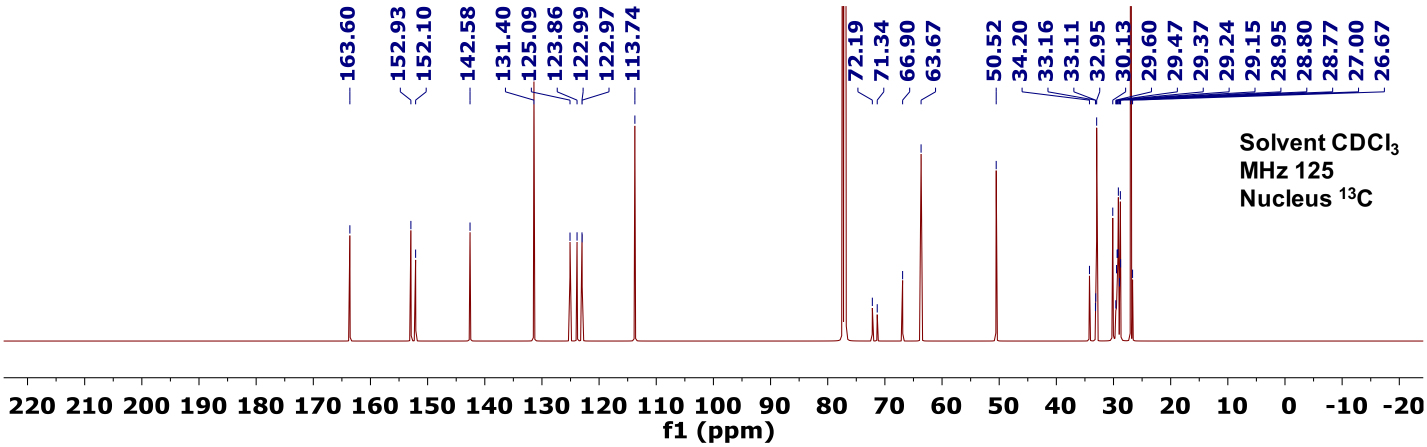
163.60 152.93 152.10 142.58 131.40 125.09 123.86 122.99 122.97 113.74 72.19 71.34 66.90 63.67 50.52 34.20 33.16 33.11 32.95 30.13 29.60 29.47 29.37 29.24 29.15 28.95 28.80 28.77 27.00 26.67
Solvent $CDCl_3$
MHz 125
Nucleus ^{13}C
f1 (ppm)

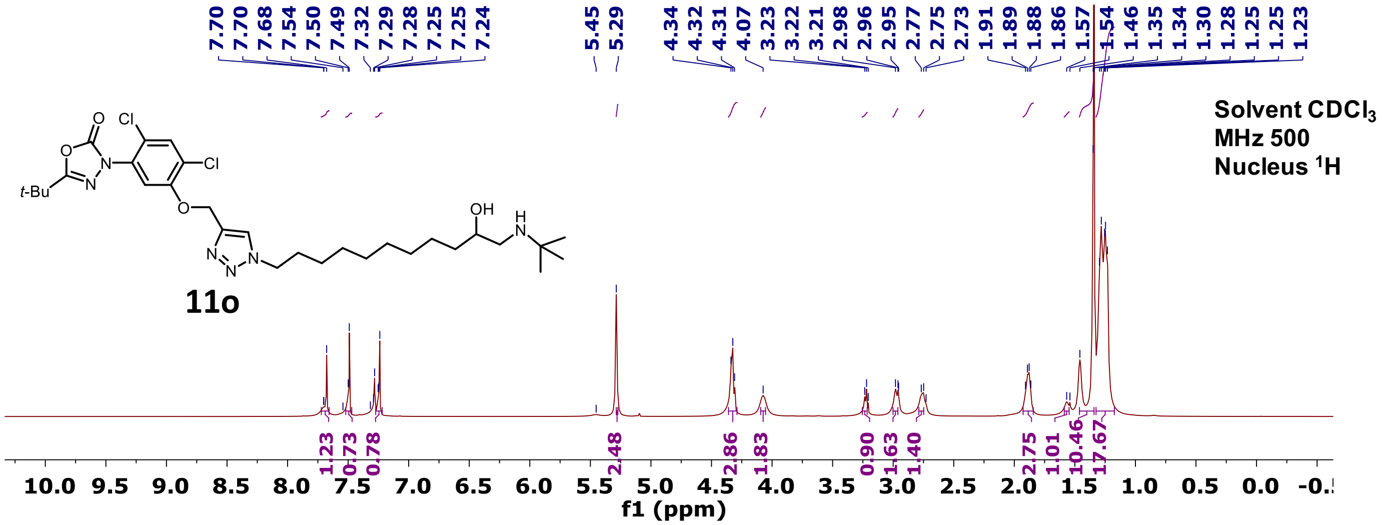

7.70 7.70 7.68 7.54 7.50 7.49 7.32 7.29 7.28 7.25 7.25 7.24 5.45 5.29 4.34 4.32 4.31 4.07 3.23 3.22 3.21 2.98 2.96 2.95 2.77 2.75 2.73 1.91 1.89 1.88 1.86 1.57 1.54 1.46 1.35 1.34 1.30 1.28 1.25 1.23
Solvent CDCl3
MHz 500
Nucleus 1H
Cl
Cl
O
N
N
t-Bu
O
O
N
N
N
OH
H
N
11o
1.23 0.73 0.78 2.48 2.86 1.83 0.90 1.63 1.40 2.75 1.01 10.46 17.67
f1 (ppm)

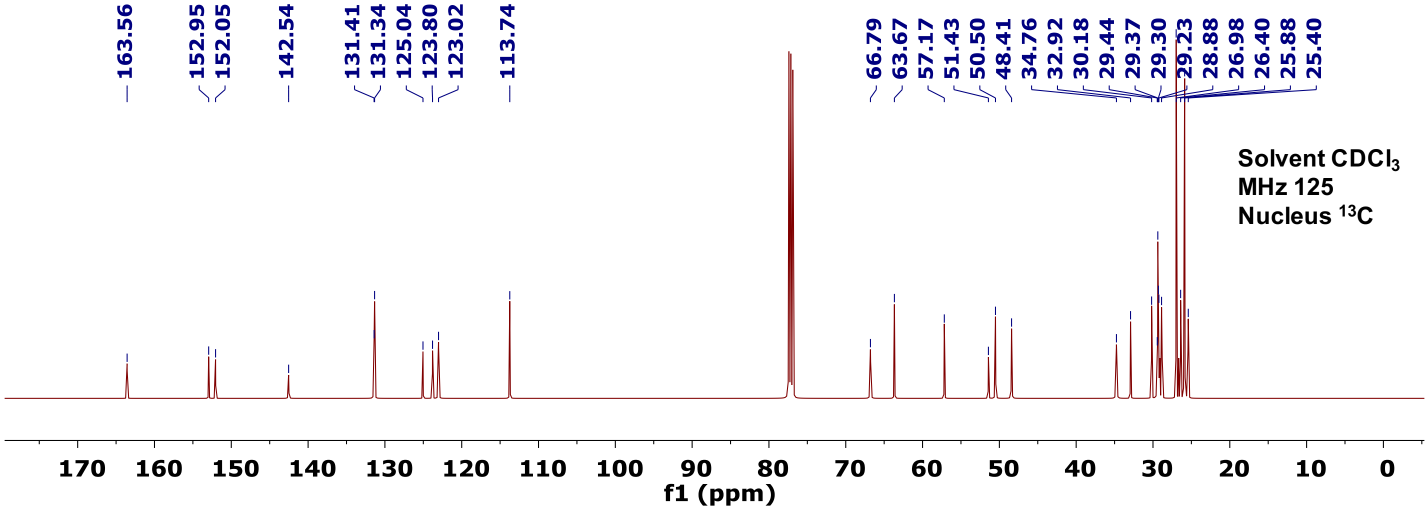

163.56 152.95 152.05 142.54 131.41 131.34 125.04 123.80 123.02 113.74 66.79 63.67 57.17 51.43 50.50 48.41 34.76 32.92 30.18 29.44 29.37 29.30 29.23 28.88 26.98 26.40 25.88 25.40
Solvent CDCl3
MHz 125
Nucleus 13C
f1 (ppm)

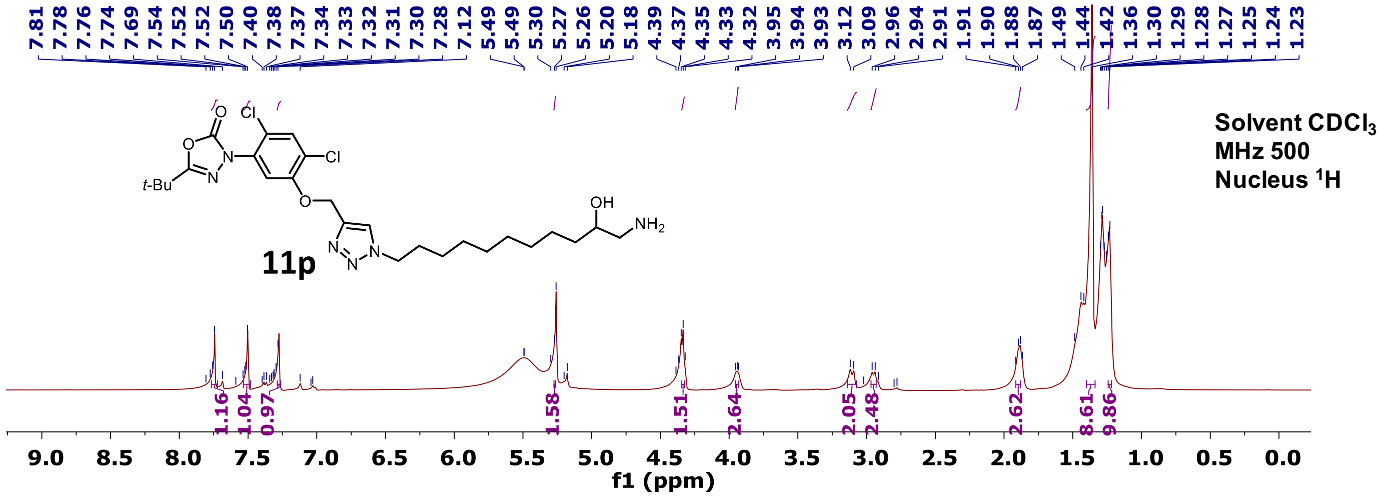
7.81 7.78 7.76 7.74 7.69 7.54 7.52 7.52 7.50 7.40 7.38 7.37 7.34 7.33 7.32 7.31 7.30 7.28 7.12 5.49 5.49 5.30 5.27 5.26 5.20 5.18 4.39 4.37 4.35 4.33 4.32 3.95 3.94 3.93 3.12 3.09 2.96 2.94 2.91 1.91 1.90 1.88 1.87 1.49 1.44 1.42 1.36 1.30 1.29 1.28 1.27 1.25 1.24 1.23
Cl
O
N
Cl
t-Bu
O
OH
NH2
N
11p
Solvent CDCl3
MHz 500
Nucleus 1H
1.16 1.04 0.97 1.58 1.51 2.64 2.05 2.48 2.62 8.61 9.86
9.0 8.5 8.0 7.5 7.0 6.5 6.0 5.5 5.0 4.5 4.0 3.5 3.0 2.5 2.0 1.5 1.0 0.5 0.0
f1 (ppm)

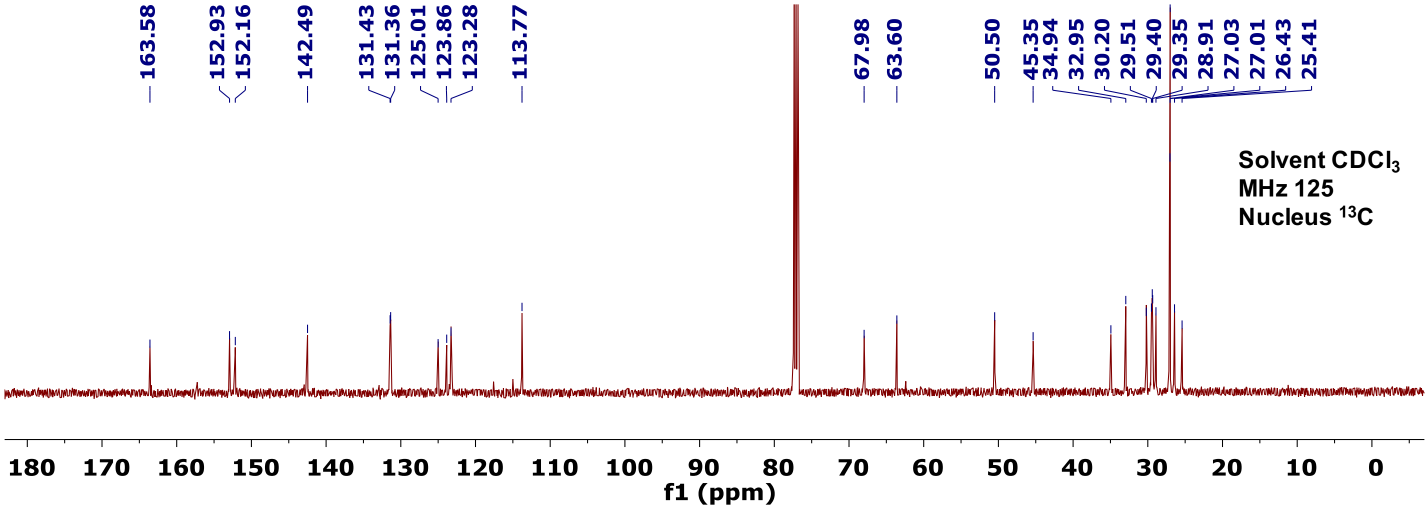
163.58 152.93 152.16 142.49 131.43 131.36 125.01 123.86 123.28 113.77 67.98 63.60 50.50 45.35 34.94 32.95 30.20 29.51 29.40 29.35 28.91 27.03 27.01 26.43 25.41
Solvent CDCl3
MHz 125
Nucleus 13C
180 170 160 150 140 130 120 110 100 90 80 70 60 50 40 30 20 10 0
f1 (ppm)

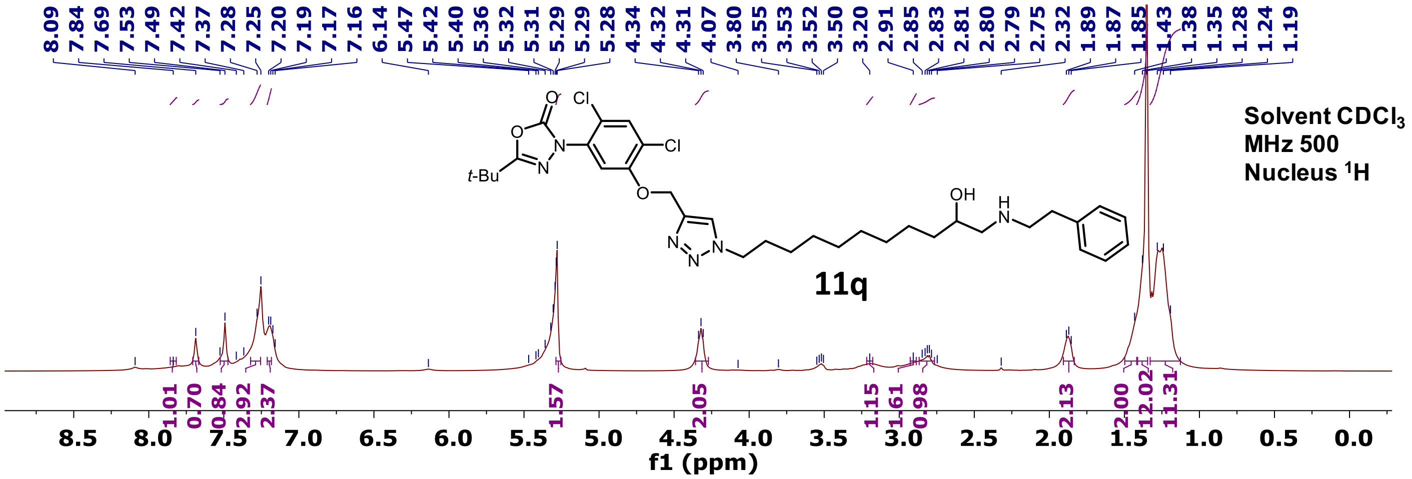
8.09 7.84 7.69 7.53 7.49 7.42 7.37 7.28 7.25 7.20 7.19 7.17 7.16 6.14 5.47 5.42 5.40 5.36 5.32 5.31 5.29 5.29 5.28 4.34 4.32 4.31 4.07 3.80 3.55 3.53 3.52 3.50 3.20 2.91 2.85 2.83 2.81 2.80 2.79 2.75 2.32 1.89 1.87 1.85 1.43 1.38 1.35 1.28 1.24 1.19
Cl
Cl
O
O
N
N
t-Bu
O
OH
H
N
N
N
N
11q
Solvent $CDCl_3$
MHz 500
Nucleus ^1H
1.01 0.70 0.84 2.92 2.37 1.57 2.05 1.15 1.61 0.98 2.13 2.00 12.02 11.31
8.5 8.0 7.5 7.0 6.5 6.0 5.5 5.0 4.5 4.0 3.5 3.0 2.5 2.0 1.5 1.0 0.5 0.0
f1 (ppm)

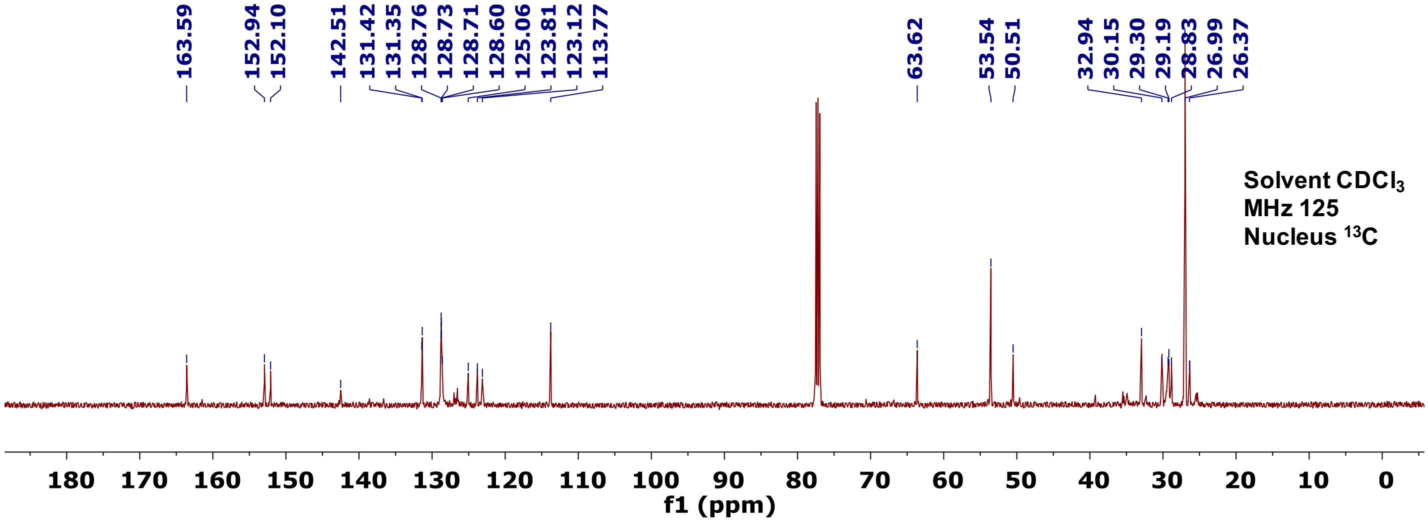
163.59 152.94 152.10 142.51 131.42 131.35 128.76 128.73 128.71 128.60 125.06 123.81 123.12 113.77 63.62 53.54 50.51 32.94 30.15 29.30 29.19 28.83 26.99 26.37
Solvent $CDCl_3$
MHz 125
Nucleus ^{13}C
180 170 160 150 140 130 120 110 100 90 80 70 60 50 40 30 20 10 0
f1 (ppm)

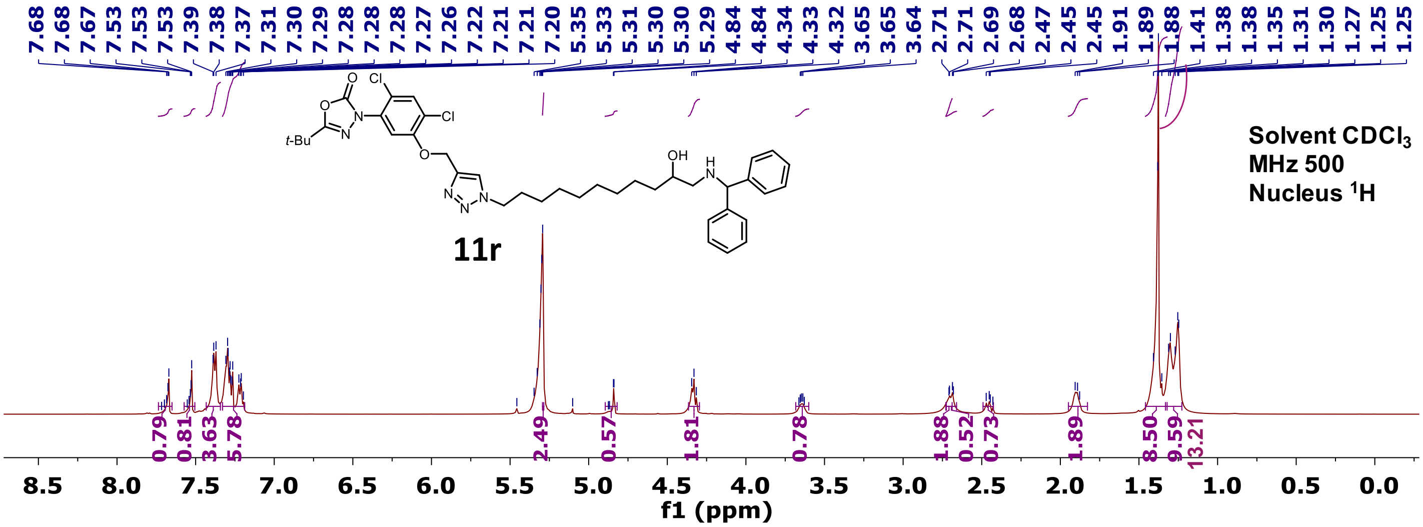
7.68 7.68 7.67 7.53 7.53 7.53 7.39 7.38 7.37 7.31 7.30 7.29 7.28 7.28 7.28 7.27 7.26 7.22 7.21 7.21 7.20 5.35 5.33 5.31 5.30 5.30 5.29 4.84 4.84 4.34 4.33 4.32 3.65 3.65 3.64 2.71 2.71 2.69 2.68 2.47 2.45 2.45 1.91 1.89 1.88 1.41 1.38 1.38 1.35 1.31 1.30 1.27 1.25 1.25
11r
t-Bu
OH
Cl
Solvent CDCl3
MHz 500
Nucleus 1H
0.79 0.81 3.63 5.78 2.49 0.57 1.81 0.78 1.88 0.52 0.73 1.89 8.50 9.59 13.21
8.5 8.0 7.5 7.0 6.5 6.0 5.5 5.0 4.5 4.0 3.5 3.0 2.5 2.0 1.5 1.0 0.5 0.0
f1 (ppm)

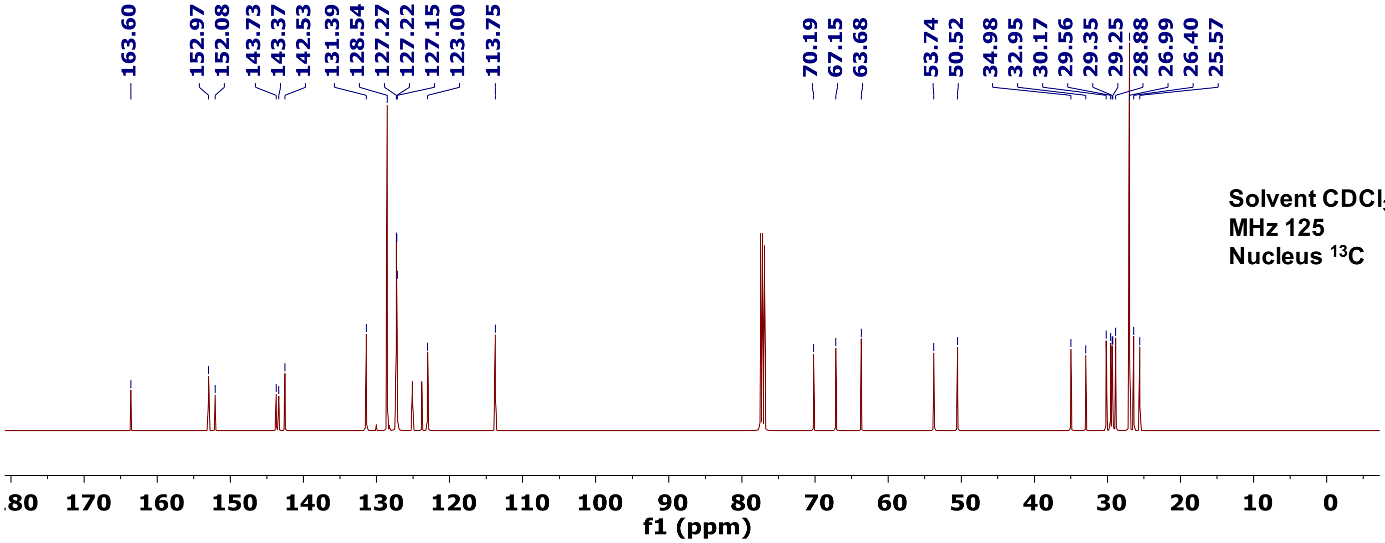
163.60 152.97 152.08 143.73 143.37 142.53 131.39 128.54 127.27 127.22 127.15 123.00 113.75 70.19 67.15 63.68 53.74 50.52 34.98 32.95 30.17 29.56 29.35 29.25 28.88 26.99 26.40 25.57
Solvent CDCl3
MHz 125
Nucleus 13C
180 170 160 150 140 130 120 110 100 90 80 70 60 50 40 30 20 10 0
f1 (ppm)

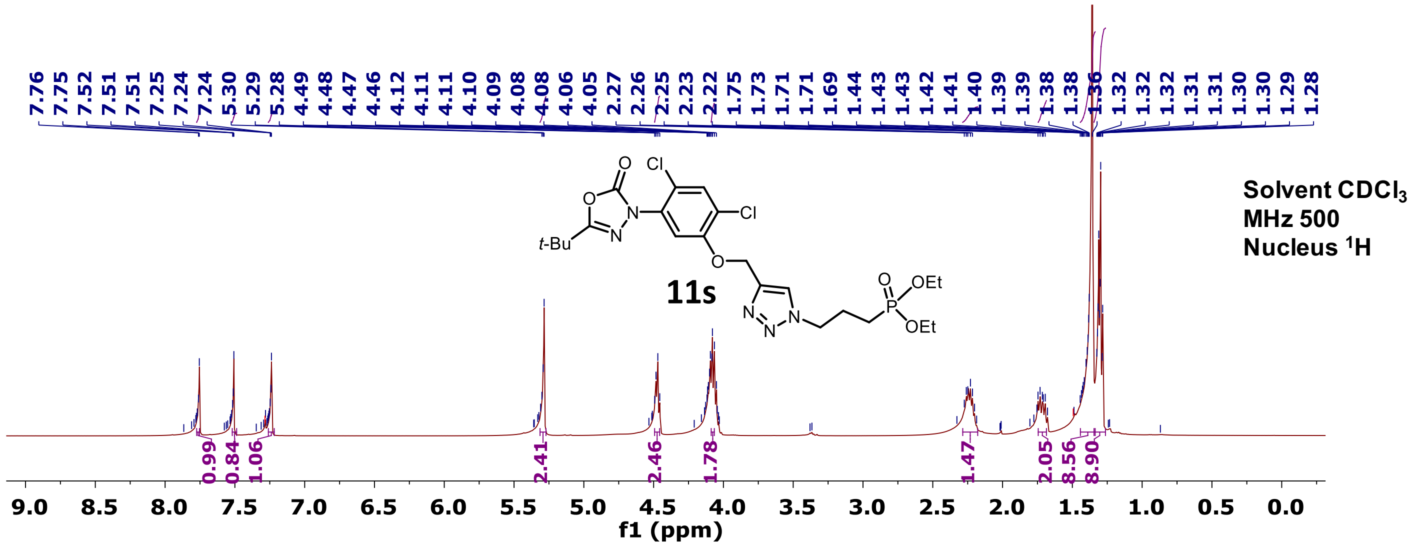
7.76
7.75
7.52
7.51
7.51
7.25
7.24
7.24
5.30
5.29
5.28
4.49
4.48
4.47
4.46
4.12
4.11
4.11
4.10
4.09
4.08
4.08
4.06
4.05
2.27
2.26
2.25
2.23
2.22
1.75
1.73
1.71
1.71
1.69
1.44
1.43
1.43
1.42
1.41
1.40
1.39
1.39
1.38
1.38
1.36
1.32
1.32
1.32
1.31
1.31
1.30
1.30
1.29
1.28
11s
Solvent CDCl3
MHz 500
Nucleus 1H
0.99
0.84
1.06
2.41
2.46
1.78
1.47
2.05
8.56
8.90
f1 (ppm)

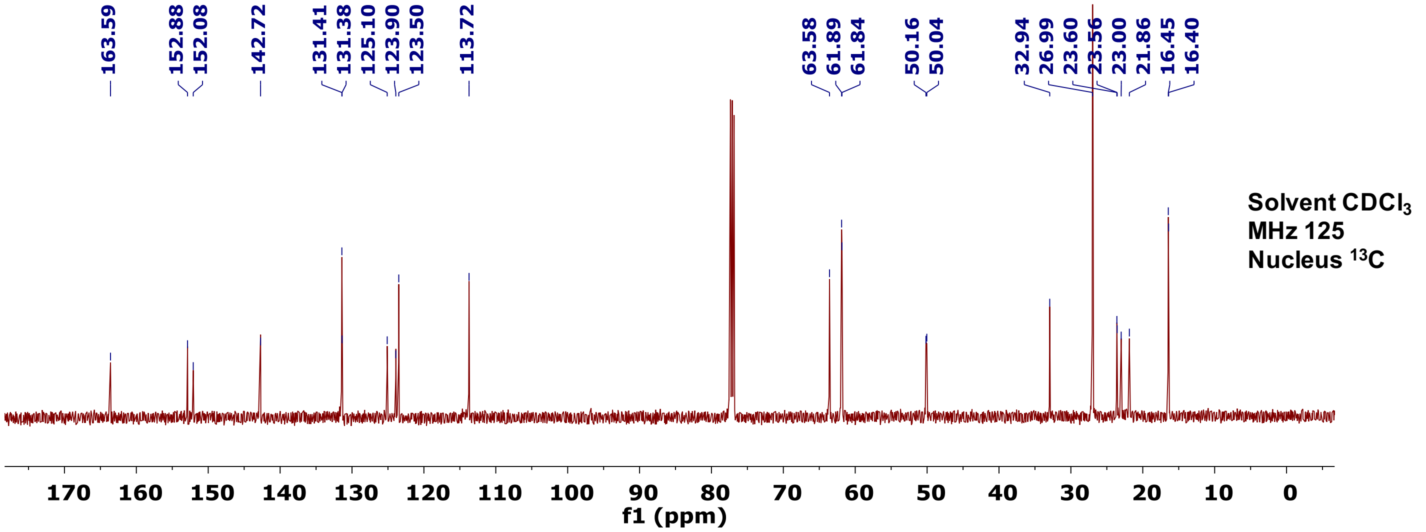
163.59
152.88
152.08
142.72
131.41
131.38
125.10
123.90
123.50
113.72
63.58
61.89
61.84
50.16
50.04
32.94
26.99
23.60
23.56
23.00
21.86
16.45
16.40
Solvent CDCl3
MHz 125
Nucleus 13C
f1 (ppm)

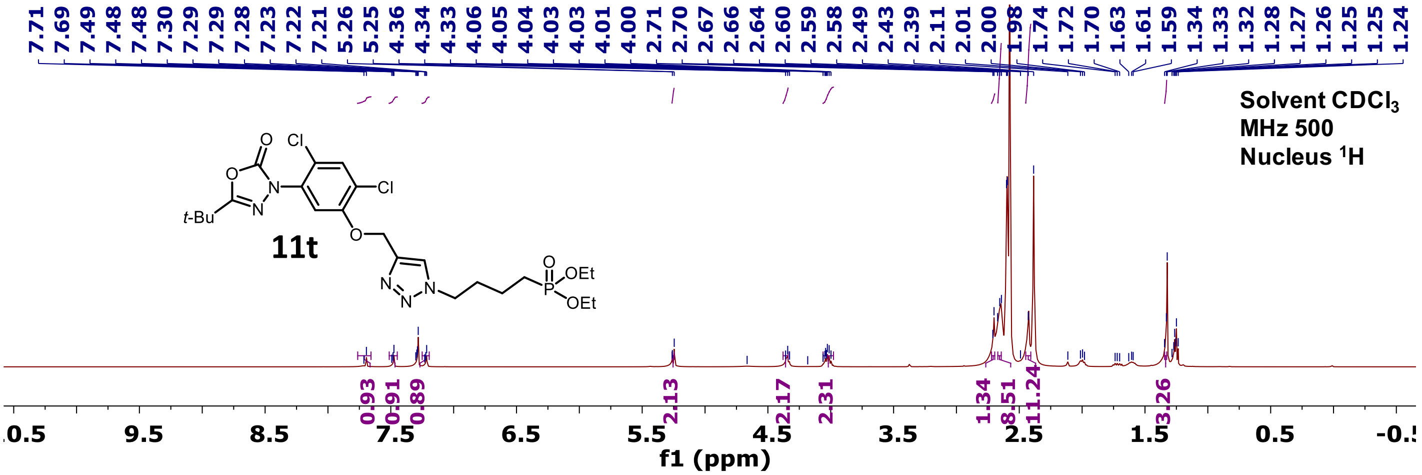
7.71 7.69 7.49 7.48 7.48 7.30 7.29 7.29 7.28 7.23 7.22 7.21 5.26 5.25 4.36 4.34 4.33 4.06 4.05 4.04 4.03 4.03 4.01 4.00 2.71 2.70 2.67 2.66 2.64 2.60 2.59 2.58 2.49 2.43 2.39 2.11 2.01 2.00 1.98 1.74 1.72 1.70 1.63 1.61 1.59 1.34 1.33 1.32 1.28 1.27 1.26 1.25 1.25 1.24
Solvent CDCl3
MHz 500
Nucleus 1H
11t
t-Bu
Cl
Cl
OEt
OEt
0.93 0.91 0.89 2.13 2.17 2.31 1.34 8.51 11.24 3.26
f1 (ppm)

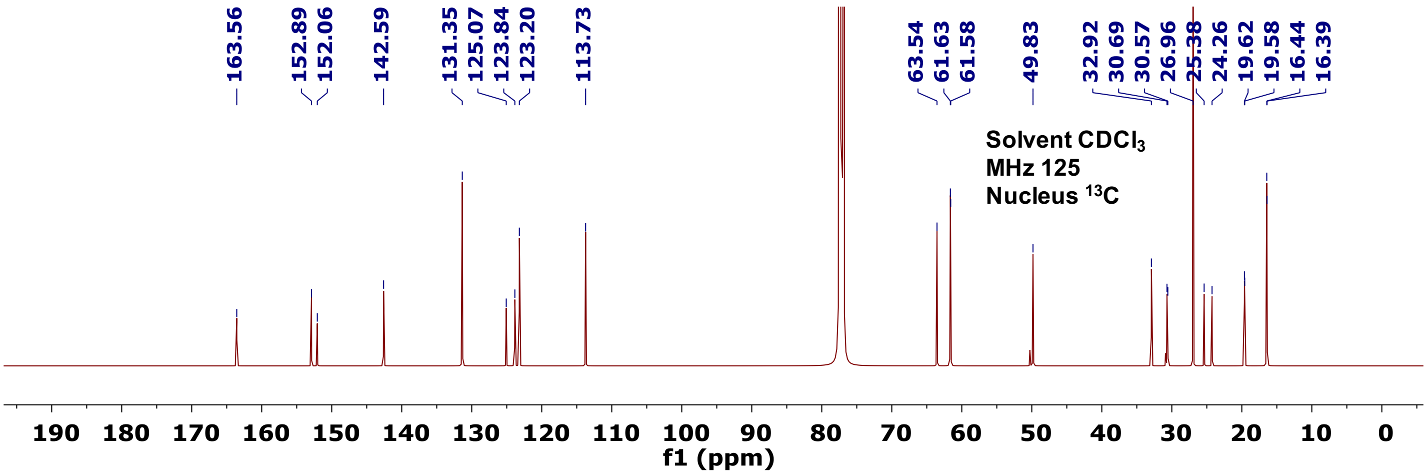
163.56 152.89 152.06 142.59 131.35 125.07 123.84 123.20 113.73 63.54 61.63 61.58 49.83 32.92 30.69 30.57 26.96 25.38 24.26 19.62 19.58 16.44 16.39
Solvent CDCl3
MHz 125
Nucleus 13C
f1 (ppm)

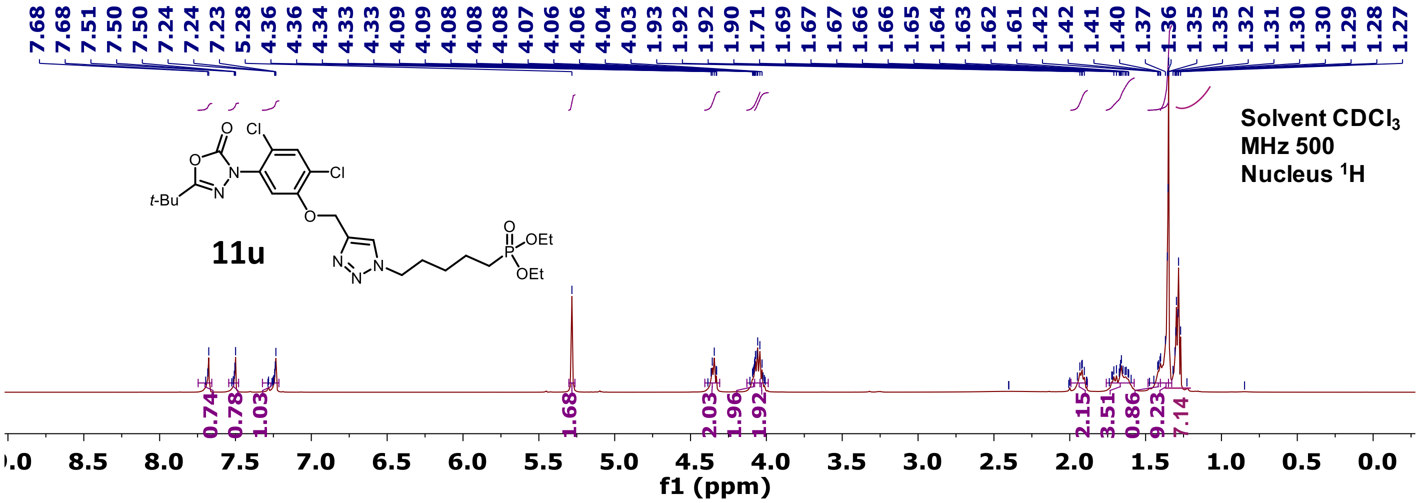
7.68 7.68 7.51 7.50 7.50 7.24 7.24 7.23 5.28 4.36 4.36 4.34 4.33 4.33 4.09 4.09 4.08 4.08 4.08 4.07 4.06 4.06 4.04 4.03 1.93 1.92 1.92 1.90 1.71 1.69 1.67 1.67 1.66 1.66 1.65 1.64 1.63 1.62 1.61 1.42 1.42 1.41 1.40 1.37 1.36 1.35 1.35 1.32 1.31 1.30 1.30 1.29 1.28 1.27
Solvent CDCl3
MHz 500
Nucleus 1H
11u
t-Bu
OEt
OEt
0.74 0.78 1.03 1.68 2.03 1.96 1.92 2.15 3.51 0.86 9.23 7.14
f1 (ppm)

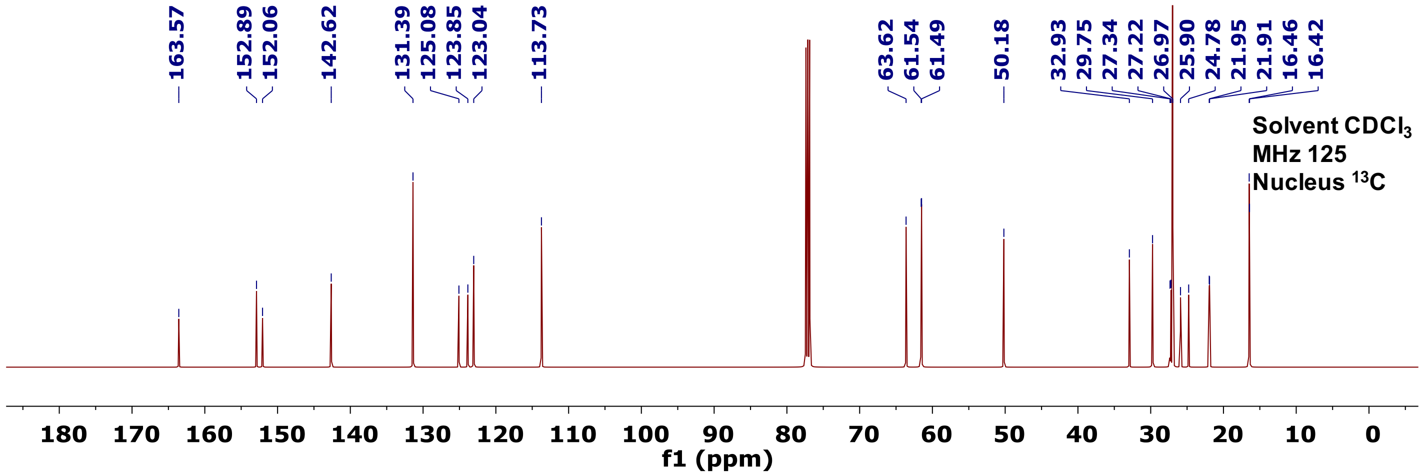
163.57 152.89 152.06 142.62 131.39 125.08 123.85 123.04 113.73 63.62 61.54 61.49 50.18 32.93 29.75 27.34 27.22 26.97 25.90 24.78 21.95 21.91 16.46 16.42
Solvent CDCl3
MHz 125
Nucleus 13C
f1 (ppm)

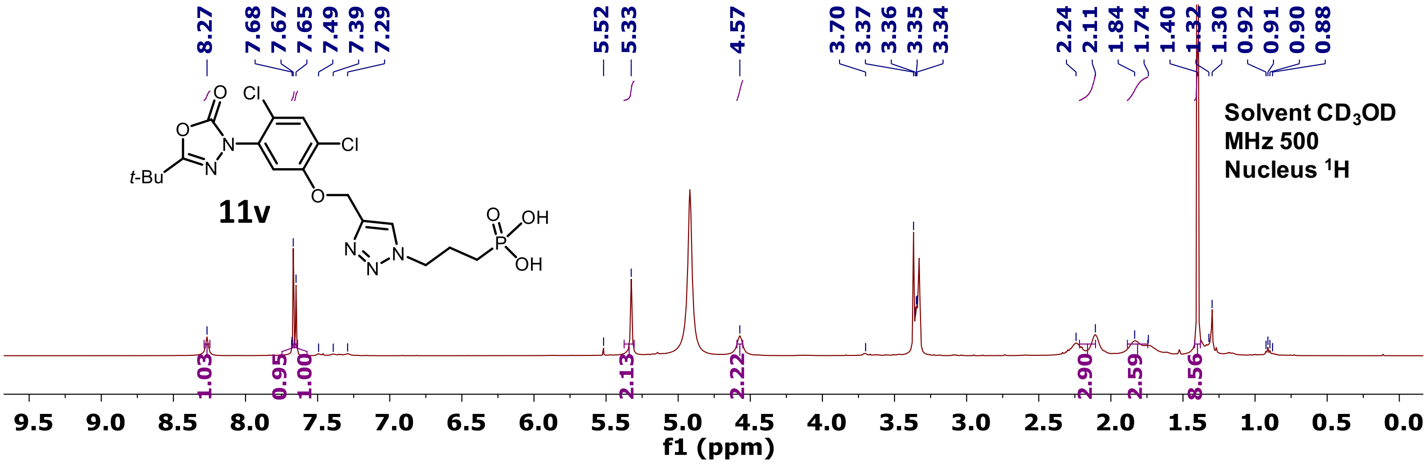
8.27
7.68
7.67
7.65
7.49
7.39
7.29
5.52
5.33
4.57
3.70
3.37
3.36
3.35
3.34
2.24
2.11
1.84
1.74
1.40
1.32
1.30
0.92
0.91
0.90
0.88
Cl
O
N
Cl
t-Bu
11v
O
OH
P
OH
N
Solvent CD_3OD
MHz 500
Nucleus ^1H
1.03
0.95
1.00
2.13
2.22
2.90
2.59
8.56
9.5 9.0 8.5 8.0 7.5 7.0 6.5 6.0 5.5 5.0 4.5 4.0 3.5 3.0 2.5 2.0 1.5 1.0 0.5 0.0
f1 (ppm)

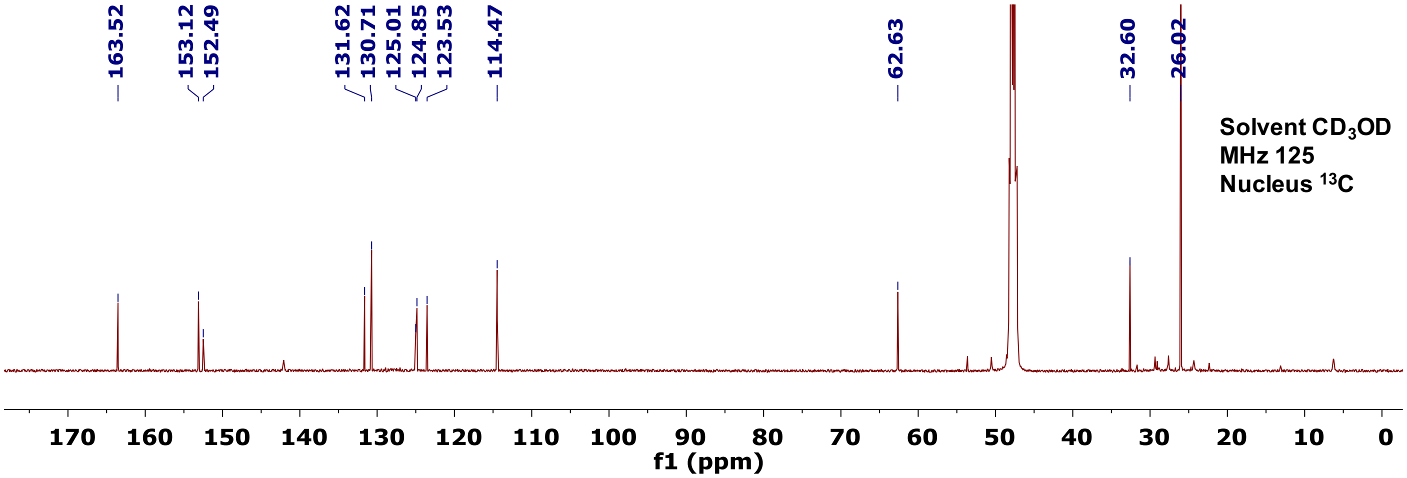
163.52
153.12
152.49
131.62
130.71
125.01
124.85
123.53
114.47
62.63
32.60
26.02
Solvent CD_3OD
MHz 125
Nucleus ^{13}C
170 160 150 140 130 120 110 100 90 80 70 60 50 40 30 20 10 0
f1 (ppm)

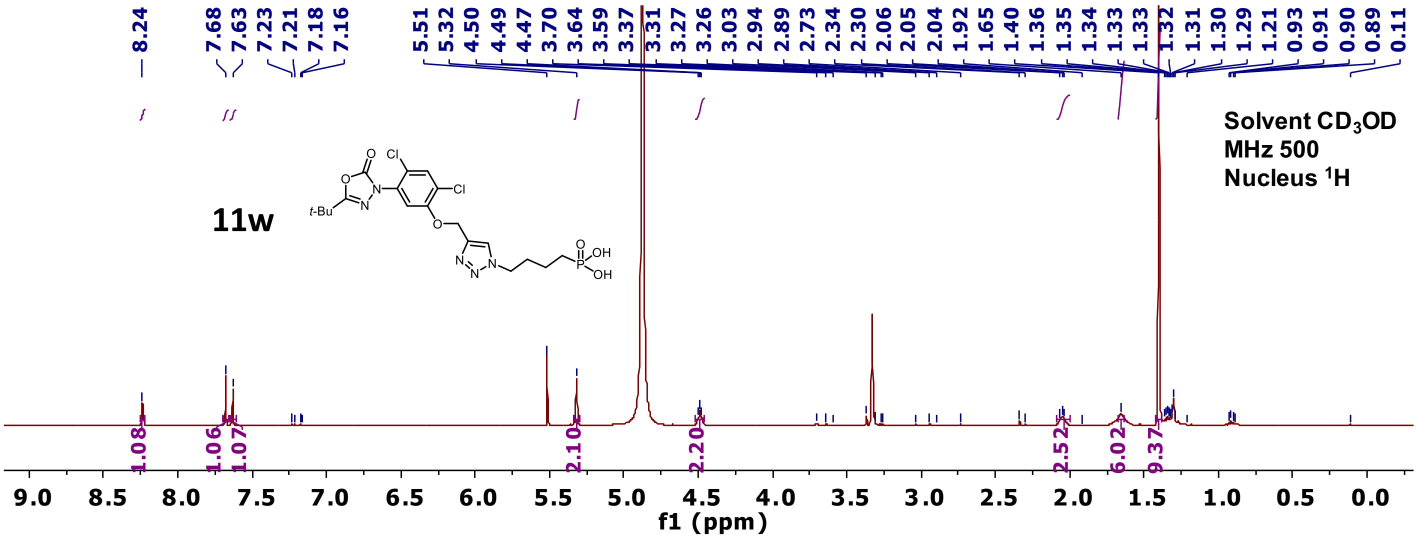
8.24
7.68
7.63
7.23
7.21
7.18
7.16
5.51
5.32
4.50
4.49
4.47
3.70
3.64
3.59
3.37
3.31
3.27
3.26
3.03
2.94
2.89
2.73
2.34
2.30
2.06
2.05
2.04
1.92
1.65
1.40
1.36
1.35
1.34
1.33
1.33
1.32
1.31
1.30
1.29
1.21
0.93
0.91
0.90
0.89
0.11
Solvent CD3OD
MHz 500
Nucleus 1H
1.08
1.06
1.07
2.10
2.20
2.52
6.02
9.37
9.0 8.5 8.0 7.5 7.0 6.5 6.0 5.5 5.0 4.5 4.0 3.5 3.0 2.5 2.0 1.5 1.0 0.5 0.0
f1 (ppm)

11w

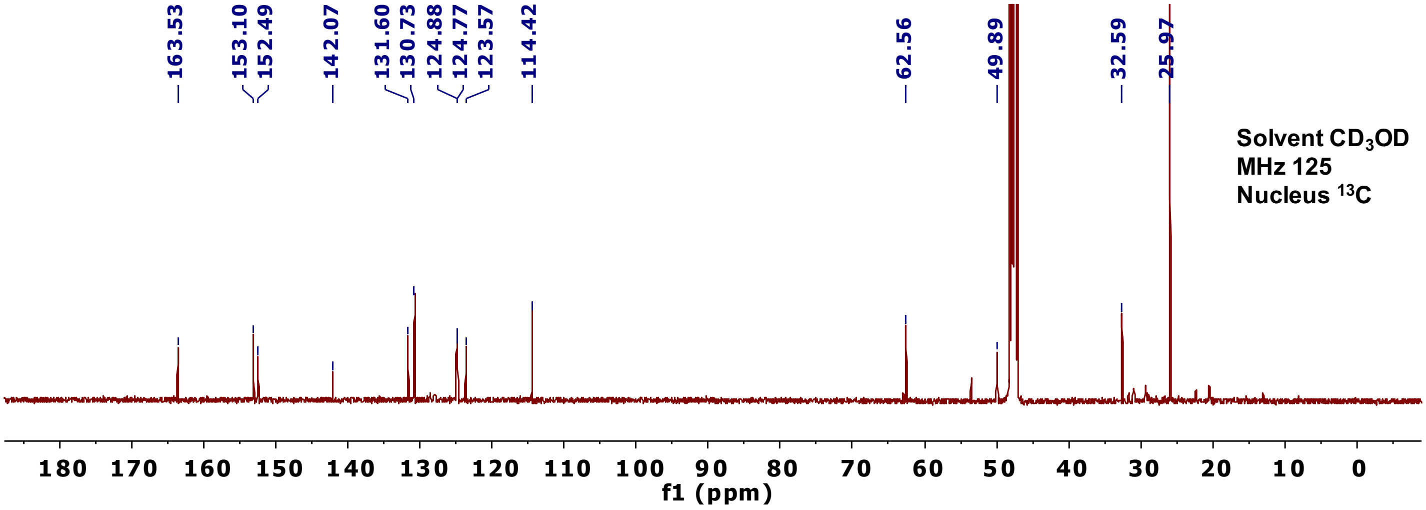
163.53
153.10
152.49
142.07
131.60
130.73
124.88
124.77
123.57
114.42
62.56
49.89
32.59
25.97
Solvent CD3OD
MHz 125
Nucleus 13C
180 170 160 150 140 130 120 110 100 90 80 70 60 50 40 30 20 10 0
f1 (ppm)
